# Supplementary material for: Population-based assessment of cardiovascular complications of rheumatic heart disease in Fiji: a record-linkage analysis
Source: BMJ Open. 2023 Apr 24;13(4):e070629. doi: 10.1136/bmjopen-2022-070629 (PMC10152053; doi:10.1136/bmjopen-2022-070629)
Supplement: Supplementary data [file bmjopen-2022-070629supp001.pdf]

Supplementary Material

Parks T, *et al. BMJ Open* 2023; 13:e070629. doi: 10.1136/bmjopen-2022-070629

Figure S1: **Overview of the study design.** Our primary analyses focused on the subset of patients from our previous reported study with clinically apparent RHD.[14] For further analyses, we defined two subsets: patients with at least one hospital attendance during follow-up, termed the hospital cohort, and women with at least one pregnancy during follow-up, termed the maternity cohort.

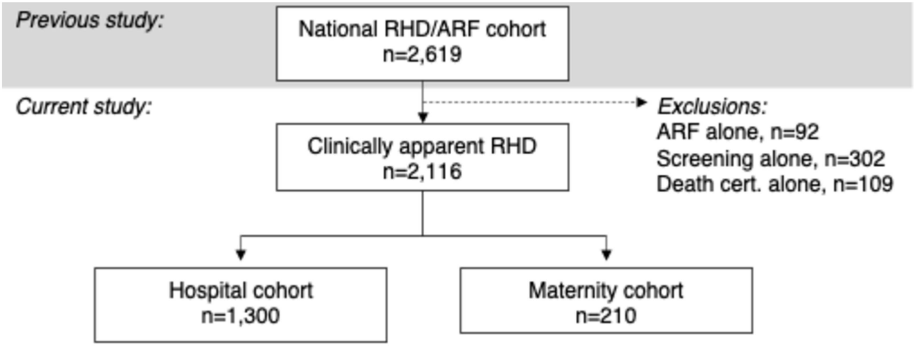

534 ARF, acute rheumatic fever.

Figure S2: **Cumulative incidence of any complication, heart failure, atrial fibrillation, ischaemic stroke and infective endocarditis in the national cohort.**

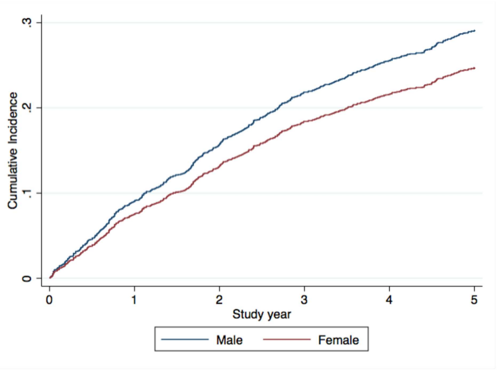

(a) All events

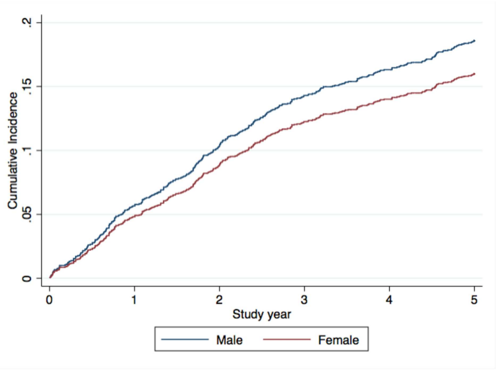

(b) Heart failure

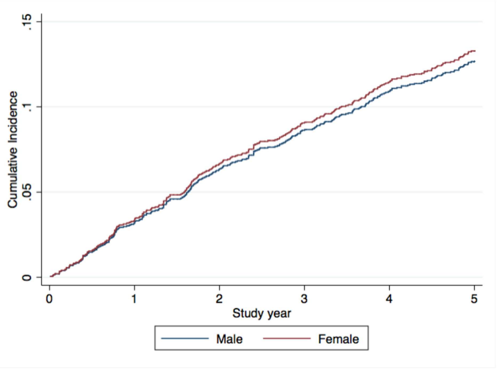

(c) Atrial fibrillation

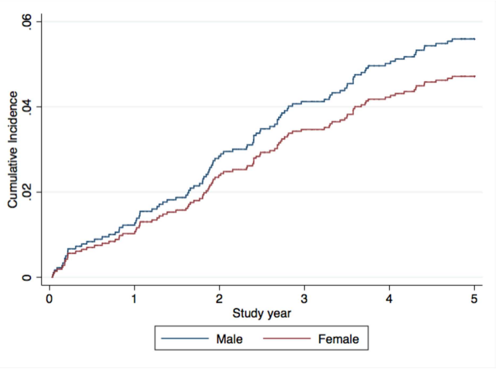

(d) Ischaemic stroke

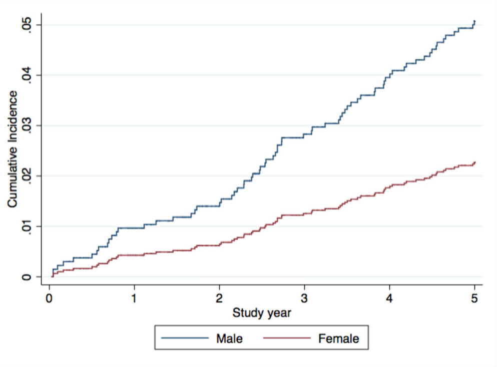

(e) Infective endocarditis

Figure S3: **Population-based rates of RHD-attributable disability from the study compared to estimates for the same period from the Global Burden of Disease (GBD) study.**[7,9,12]

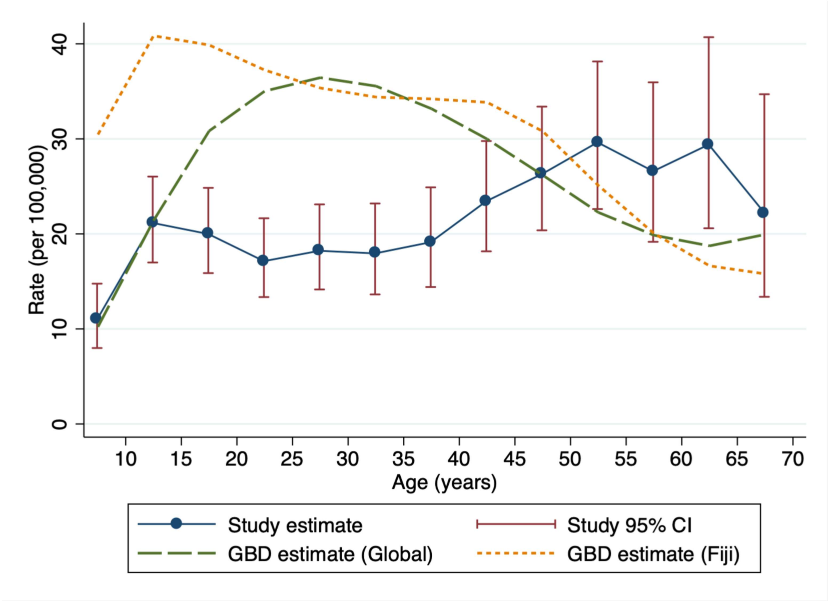

(a) Years lived with disability (YLDs)

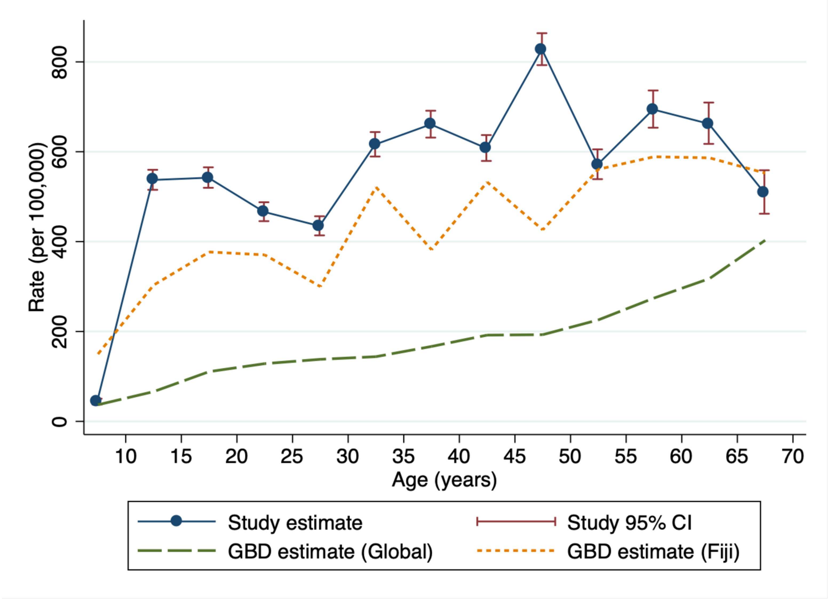

(b) Disability adjusted life-years (DALYs)

Parks T, *et al. BMJ Open* 2023; 13:e070629. doi: 10.1136/bmjopen-2022-070629

Figure S4: **Proportion of the hospital cohort with new RHD complications at 2 years follow-up compared to clinical outcomes at 2 years follow-up in the REMEDY cohort.**[15]

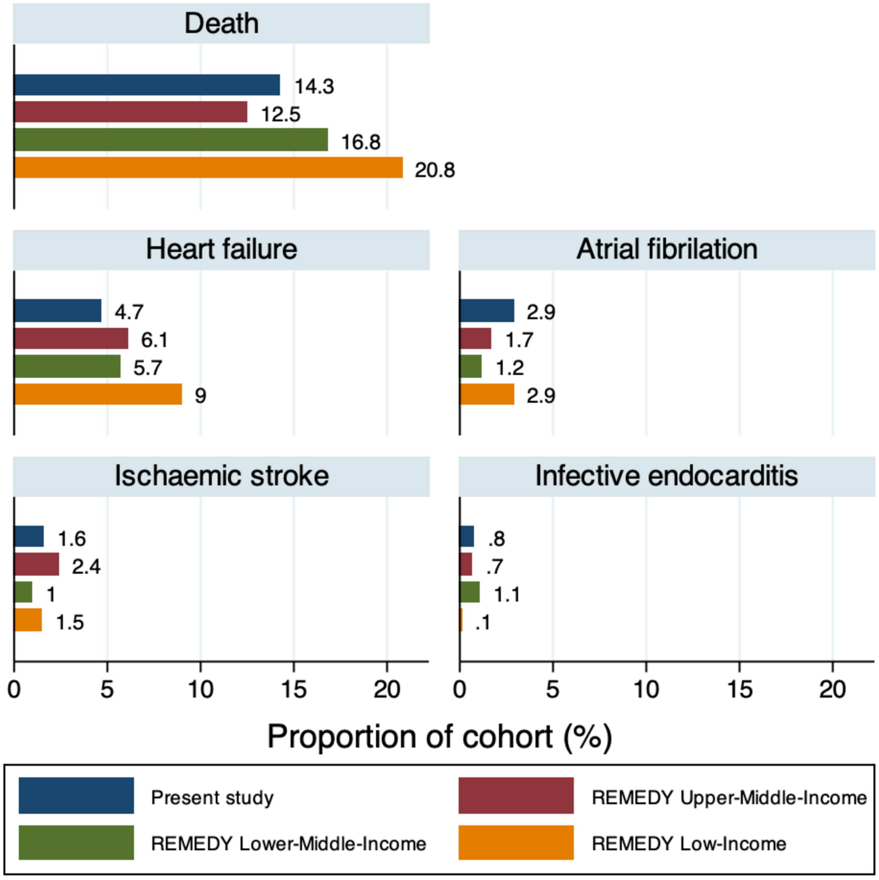

Parks T, *et al. BMJ Open* 2023; 13:e070629. doi: 10.1136/bmjopen-2022-070629

Table S1: **Diagnostic codes used for this analysis for rheumatic heart disease and its complications.**

**Diagnosis**	ICD-10 Codes*
Rheumatic heart disease	I05-I09
Acute rheumatic fever	I00-I02
Heart failure	I50
Atrial fibrillation	I48
Infective endocarditis	I33
Ischaemic stroke	G46, I63-64, I67.9, I69.3-I69.4, I69.8

*International Statistical Classification of Diseases and Related Health Problems 10th Revision (ICD-10) codes.

Table S2: **Numbers of individuals and admissions for which diagnostic codes were used in the cohort by age and sex*.** The percentage of individuals and admissions within each age category to which the code applies is also shown.

**Diagnosis**	**Age (yrs)**	**Admissions, n (%)**		**Individuals, n (%)**	
		Males n=1053	Females n=1830	Males n=892	Females n=1215
RHD	5-14	182 (60)	189 (66)	136 (38)	134 (42)
	15-39	186 (56)	437 (44)	128 (42)	273 (48)
	40-69	128 (30)	205 (38)	100 (44)	141 (46)
ARF	5-14	43 (14)	54 (18)	40 (12)	45 (14)
	15-39	7 (2)	17 (2)	7 (2)	16 (2)
	40-69	1 (0)	1 (0)	1 (0)	1 (0)
Heart failure	5-14	30 (10)	30 (10)	16 (4)	21 (6)
	15-39	90 (28)	93 (10)	53 (16)	66 (12)
	40-69	211 (50)	197 (36)	88 (40)	102 (32)
Atrial fibrillation	5-14	4 (2)	4 (2)	3 (0)	4 (2)
	15-39	79 (24)	74 (8)	46 (14)	51 (8)
	40-69	118 (28)	228 (42)	61 (28)	114 (36)
Ischaemic stroke	5-14	3 (2)	2 (0)	3 (0)	2 (0)
	15-39	16 (4)	24 (2)	15 (4)	20 (4)
	40-69	36 (8)	46 (8)	26 (12)	33 (10)
Endocarditis	5-14	18 (6)	8 (2)	16 (4)	8 (2)
	15-39	20 (6)	17 (2)	20 (6)	14 (2)
	40-69	8 (2)	5 (0)	8 (4)	5 (2)
Ischaemic heart diseas	5-14	6 (2)	2 (0)	6 (2)	2 (0)
	15-39	13 (4)	9 (0)	12 (4)	9 (0)
	40-69	142 (34)	88 (16)	70 (32)	59 (18)
Community acquired pneumonia	5-14	22 (8)	31 (10)	18 (6)	26 (8)
	15-39	29 (8)	43 (4)	26 (8)	35 (6)
	40-69	34 (8)	32 (6)	29 (12)	25 (8)
Diabetes mellitus	5-14	0 (0)	0 (0)	0 (0)	0 (0)
	15-39	2 (0)	6 (0)	1 (0)	4 (0)
	40-69	62 (14)	90 (16)	33 (14)	45 (14)
Obstetric	5-14	0 (0)	4 (2)	0 (0)	3 (0)
	15-39	0 (0)	447 (44)	0 (0)	215 (38)
	40-69	0 (0)	10 (2)	0 (0)	4 (2)

*Sex information missing for nine patients who had a total of 13 admissions.

Table S3: **Clinical events by sex in the national, hospital and maternity cohorts.***

	**National** (n = 2,116)		**Hospital** (n = 1,300)		**Maternity** (n = 210)
	Male (n = 892)	Female (n = 1,215)	Male (n = 547)	Female (n = 748)	Female (n = 210)
Hospital admission, n (%)					
Any complication	252 (28.3)	292 (24.0)	208 (38.0)	241 (32.2)	22 (10.5)
Heart failure	160 (17.9)	189 (15.6)	128 (23.4)	156 (20.9)	11 (5.2)
Atrial fibrillation	109 (12.6)	158 (13.0)	90 (16.5)	125 (16.7)	7 (3.3)
Ischaemic stroke	48 (5.4)	56 (4.6)	37 (6.8)	45 (6.3)	7 (3.3)
Infective endocarditis	45 (5.0)	27 (2.2)	40 (7.3)	23 (3.1)	4 (1.9)
Echocardiography, n (%)					
Moderate disease	103 (11.6)	162 (13.3)	81 (14.8)	137 (18.3)	45 (21.4)
Severe disease	112 (12.6)	147 (12.1)	100 (18.3)	122 (16.3)	29 (13.8)
Mitral stenosis	136 (15.3)	269 (22.1)	121 (22.1)	230 (30.8)	64 (30.5)
Heart valve surgery, n (%)					
Prior to study	32 (3.6)	54 (4.4)	26 (4.8)	38 (5.1)	7 (3.3)
During follow-up	51 (5.7)	75 (6.2)	39 (7.1)	56 (7.5)	10 (4.8)
Date uncertain	31 (3.5)	32 (2.6)	21 (3.8)	21 (2.8)	5 (2.4)
Death during follow-up, n (%)	147 (16.5)	173 (14.2)	113 (20.7)	127 (17.0)	11 (5.2)

*Sex information missing for nine patients in the national cohort and five patients in the hospital cohort.

Parks T, *et al. BMJ Open* 2023; 13:e070629. doi: 10.1136/bmjopen-2022-070629

Table S4: **Cumulative incidence rates per 100 person-years with 95% confidence intervals of nonfatal complications of RHD in the national cohort at various time intervals during the study.**

Age	Sex	Years	Any complication*	Heart failure	Atrial fibrillation	Ischaemic stroke	Infective endocarditis
5-14 years	Male	1-3	17.4 (14.5–21.0)	0.6 (0.3–1.5)	0.1 (0.0–0.9)	0.2 (0.1–1.0)	0.8 (0.3–1.7)
		4-5	8.4 (5.5–12.8)	1.2 (0.5–2.9)	0 (0.0-0.0)	0 (0.0-0.0)	1.2 (0.5–2.9)
		All	14.9 (12.5–17.7)	1.8 (0.4–1.5)	0.1 (0.0–0.6)	0.2 (0.0–0.7)	0.9 (0.5–1.6)
	Female	1-3	16.5 (13.5–20.0)	1.5 (0.8–2.7)	0.3 (0.1–1.1)	0.4 (0.1–1.3)	0.7 (0.3–1.6)
		4-5	11.4 (7.8–16.7)	0.8 (0.3–2.5)	0.3 (0.0–1.8)	0 (0.0-0.0)	0.3 (0.0–1.9)
		All	15.1 (12.7–17.9)	1.3 (0.8–2.1)	0.3 (0.1–0.8)	0.3 (0.1–0.8)	0.5 (0.2–1.2)
15-39 years	Male	1-3	13.2 (11.0–15.9)	3.6 (2.6–5.0)	3.1 (2.1–4.4)	1.7 (1.0–2.7)	0.9 (0.5–1.8)
		4-5	11.4 (8.7–14.9)	2.7 (1.7–4.2)	2.1 (1.2–3.5)	0.4 (0.1–1.3)	2.0 (1.2–3.4)
		All	12.6 (10.8–14.6)	3.2 (2.4–4.2)	2.7 (2.0–3.6)	1.1 (0.7–1.8)	1.4 (0.9–2.1)
	Female	1-3	17.1 (15.1–19.4)	3.0 (2.3–3.9)	1.6 (1.1–2.4)	0.8 (0.4–1.3)	0.7 (0.4–1.2)
		4-5	12.5 (10.1–15.5)	1.5 (0.9–2.4)	1.5 (0.9–2.4)	0.8 (0.4–1.5)	0.3 (0.1–0.8)
		All	15.6 (14.0–17.4)	2.4 (1.9–3.0)	1.6 (1.2–2.1)	0.8 (0.5–1.2)	0.5 (0.3–0.9)
40-69 years	Male	1-3	25.6 (21.4–30.5)	13.5 (10.7–16.8)	8.0 (6.0–10.6)	3.2 (2.1–5.0)	0.6 (0.2–1.7)
		4-5	20.3 (14.8–28.0)	6.5 (4.2–10.3)	6.0 (3.8–9.5)	3.0 (1.6–5.5)	1.7 (0.7–3.7)
		All	24.1 (20.6–28.1)	11.1 (9.1–13.6)	7.3 (5.7–9.3)	3.1 (2.2–4.5)	1.0 (0.5–1.9)
	Female	1-3	22.9 (19.6–26.8)	10.7 (8.7–13.3)	10.7 (8.6–13.2)	3.5 (2.5–5.0)	0.3 (0.1–1.1)
		4-5	17.2 (13.0–22.7)	4.8 (3.2–7.4)	8.6 (6.2–11.9)	2.4 (1.3–4.2)	0.6 (0.2–1.7)
		All	21.2 (18.5–24.3)	8.7 (7.2–10.5)	10.0 (8.3–11.9)	3.1 (2.3–4.2)	0.4 (0.2–0.9)

*At least one hospitalisation for heart failure, stroke, atrial fibrillation or infective endocarditis.

Table S5: **Hazard ratio estimates with 95% confidence intervals for new nonfatal complications of RHD within the national cohort in a multivariate competing-risks analysis*.**

		Any complication‡	Heart failure	Atrial fibrillation	Ischaemic stroke	Infective endocarditis
Age (yrs)	5-14	1	1	1	1	1
	15-39	**2.77 (1.44–5.32)**	1.98 (0.91–4.30)	**20.4 (5.0–83.4)**	**4.19 (1.48–11.82)**	1.20 (0.67–2.14)
	40-69	**6.78 (3.46–13.28)**	**4.41 (1.97–9.83)**	**77.4 (19.1–313.4)**	**7.26 (2.46–21.4)**	0.77 (0.34–1.74)
Sex	Male	1	1	1	1	1
	Female	**0.62 (0.44–0.87)**	0.65 (0.42–1.01)	1.05 (0.81–1.35)	0.77 (0.52–1.13)	**0.42 (0.26–0.68)**
Ethnicity	Fijian of Indian Descent	1	1	1	1	1
	iTaukei & others	**2.68 (1.79–4.03)**	**1.80 (1.09–2.96)**	**2.0 (1.52–2.61)**	**2.45 (1.52–3.94)**	1.71 (0.97–3.04)
Comorbidity§	Index = 0	1	1	1	1	1
	Index = 1	**5.92 (3.56–9.87**	**3.65 (1.89-7.02)**	**2.92 (2.03–4.21)**	**5.21 (2.59–10.5)**	1.20 (0.36–3.98)
	Index ≥2	**6.41 (4.15–9.89)**	**3.64 (2.00–6.59)**	**2.51 (1.86–3.89)**	**9.14 (5.36–15.6)**	1.97 (0.85–4.54)

∗Bold font indicates P<0.05 while underlined font indicates P<0.001. ‡At least one hospitalisation for heart failure, stroke, atrial fibrillation or endocarditis. §Modified Charlson Comorbidity Index[26] calculated without points for heart failure and stroke.

Table S6: **Population-based rates of hospitalisations for RHD complications per 100,000 person-years in the general population.**

Age (years)	Any complication*		Heart failure		Atrial fibrillation		Fiji
	Events	Rate (95% CI)	Events	Rate (95% CI)	Events	Rate (95% CI)	Popn†
0-4	0	-	0	-	0	-	84926
5-9	124	31 (25.8-37)	8	2 (0.9-3.9)	1	0.2 (0-1.4)	80020
10-14	174	41.4 (35.4-48)	21	5 (3.1-7.6)	2	0.5 (0.1-1.7)	84145
15-20	159	39.2 (33.4-45.8)	38	9.4 (6.6-12.9)	15	3.7 (2.1-6.1)	81040
20-24	181	44.3 (38.1-51.2)	34	8.3 (5.8-11.6)	26	6.4 (4.2-9.3)	81722
25-29	188	50.4 (43.4-58.1)	39	10.5 (7.4-14.3)	36	9.6 (6.8-13.4)	74621
30-34	161	49.8 (42.4-58.1)	31	9.6 (6.5-13.6)	28	8.7 (5.8-12.5)	64629
35-39	136	47.3 (39.7-56)	39	13.6 (9.6-18.5)	35	12.2 (8.5-16.9)	57501
40-44	171	59.8 (51.2-69.5)	78	27.3 (21.6-34.1)	48	16.8 (12.4-22.3)	57158
45-49	177	69.5 (59.6-80.5)	91	35.7 (28.8-43.8)	77	30.2 (23.8-37.8)	50960
50-54	147	72.6 (61.3-85.3)	66	32.6 (25.2-41.5)	63	31.1 (23.9-39.8)	40493
55-59	120	76 (63-90.9)	75	47.5 (37.4-59.5)	64	40.5 (31.2-51.8)	31584
60-64	98	80 (65-97.5)	66	53.9 (41.7-68.6)	44	35.9 (26.1-48.2)	24490
65-69	48	56.1 (41.4-74.4)	26	30.4 (19.9-44.6)	36	42.1 (29.5-58.3)	17102
Aged 0–69 years	-	45.4 (43.4-47.5)	-	14.7 (13.6-16.0)	-	11.4 (10.4-12.5)	

*All hospitalisations for heart failure, stroke, atrial fibrillation or endocarditis. †Estimated midpoint population in Fiji.

Table S6: **Continued**

Age (years)	Ischaemic stroke		Infective endocarditis		Fiji
	Events	Rate (95% CI)	Events	Rate (95% CI)	Popn*
0-4	0	-	0	-	84926
5-9	0	0 (0-0.9)	7	1.7 (0.7-3.6)	84145
10-14	3	0.7 (0.1-2.1)	9	2.1 (1-4.1)	84145
15-20	4	1 (0.3-2.5)	9	2.2 (1-4.2)	81040
20-24	7	1.7 (0.7-3.5)	3	0.7 (0.2-2.1)	81722
25-29	3	0.8 (0.2-2.3)	10	2.7 (1.3-4.9)	74621
30-34	9	2.8 (1.3-5.3)	9	2.8 (1.3-5.3)	64629
35-39	5	1.7 (0.6-4.1)	8	2.8 (1.2-5.5)	58501
40-44	17	5.9 (3.5-9.5)	8	2.8 (1.2-5.5)	57158
45-49	9	3.5 (1.6-6.7)	3	1.2 (0.2-3.4)	50960
50-54	14	6.9 (3.8-11.6)	4	2 (0.5-5.1)	40493
55-59	8	5.1 (2.2-10)	1	0.6 (0-3.5)	31584
60-64	7	5.7 (2.3-11.8)	0	0 (0-3)	24490
65-69	7	8.2 (3.3-16.9)	0	0 (0-4.3)	17102
Aged 0–69 years		2.2 (1.8-2.7)		1.7 (1.3-2.1)	

*Estimated midpoint population in Fiji.

Table S7: **Incidence rate ratios estimates with 95% confidence intervals for new nonfatal complications of RHD in the general population in multivariate Poisson regression*.**

		Any complication†	Heart failure	Atrial fibrillation	Ischaemic stroke	Infective endocarditis
Age (yrs)	5-14	1	1	1	1	1
	15-39	2.00 (1.75-2.28)	**4.37 (2.95-6.48)**	**32.8 (10.4-103.6)**	**6.74 (2.04-22.3)**	**1.9 (1.04-3.46)**
	40-69	3.04 (2.66-3.48)	**16.0 (10.9-23.3)**	**130 (41.6-408)**	**25.2 (7.88-80.8)**	1.28 (0.63-2.61)
Sex	Male	1	1	1	1	1
	Female	**1.43 (1.30-1.57)**	0.93 (0.79-1.09)	**1.51 (1.25-1.82)**	1.27 (0.84-1.92)	0.63 (0.39-1.02)
Ethnicity	Fijian of Indian Descent	1	1	1	1	1
	iTaukei & others	**1.39 (1.27-1.53)**	1.18 (1.0-1.38)	**1.30 (1.07-1.59)**	**1.79 (1.13-2.84)**	**2.11 (1.18-3.75)**

*Bold font indicates P<0.05 while underlined font indicates P<0.001. †At least one hospitalisation for heart failure, stroke, atrial fibrillation or endocarditis.

Table S8: **Estimates of years of life lost (YLL), years lived with disability (YLD) and disability adjusted life years (DALYs) attributable to RHD complications per 100,000 person-years in the general population.**

Age	Deaths	Death rate	YLL	YLL rate	YLD	YLD rate	DALYs rate	Fiji popn.*	GBD. std (%)†
0-4	0	0	0	0	0	0	0	84926	10.18
5-9	1.5	0.4	130.1	32.5	43.7	10.9	43.5	80020	9.68
10-14	27.5	6.5	2171.3	516.1	88.9	21.1	537.2	84145	8.95
15-19	28.6	7.1	2116	522.2	81.3	20.1	542.3	81040	8.38
20-24	26.5	6.5	1834.3	448.9	70.4	17.2	466.1	81722	8.02
25-29	24.2	6.5	1554.3	416.6	67.8	18.2	434.8	74621	7.78
30-34	32.7	10.1	1933.1	598.2	58.3	18	616.3	64629	7.33
35-39	34	11.8	1844.8	641.6	54.7	19	660.7	57501	6.78
40-44	33.9	11.8	1670.2	584.4	66.8	23.4	607.8	57158	6.09
45-49	46	18	2042.3	801.5	66.5	26.1	827.6	50960	5.47
50-54	27.7	13.7	1097.3	542	59.9	29.6	571.5	40493	4.87
55-59	30.2	19.1	1053.7	667.2	42.2	26.7	693.9	31584	4.25
60-64	25.6	20.9	774.9	632.8	35.9	29.3	662.2	24490	3.6
65-69	16.2	19	416.1	486.7	18.6	21.8	508.4	17102	2.91
Aged 0–69 years (95% CI)		8.6 (7.7-9.5)		448.9 (442.5-455.4)		18.2 (16.9-19.5)	467.1 (460.5-473.7)		
Age-standardised‡ (95% CI)		9.4 (8.5-10.3)		438.0 (431.8-444.4)		18.4 (17.1-19.7)	456.4 (450.1-462.9)		

*Estimated midpoint population in Fiji. †GBD world population age standard[30] ; ‡Study rates for the population aged 65–69 years applied to population aged ≥70 years.

Table S9: **Hazard ratio estimates with 95% confidence intervals for risk of death associated with hospitalisation for nonfatal complications of RHD in the hospital cohort in a multivariate Cox regression analysis.**

Variable	Level	HR (95% CI)	P
Heart failure	Before	1.0	
with or without atrial fib.	After	6.57 (4.76–9.06)	<0.001
Atrial fibrillation	Before	1.0	
without heart failure	After	2.86 (1.75–4.67)	<0.001
Ischaemic stroke	Before	1.0	
	After*	6.56 (3.82–11.2)	<0.001
Infective endocarditis	Before	1.0	
	After*	4.48 (2.22–9.02)	<0.001
Age (yrs)	5-14	1.0	
	15-39	1.40 (0.88–2.24)	0.16
	40-69	1.67 (1.01–2.75)	0.046
Sex	Male	1.0	
	Female	0.83 (0.63–1.09)	0.17
Ethnicity	Fijian of Indian Descent	1.0	
	iTaukei & others	0.78 (0.57–1.07)	0.12
Comorbidity†	Index < 2	1.0	
	Index ≥2	1.49 (1.10–2.03)	0.011
Residence	Urban	1.0	
	Rural	1.18 (0.90–1.54)	0.23

*Hazard within 28 days of admission for complication. †Modified Charlson Comorbidity Index[26] calculated without points for heart failure and stroke.
