# Supplementary material for: Population-based assessment of cardiovascular complications of rheumatic heart disease in Fiji: a record-linkage analysis
Source: BMJ Open. 2023 Apr 24;13(4):e070629. doi: 10.1136/bmjopen-2022-070629 (PMC10152053; doi:10.1136/bmjopen-2022-070629)
Supplement: Author's manuscript [file bmjopen-2022-070629.draft_revisions.pdf]

BMJ Open is committed to open peer review. As part of this commitment we make the peer review history of every article we publish publicly available.

When an article is published we post the peer reviewers' comments and the authors' responses online. We also post the versions of the paper that were used during peer review. These are the versions that the peer review comments apply to.

The versions of the paper that follow are the versions that were submitted during the peer review process. They are not the versions of record or the final published versions. They should not be cited or distributed as the published version of this manuscript.

BMJ Open is an open access journal and the full, final, typeset and author-corrected version of record of the manuscript is available on our site with no access controls, subscription charges or pay-per-view fees (http://bmjopen.bmj.com).

If you have any questions on BMJ Open's open peer review process please email info.bmjopen@bmj.com

BMJ Open

Population-Based Assessment of Nonfatal Complications of Rheumatic Heart Disease in Fiji: a Record-linkage Analysis

Journal:	*BMJ Open*
Manuscript ID	bmjopen-2022-070629
Article Type:	Original research
Date Submitted by the Author:	01-Dec-2022

Complete List of Authors:	Parks, Tom; Imperial College London; University of Oxford<br>Narube, Litia; Fiji National University College of Medicine Nursing and Health Sciences<br>Perman, Mai Ling; Fiji National University College of Medicine Nursing and Health Sciences<br>Sakumeni, Kelera; Fiji Ministry of Health & Medical Services<br>Fong, James; Fiji Ministry of Health & Medical Services<br>Engelman, Daniel; Murdoch Children's Research Institute<br>Colquhoun, Samantha ; Australian National University<br>Steer, Andrew; Murdoch Children's Research Institute<br>Kado, Joseph; Fiji Ministry of Health & Medical Services; Telethon Kids Institute

Keywords:	Valvular heart disease < CARDIOLOGY, Heart failure < CARDIOLOGY, Stroke < NEUROLOGY, PAEDIATRICS, OBSTETRICS, PUBLIC HEALTH

SCHOLARONE™
Manuscripts

I, the Submitting Author has the right to grant and does grant on behalf of all authors of the Work (as defined in the below author licence), an exclusive licence and/or a non-exclusive licence for contributions from authors who are: i) UK Crown employees; ii) where BMJ has agreed a CC-BY licence shall apply, and/or iii) in accordance with the terms applicable for US Federal Government officers or employees acting as part of their official duties; on a worldwide, perpetual, irrevocable, royalty-free basis to BMJ Publishing Group Ltd ("BMJ") its licensees and where the relevant Journal is co-owned by BMJ to the co-owners of the Journal, to publish the Work in this journal and any other BMJ products and to exploit all rights, as set out in our licence.

The Submitting Author accepts and understands that any supply made under these terms is made by BMJ to the Submitting Author unless you are acting as an employee on behalf of your employer or a postgraduate student of an affiliated institution which is paying any applicable article publishing charge ("APC") for Open Access articles. Where the Submitting Author wishes to make the Work available on an Open Access basis (and intends to pay the relevant APC), the terms of reuse of such Open Access shall be governed by a Creative Commons licence – details of these licences and which Creative Commons licence will apply to this Work are set out in our licence referred to above.

Other than as permitted in any relevant BMJ Author's Self Archiving Policies, I confirm this Work has not been accepted for publication elsewhere, is not being considered for publication elsewhere and does not duplicate material already published. I confirm all authors consent to publication of this Work and authorise the granting of this licence.

For peer review only

Population-Based Assessment of Nonfatal Complications of Rheumatic Heart Disease in Fiji: a Record-linkage Analysis

Tom Parks MD*[1,2], Litia Narube MBBS[3], Mai Ling Perman MBBS[3], Kelera Sakumeni MBBS[4], James J. Fong MBBS[4], Daniel Engelman PhD[5,6], Samantha M. Colquhoun PhD[7], Andrew C. Steer PhD[5,6], and Joseph Kado MBBS[4,8,9]

[1]Imperial College London, London, UK
[2]University of Oxford, Oxford, UK
[3]Fiji National University, Suva, Fiji
[4]Fiji Islands Ministry of Health and Medical Services, Suva, Fiji
[5]Murdoch Children's Research Institute, Melbourne Australia
[6]University of Melbourne, Melbourne, Australia
[7]Australian National University, Canberra, Australia
[8]Telethon Kids Institute, Perth, Australia
[9]University of Western Australia, Perth, Australia

*Correspondence to: t.parks@imperial.ac.uk

For peer review only - http://bmjopen.bmj.com/site/about/guidelines.xhtml

1 Abstract

Objective: To determine population-based rates of nonfatal complications of rheumatic heart disease (RHD).

Design: Retrospective cohort study based on multiple sources of routine clinical and administrative data amalgamated by probabilistic record-linkage.

Setting: Fiji, an upper-middle-income country, where most of the population has access to government-funded health care services.

Participants: National cohort of 2,116 patients with clinically apparent RHD aged 5-69 years during 2008-2012.

Primary and secondary outcome measures: The primary outcome was hospitalisation for any of heart failure, atrial fibrillation, ischaemic stroke and infective endocarditis. Secondary outcomes were first hospitalisation for each of the complications individually in the national cohort as well as in hospital (n=1300) and maternity (n=210) subsets. Information on outcomes was obtained from discharge diagnoses coded in the hospital patient information system. Population-based rates were obtained using relative survival methods with census data as the denominator.

Results: Among 2,116 patients in the national cohort (median age, 23.3 years; 57.7% female), 546 (25.8%) were hospitalised for an RHD complication, a substantial proportion of all cardiovascular admissions in the country during this period in those aged 0–40 years (heart failure, 210/454, 46.2%; ischaemic stroke 31/134, 23.3%). Absolute numbers of RHD complications peaked during the third decade of life with higher population-based rates in women compared to men (incidence rate ratio 1.4, 95% CI 1.3–1.6, P<0.001). Hospitalisation for any RHD complication was associated with substantially increased risk of death (hazard ratio, 5.4, 95% CI, 3.4–8.8, P<0.001), especially after the onset of heart failure (hazard ratio, 6.6, 95% CI, 4.8–9.1, P<0.001).

Conclusions: Our study defines the burden of RHD-attributable morbidity in the general population of Fiji, likely reflecting the situation in low- and middle-income countries worldwide. Hospitalisation for an RHD complication is associated with markedly increased risk of death, re-emphasising the importance of effective early prevention.

For peer review only

For peer review only - http://bmjopen.bmj.com/site/about/guidelines.xhtml

Strengths and limitations of this study

- Our study was based on probabilistic record-linkage providing an efficient means to derive population-based estimates of disease burden from routinely collected data.
- We used relative survival methods to estimate rates of RHD-attributable morbidity in the general populations since this approach helps separate out events that are unrelated to the disease of interest.
- We demonstrate the comparability of the hospital subset of our cohort to key previous hospital-based studies, including the landmark REMEDY study, set in Asia and Africa, greatly increasingly the generalisability of our population-based estimates beyond Fiji.
- The routinely collected data used in our study are likely to contain some inaccuracies, including in the precision of coding of discharge diagnoses. However, there is little reason to suspect the relevant diagnostic codes would have been used differently in patients with and without RHD.
- Reflecting external factors, we recognise that several years have now passed since the period from which our multiple sources of routine data were available. Nonetheless, they continue to provide invaluable insight into the burden of RHD in low resource settings, chiefly due to the scarcity of comparable estimates from elsewhere.

For peer review only - http://bmjopen.bmj.com/site/about/guidelines.xhtml

2 Introduction

Rheumatic heart disease (RHD) is a chronic disease of the heart valves that predominantly affects children and young adults in low- and middle-income countries (LMICs).[1] The disease is a consequence of an aberrant immune response to *Streptococcus pyogenes* infection causing inflammation and scarring of the heart valves[2], which manifests clinically through complications such as heart failure, ischaemic stroke and early death.[3] Secondary prevention is achievable using long-term antibiotic prophylaxis,[4] although implementation remains challenging in most settings where the disease is endemic.[5] Moreover, there remains no vaccine against the causative *S. pyogenes*.[6]

In recent years, there has been growing recognition that RHD causes a substantial burden of disease and disability globally,[7–10] culminating in 2018 in a World Health Assembly resolution calling for a coordinated global response.[11] Global summary estimates by the Global Burden of Disease (GBD) project suggest RHD accounts for approximately 305,000 deaths and 10.7 million disability-adjusted life years annually, the majority of these occurring LMICs.[7,9,12] Notwithstanding their importance in providing impetus for increased investment in RHD prevention, such estimates remain problematic because of the scarcity of good quality data available from LMIC settings, where the disease is endemic.[13] For example, to our knowledge, our previous record-linkage analysis set in Fiji remains the only population-based estimate of RHD-attributable mortality from an LMIC.[14] Moreover, data pertaining to the rates of nonfatal complications, such as heart failure and stroke, among RHD patients living in LMICs are particularly scarce.[7]

One compelling exception to this pattern was the landmark REMEDY study, published in 2016, a prospective, hospital-based registry that defined rates of death, heart failure and stroke among 3,343 symptomatic RHD patients enrolled across 12 African countries, India and Yemen.[15] However, as the authors of that study noted, since the patients were mostly enrolled at tertiary referral hospitals, the rates observed cannot be easily generalised to the wider pool of RHD patients away from hospital settings.[15] Furthermore, neither REMEDY nor more recent studies of RHD progression provide

For peer review only - http://bmjopen.bmj.com/site/about/guidelines.xhtml

estimates of population-based rates of complications of RHD[16–19], which are needed to improve current global summary estimates as well as to inform the design, implementation and evaluation of disease control efforts.[13,20]

Therefore we sought to assess the burden and calculate nationwide population-based rates of RHD-attributable morbidity in Fiji, a middle-income country in the Western Pacific.

3 Methods

This study investigated RHD-attributable morbidity among patients with clinically apparent RHD in Fiji during 2008-2012. Patients were identified from a cohort previously ascertained by probabilistic record linkage (Supplementary Figure S1).[14] In this analysis, we investigated the four major complications of RHD: heart failure, atrial fibrillation, ischaemic stroke and infective endocarditis[3], which together we term 'any complication'. We then used census data to calculate age-specific RHD-attributable rates, as well as estimates of years lived with disability (YLDs), for the wider population.

We also examined two subsets of the 'national' cohort in further detail (Supplementary Figure S1). The first, termed the 'hospital' cohort, comprised patients with at least one hospital attendance during follow-up, allowing assessment of the relationship between RHD-attributable morbidity and mortality in this setting, as well as comparison with previously reported hospital-based studies. The second, termed the 'maternity' cohort, comprised women with at least one diagnostic code indicating pregnancy or childbirth during follow-up, enabling a description of the RHD-attributable morbidity and mortality during pregnancy, childbirth and up to one year postpartum in the cohort.[21] The study was approved by the Fiji National Research Ethics Review Committee (2013-89) and the Oxford University Tropical Research Ethics Committee (1055-13).

For peer review only - http://bmjopen.bmj.com/site/about/guidelines.xhtml

3.1 Setting

At the time of the study, the population of Fiji was approximately 840,000, comprising two major ethnic groups, Indigenous iTaukei and Fijians of Indian Descent.[22,23] Government-funded hospital services are available free-of-charge including consultations, admissions, laboratory investigations and radiological examinations.[24] A high proportion of pregnant women give birth in hospital.[24]

3.2 Cohort

For peer review only

We used sources of data and record-linkage methods as described previously.[14] Briefly, we used a probabilistic record-linkage procedure to amalgamate data across the four datasets: a hospital patient information system, a disease control register, echocardiography clinic registers and a database of death certificates. After extensive cleaning and standardisation, we used multiple identifiers including registration numbers, names, sex, ethnicity, birth dates, and locality of residence to find pairs of records that referred to the same individual. In its final configuration, our procedure identified known duplications in the hospital patient information system with sensitivity of 91.4% and specificity of 99.9%.[14]

For this current analysis, we focussed on patients with clinically apparent RHD reported in one or more of the hospital patient information system, the disease control register and the echocardiography clinic registers. As before, we restricted the analysis to patients aged 5–69 years and the period 2008–2012 for which the most data was available.[14] We obtained information on complications resulting in hospitalisation, as well as pregnancy outcomes from the coded discharge diagnoses reported in the hospital patient information system. The completeness and quality of Fiji's hospital patient information system data have been independently investigated previously[25] and the diagnostic codes used to define RHD and its complications are summarised in Supplementary Table S1. We also used this system to obtain information on co-morbidities using a system based on the Charlson

For peer review only - http://bmjopen.bmj.com/site/about/guidelines.xhtml

Comorbidity Index[26] but excluding heart failure and stroke from the calculation since these were outcomes of interest in this study.

The primary outcome was first hospitalisation for any complication defined by the date of hospital admission when the relevant diagnostic code first appeared in the patient's records. The secondary outcomes were hospitalisation for each of the complications individually. We assumed no loss to follow-up because we expected the patient information system would include most hospitalisations in the country during 2008–2012.[24,25]

3.3 Statistical methods

We used survival methods to calculate cumulative incidence rates for each outcome in the cohort. Notably, the available coded discharge diagnoses data did not reliably distinguish between new and past diagnoses. Accordingly, to minimise the risk of counting events that occurred prior to the study, we limited the incidence rate calculations for the three more frequent outcomes, any complication, heart failure and atrial fibrillation, to the fourth and fifth year of the study. However, for the two remaining outcomes, ischaemic stroke and infective endocarditis, we used all the data available because: 1) these less frequent outcomes occurred at a comparatively constant rate throughout the study (Supplementary Figure S2); and 2) preliminary estimates based on the fourth and fifth year of the study tended to be imprecise. Having excluded nine individuals with missing sex information, we then used competing-risks regression (Stata *stcrreg* command)[27] to examine the impact of age (in three categories), sex and ethnicity (both as recorded in the hospital patient information system), and comorbidity on the risk of complications with death as the competing failure event.

To obtain rates of RHD-attributable morbidity in the general population, we used relative survival methods by applying background rates of hospitalisation to the cohort by sex, ethnicity and age in thirteen five-year age categories.[28] Background rates for each diagnosis were estimated by Poisson regression using the counts of hospitalisations from the patient information system and concurrent

For peer review only - http://bmjopen.bmj.com/site/about/guidelines.xhtml

denominator population estimates published by the Fiji Bureau of Statistics.[22] Specifically, we calculated age-specific rates for each complication using the observed minus the expected number (i.e. the excess) as the numerator divided by the population estimates as the denominator with 95% confidence intervals (CI) calculated using Poisson.

Years lived with disability were calculated by applying disability weights used by the GBD study to the person-time of the cohort during follow-up using weights: heart failure (severe), 0.179; stroke (moderate), 0.316; atrial fibrillation (symptomatic), 0.224; infective endocarditis (severe), 0.133; uncomplicated RHD, 0.049.[12] Where a patient had experienced more than one complication, disability weights were combined using a multiplicative model, as previously described.[29] To estimate disability-adjusted life-years (DALYs), we updated our previous estimates of years of life lost (YLLs)[14] using the GBD 2019 theoretical minimum risk life table.[12] In addition to summary estimates for the population of Fiji aged 0–69 years, we also calculated age-standardised rates using the GBD world population age standard[30] with rates of death and disability from our study for the population aged 65–69 years applied to the population aged ≥70 years.

To assess the relationship between RHD-attributable morbidity and mortality in the cohort, we focussed on the hospital cohort subset, defined by at least one attendance during follow-up at one of the two regional hospitals or the one national referral hospitals in the country. Alongside the opportunity for comparison with previously reported hospital-based studies, this subgroup had the advantage of a well-defined starting point for the time-to-event analysis (i.e. the earliest recorded hospital or outpatient clinic attendance in the study period at which a diagnosis of RHD was recorded) as well as the availability of additional clinical parameters for analysis (e.g. disease severity and presence of mitral stenosis). For each complication, having excluded five individuals with missing sex information, we split the follow-up time at the earliest point at which the complication occurred (Stata *stsplit* command) and plotted Kaplan-Meier estimates of survival based on the resulting variable to illustrate risk before and after the event occurred. We then used a Cox regression model to measure the relative impact of each of the complications, using the Schoenfeld residuals to test the

For peer review only - http://bmjopen.bmj.com/site/about/guidelines.xhtml

proportional-hazards assumption (Stata *phtest* command). All models included potential confounders – age, sex, ethnicity, comorbidity and rural residence – and evaluated interactions using likelihood ratio tests. Statistical analyses were conducted using Stata, version 12 (StataCorp, College Station, TX).

3.3 Patient and public involvement

Patients and/or the public were not involved in the design, or conduct, or reporting, or dissemination plans of this research.

4 Results

From our previously reported cohort of 2,619 patients,[14] we excluded 92 individuals known only for acute rheumatic fever, 302 identified only through echocardiographic screening, and 109 for whom the only available information was a death certificate (Supplementary Figure S1). The cohort comprised 2,116 patients with clinically apparent RHD followed for a median of 5.0 years. Most were aged 5-39 years (74.6%) and more than half were female (57.7%, Table 1).

4.1 RHD-attributable morbidity in the cohort

There were 2,116 patients in the national cohort, totalling 9,439 years of person-time. During follow-up, 1,335 (63.1%) patients were hospitalised a total of 2,896 times, equating to 1.4% of the total 205,427 admissions for any cause in the population of Fiji aged 5–69 years during 2008–2012. The most frequent discharge diagnostic codes were heart failure (22.5% of admissions) and atrial fibrillation (17.6%) (Supplementary Table S2).

For peer review only - http://bmjopen.bmj.com/site/about/guidelines.xhtml

In total, 546 patients (25.8%) were admitted with a discharge diagnosis consistent with a complication of RHD (Figure 1). More specifically, 349 patients (16.5%) were admitted with heart failure, 268 (12.7%) for atrial fibrillation, 104 (4.9%) for ischaemic stroke and 73 (3.4%) for endocarditis (Supplementary Table S3). The yearly cumulative incidence rate of any complication was 4.6% per year (95% CI 3.9–5.4) with rates of all complications except infective endocarditis increasing with age (Table 2 & Supplementary Table S4).

Table 1: **Baseline characteristics of patients by sex in the national, hospital and maternity cohorts.** *

	**National** (n = 2,116)		**Hospital** (n = 1,300)		**Maternity** (n = 210)
	Male (n = 892)	Female (n = 1,215)	Male (n = 547)	Female (n = 748)	Female (n = 210)
Age, n (%)					
5–14 years	356 (39.9)	326 (26.8)	197 (36.0)	179 (23.9)	5 (0.95)
15–39 years	312 (35.0)	577 (47.5)	191 (34.9)	356 (47.6)	202 (96.2)
40–69 years	224 (25.1)	312 (25.7)	159 (29.1)	213 (28.5)	6 (2.9)
Ethnicity, n (%)					
iTaukei (Indigenous)	527 (59.1)	775 (63.8)	332 (60.7)	500 (66.8)	151 (72.3)
Fijian of Indian Descent	294 (33.0)	366 (30.1)	166 (30.4)	201 (26.9)	41 (19.6)
Other	71 (8.0)	74 (6.1)	49 (9.0)	47 (6.3)	17 (8.1)
Data sources, n (%)					
Hospital admission	375 (42.0)	562 (46.3)	324 (59.2)	461 (61.6)	135 (64.3)
Echocardiography clinic	427 (47.9)	608 (50.0)	341 (62.3)	480 (64.2)	129 (61.4)
Control programme	523 (58.6)	682 (56.1)	185 (33.8)	223 (29.8)	92 (43.8)
Comorbidity†, n (%)					
Index = 0	763 (85.5)	1,047 (86.2)	435 (79.5)	615 (82.2)	195 (92.9)
Index = 1	48 (5.4)	46 (3.8)	41 (7.5)	33 (4.4)	7 (3.3)
Index ≥2	81 (9.1)	122 (10.0)	71 (13.0)	100 (13.4)	8 (3.8)
Rural residence, n (%)	400 (44.8)	515 (42.4)	245 (44.8)	301 (40.2)	64 (30.5)
Pregnancy, n (%)	0	210 (17.3)	0	155 (20.7)	210 (100)

* Sex information missing for nine patients in the national cohort and five patients in the hospital cohort. †Modified Charlson Comorbidity[26] calculated without points awarded for heart failure and stroke.

For peer review only - http://bmjopen.bmj.com/site/about/guidelines.xhtml

Table 2: **Cumulative incidence rates per 100 person-years with 95% confidence intervals of nonfatal complications of RHD within the national cohort [*].**

Age	Sex	Any complication [†]	Heart failure	Atrial fibrillation	Ischaemic stroke	Infective endocarditis
5-14 yrs	Male	1.7 (0.8–3.6)	1.2 (0.5–2.9)	0	0.2 (0.0–0.7)	0.9 (0.5–1.6)
	Female	1.1 (0.4–2.9)	0.8 (0.3–2.5)	0	0.2 (0.0–0.7)	0.5 (0.2–1.2)
15-39 yrs	Male	4.9 (3.5–7.0)	2.7 (1.7–4.2)	2.1 (1.2–3.5)	1.0 (0.6–1.6)	1.4 (0.9–2.1)
	Female	2.8 (2.0–4.0)	1.5 (0.9–2.4)	1.5 (0.9–2.4)	0.7 (0.4–1.0)	0.5 (0.3–0.9)
40-69 yrs	Male	12.8 (9.9–18.3)	6.5 (4.2–10.3)	6.6 (4.3–10.2)	2.9 (2.0–4.2)	1.0 (0.5–1.9)
	Female	10.3 (7.5–14.2)	4.8 (3.2–7.4)	8.5 (6.2–11.8)	2.6 (1.8–3.6)	0.4 (0.2–0.9)

[*] Rates are based on hospitalisation during the fourth or fifth year for any complications, heart failure and atrial fibrilation but otherwise at any stage during the study. Rates for the entire study period are given in Supplementary Table S4. [†] At least one hospitalisation for heart failure, stroke, atrial fibrillation or endocarditis.

For peer review only - http://bmjopen.bmj.com/site/about/guidelines.xhtml

For peer review only

In a multivariate analysis with adjustment for age and comorbidity, complications occurred more frequently in men than in women (hazard ratio, HR, 1.6, 95% CI 1.2–2.3, P=0.005, Supplementary Table S5). In addition, Indigenous iTaukei or other ethnicity was associated with a more than two-fold increased risk of complications compared to Fijians of Indian Descent (HR, 2.7, 95% CI 1.8–4.0, P<0.001). Across the complications, residential location (urban/rural) had no impact on risk (P>0.20).

4.2 RHD-attributable morbidity in the general population

To estimate RHD-attributable morbidity in the general population, we compared rates of hospitalisation for complications to those in the general population. Among the population 5-69 years in Fiji during 2008–2012, 651 of 4756 (13.7%) hospitalisations with a discharge diagnosis consistent with heart failure, 509 of 2225 (22.9%) with atrial fibrillation, 127 of 1927 (6.6%) with ischaemic stroke and 73 of 108 (67.6%) with endocarditis occurred in patients with an underlying diagnosis of RHD (Figure 2). However, before 40 years of age, 210 of 454 (46.2%) hospitalisations with heart failure, 143 of 288 (49.7%) with atrial fibrillation and 31 of 134 (23.3%) for stroke were associated with an underlying diagnosis of RHD.

We then calculated rates of RHD-attributable morbidity in the general population (Figure 2). For the Fiji population aged 0–69 years, we estimated that for every 100,000 person-years there were 14.7 (95% CI 13.6–16.0) excess hospitalisations for heart failure, 11.4 for atrial fibrillation (95% CI 10.4–12.5), 2.2 for ischaemic stroke (95% CI 1.8–2.7) and 1.7 for infective endocarditis (95% CI 1.3–2.1) each year (Supplementary Table S6). While absolute numbers of RHD-attributable complications peaked in the third decade, the incidence of all but infective endocarditis increased with age. In a multivariate analysis, incidence rates of complications were lower outside the Fijian of Indian Descent population (Supplementary Table S7), with this difference being most marked for stroke (incidence rate ratio, IRR, 1.8, 95% CI 1.1–2.8, P=0.013) and infective endocarditis (IRR 2.1, 95% CI 1.2–3.8, P=0.012). However, contrary to the within cohort risk calculations, overall incidence of complications

For peer review only - http://bmjopen.bmj.com/site/about/guidelines.xhtml

was higher in women than in men (IRR 1.4, 95% CI 1.3-1.6, P<0.001), an effect that appeared driven predominantly by higher rates of atrial fibrillation (IRR 1.5, 95% CI 1.3-1.8, P<0.001).

We estimated a yearly rate of 18.2 RHD-attributable YLDs (95% CI 16.9–19.5) per 100,000 person-years in the Fiji population aged 0–69 years (Supplementary Table S8). Rates of RHD-attributable disability increased during adolescence and thereafter increased only marginally (Supplementary Figure S3). Heart failure alone accounted for 18.7% of YLDs with 28.6% due to other complications while the remaining 52.7% were attributable to RHD without complications. Combined with our previous analyses of RHD-attributable mortality for the same population[14], we calculated 467.1 DALYs (95% CI 460.5–473.7) per 100,000 person-years were lost to RHD in the Fiji population aged 0–69 years during the study period.

4.3 Relationship between RHD-attributable morbidity and mortality

There were 1300 patients in the hospital cohort with at least one admission to one of the two regional or one national referral hospitals or outpatient attendance at one of the national referral hospital during follow-up (Table 1). This cohort accounted for 3,118 person-years with a median follow-up time of 2.4 years and 238 deaths (18.5%), equating to an overall yearly death rate of 7.2% (95% CI 6.3–8.2).

Median survival time was markedly shortened after admission with a complication to 3.6 years after any complication, 2.1 years after heart failure and 2.4 years after stroke (Figure 3). Adjusted for age, sex, ethnicity, comorbidity and rural residence, admission for any complication was associated with a five-fold increased risk of death for the remainder of follow-up (HR 5.4, 95% CI 3.4–8.8, P<0.001). Moreover, admission for heart failure, atrial fibrillation, stroke and infective endocarditis all had independent effects on risk of death, although the independent effect of atrial fibrillation was only apparent before the onset of heart failure (Supplementary Table S9). However, while heart failure and atrial fibrillation had a lasting effect on risk of death, that associated with stroke and infective endocarditis was short-lived and only apparent within the first 28 days of admission.

For peer review only - http://bmjopen.bmj.com/site/about/guidelines.xhtml

4.4 RHD-attributable morbidity and mortality during maternity

There were 210 women in the maternity cohort with at least one diagnostic code indicating pregnancy or childbirth during follow-up (17.3% of 1,215 women in the entire cohort), 204 women with a single pregnancy and six women with two pregnancies. A total of 164 deliveries (75.9%) were managed at the national referral hospital, 46 deliveries (21.3%) at two regional referral hospitals and the remaining six at subdivisional or unknown locations. The median age at delivery was 27.0 years (IQR 22.8–31.9).

A total of 123 deliveries (56.9%) had diagnostic codes indicating a complication of labour or delivery of which the most frequent were perineal laceration (74 deliveries), fetal distress (23 deliveries) and umbilical cord complications (10 deliveries). The mode of delivery was indicated in only 50 records (six indicating caesarean section, three forceps or vacuum extraction and the remainder spontaneous). Ten of 210 women (4.8%) had a diagnostic code suggesting a prior or co-existent complication of RHD including three each with heart failure, ischaemic stroke and infective endocarditis and two with atrial fibrillation (none of these women died during follow-up). However, overall, 11 of 210 women (5.2%) died during follow-up at a median age 32.2 years, including five after the onset of at least one RHD complication. While none of these deaths occurred within 42 days of delivery, three occurred within one year of delivery (i.e. late maternal deaths) of which one death was attributable to RHD and two to other causes.

5 Discussion

In this nationwide, population-based study, we provide evidence that RHD underlies a substantial proportion of cardiovascular morbidity among children and young adults in Fiji, likely reflecting the situation in LMICs worldwide.

For peer review only

For peer review only - http://bmjopen.bmj.com/site/about/guidelines.xhtml

Our analyses provide a considerable amount of new information about the epidemiology of nonfatal RHD complications in LMICs. First, we observed a high burden of RHD-attributable disability among adolescents and young adults, especially women of childbearing age, with women in the general population at greater risk than men overall. Second, we found a large burden of disability in the population attributable not only to heart failure but also to other complications of RHD – together these other complications accounted for a greater number of YLDs than heart failure alone, which is striking given the reliance on the latter in global summary estimates.[7,9] Third, we found that all four of the nonfatal complications that we assessed were independently associated with an increased risk of death. Consequently, notwithstanding the substantial rates of nonfatal RHD complications in this setting, our study indicates that the dominant public health impact of RHD in this setting is premature death rather than chronic disability.

There are few previously published studies available for comparison to our population-based estimates. Nonetheless, comparison of estimates from our hospital cohort with those from previous hospital-based studies helps benchmark our findings. Focusing on the REMEDY study,[15] there is a clear resemblance between the rates of new events in our hospital cohort during the initial two years of follow-up and those reported by the REMEDY investigators (Supplementary Figure S4).[15] This is illustrated, for example, by the 14.3% of hospital cohort patients who died within two years compared to 12.5% of patients in REMEDY for upper middle-income countries. This comparability underscores the vital importance of our ascertainment of patients away from the hospital setting and increases the generalisability of our findings beyond the Pacific region.

An additional comparison is with the recently reported ERASE project from Australia, which also identified cases using record-linkage.[18] The most relevant comparison is the yearly cumulative incidence rate of heart failure within a year of diagnosis, which at 3.2% in the Australian study[18] is similar to the 2.5% estimated from our study with overlapping confidence intervals. Additionally, the rates of nonfatal complications that we report are similar to those from a hospital-based cohort reported from the high income Pacific nation of New Caledonia.[31] Beyond this, we and others have

For peer review only - http://bmjopen.bmj.com/site/about/guidelines.xhtml

previously highlighted the burden of RHD complications in hospital- and community-based cohorts in the Pacific and beyond.[17,19,32–34] While broadly consistent with our findings, none of these prior studies provided the population-based estimates of disease burden needed to better define the scale of the RHD problem in LMICs.

This analysis extends our previous investigation of cardiac complications of childbirth in Fiji at the national referral hospital.[35] However, the number of complications detected here are much lower than ascertained by case record review. For example, prior or coexistent heart failure was noted in only three women (2%) in the current study compared to 23% in the case note review.[35] While it plausible rates are lower in the general population compared to the national referral hospital, it is also likely these lower rates reflect variable recording of non-obstetric diagnostic codes in the hospital patient information system in this group. Accordingly, the rates of complications that we report here are low relative to previous studies[36–38] and should be interpreted with caution. Nonetheless, the substantial number of deaths in this group during the remainder of follow-up is a stark reminder of the need for new approaches to tackle the burden of RHD among women of child-bearing age.[39]

Finally, the GBD study provides the key comparison for our population-based estimates, including those specific to Fiji for the same period.[7,9,12] Our point estimate of YLDs at 18.2 per 100,000 in the population aged 0–69 years is 40% lower than that made by GBD, which, inferred from prevalence, pertains only to heart failure. Importantly, we found a markedly different age-distribution of YLDs to that suggested by GBD, with relatively fewer RHD-attributable YLDs in young adults, likely reflecting markedly shortened survival times after the onset of complications. Accordingly, 96% of DALYs in our study were the result of YLLs, reflecting RHD-attributable deaths among adolescents and young adults,[14] and our DALYs estimate at 467.1 per 100,000 in the population aged 0–69 years is 28% higher than that from GBD. Moreover, since this may reflect the situation in LMICs more widely, it is our view that these discrepancies mandate further work to measure RHD-attributable death and disability in other settings to inform future GBD estimates.

For peer review only - http://bmjopen.bmj.com/site/about/guidelines.xhtml

Although our results appear reasonable, they have some limitations. First, notwithstanding the value of linked routine data for epidemiological analyses, there is likely to be some inaccuracy in our dataset, including in the precision of coding of discharge diagnoses. Nonetheless, since there is little reason to suspect diagnostic codes would have been used differently in patients with and without RHD, we expect this limitation to have limited impact on our population-based estimates of RHD-attributable morbidity. Future studies might increase accuracy by using algorithms developed to improve ascertainment of RHD cases from routine data, although these algorithms have yet to be validated in data from LMICs.[40]

Second, our data did not distinguish new from pre-existing complications and complications were frequently present at or shortly after diagnosis. To counter this, we limited estimates of the incidence of heart failure (as well as 'any complication' and atrial fibrillation) to the fourth and fifth years of the study on the presumption that almost all of those who developed these events prior to this period would either have either died or been detected during the first three years of follow-up. This 'clearance' period is shorter than the eight years in a recent Australian study[18] but supported by event rates in our cohort (Supplementary Figure S2). In addition, to calculate population-based rates, we made comparisons with hospitalisation data from the general population from the same period, which we expect to comprise a similar make up of new and pre-existing complications.

Third, our data lack important information of clinical relevance, including recurrence of acute rheumatic fever, detailed echocardiographic correlates of risk, and the impact of therapeutics such as penicillin prophylaxis and anticoagulation. Finally, reflecting external factors, we recognise that several years have now passed since the period from which our multiple sources of routine data were available. Nonetheless, we maintain they provide invaluable insight into the burden of morbidity and mortality attributable to RHD in low resource settings, not least because it is likely to be a number of years before interventions targeting reduction of childhood *S. pyogenes* infections and improved provision of penicillin prophylaxis impact the burden of disease in the adult population in Fiji.

For peer review only - http://bmjopen.bmj.com/site/about/guidelines.xhtml

5.1 Conclusions

By summarising burden of nonfatal complications of RHD in Fiji, our study underscores the assertion that effective control of RHD in LMICs could have a substantial impact on the global burden of cardiovascular morbidity and mortality in the young. However, without significant investment in efforts to improve knowledge of RHD epidemiology in LMICs worldwide, targeting and evaluating the impact of control measures will remain problematic, including, ultimately, delivery of vaccines against the causative *S. pyogenes*.

For peer review only

For peer review only - http://bmjopen.bmj.com/site/about/guidelines.xhtml

6 Data availability

The authors did not generate the primary dataset which is the property of the Fiji Ministry of Health and Medical Services. Researchers wishing to access Fiji Ministry of Health data should send a request in writing to the Permanent Secretary for Health, Fiji Ministry of Health and Medical Services, Dinem House, 88 Amy Street, PO Box 2223, Government Buildings, Suva, Fiji Islands (see also: http://www. health.gov.fj). Use of Fiji Ministry of Health data would also be subject to approval by the Fiji National Health Research Committee. The study protocol is available for download at: http://users.ox.ac.uk/~clme1250/rhd_epi/. An earlier version of this manuscript was released as a preprint at: http://dx.doi.org/10.2139/ssrn.4237745.

7 Ethical approval

The study was approved by the Fiji National Research Ethics Review Committee (2013-89) and the Oxford University Tropical Research Ethics Committee (1055-13).

8 Contributions

TP, JK, MLP, JF, SMC, ACS conceived the study. TP, JK, LN, SMC, ACS acquired and analysed the underlying data. TP, JK, LN, MLP, KS, JF, DE, SMC, ACS interpreted the findings. TP wrote the first draft of the manuscript. All authors contributed to and critically revised the manuscript. All authors approved the final version.

9 Acknowledgements

We thank the Ministry of Health and Medical Services and the National Disease Control Programme in Fiji for providing the datasets as well as their employees who compiled them, particularly those at the Ministry of Health Information Unit. We thank and recognise the contribution of the late Dr Isimeli Tukana, formerly Head of Wellness and National Adviser for Non-Communicable Disease at the Fiji Ministry of Health and Medical

For peer review only

Services, for his vital contribution to conception and design of the study. We also thank Dr Anne E Hannay (née Miller), Mr Brenton Ward, Dr Rachel Heenan, Ms Tuliana Cua and Ms Maureen Ah Kee for their contribution to the data collection.

10 Funding

This work was supported by the Medical Research Council UK (G1100449 to TP), the British Medical Association Foundation for Medical Research (Research Grant 2012 to TP), the Sir Halley Stewart Trust (Research Grant 2013 to TP), the National Institute for Health and Care Research UK (CL-2020-21-001 to TP) and the Wellcome Trust (222098/Z/20/Z to TP). These funders had no role in study design, data collection and analysis, decision to publish, or preparation of the manuscript.

11 Competing interests

None declared.

For peer review only

12 References

1. Remenyi B, ElGuindy A, Smith SC, Yacoub M, and Holmes DR. Valvular aspects of rheumatic heart disease. The Lancet 2016;387:1335 –1346. doi: 10.1016/s01406736(16)00547-x.

2. Carapetis JR, McDonald M, and Wilson NJ. Acute rheumatic fever. The Lancet 2005;366:155 –168. doi: 10.1016/s0140-6736(05)66874-2.

3. Marijon E, Mirabel M, Celermajer DS, and Jouven X. Rheumatic heart disease. The Lancet 2012;379:953 –964. doi: 10.1016/s0140-6736(11)61171-9.

4. Beaton A, Okello E, Rwebembera J, et al. Secondary Antibiotic Prophylaxis for Latent Rheumatic Heart Disease. New England Journal of Medicine 2021;386:230– 40. doi: 10.1056/nejmoa2102074.

5. Engelman D, Mataika RL, Kado JH, et al. Adherence to secondary antibiotic prophylaxis for patients with rheumatic heart disease diagnosed through screening in Fiji. Tropical Medicine & International Health 2016;21:1583–91. doi: 10.1111/tmi. 12796.

6. Vekemans J, Gouvea-Reis F, Kim JH, et al. The Path to Group A Streptococcus Vaccines: World Health Organization Research and Development Technology Roadmap and Preferred Product Characteristics. Clinical Infectious Diseases 2019;69:877–83. doi: 10.1093/cid/ciy1143.

7. Watkins DA, Johnson CO, Colquhoun SM, et al. Global, Regional, and National Burden of Rheumatic Heart Disease, 1990-2015. The New England Journal of Medicine 2017;377:713 –722. doi: 10.1056/nejmoa1603693.

8. Sliwa K, White A, Milan P, Mocumbi AO, Zilla P, and Wood D. Momentum builds for a global response to rheumatic heart disease. European Heart Journal 2018;39:4229 –4232. doi: 10.1093/eurheartj/ehy763.

9. Roth GA, Mensah GA, Johnson CO, et al. Global Burden of Cardiovascular Diseases and Risk Factors, 1990–2019 Update From the GBD 2019 Study. Journal of the American College of Cardiology 2020;76:2982–3021. doi: 10.1016/j.jacc.2020. 11.010.

10. Ghamari S, Abbasi-Kangevari M, Moghaddam SS, et al. Rheumatic Heart Disease Is a Neglected Disease Relative to Its Burden Worldwide: Findings From Global Burden of Disease 2019. Journal of the American Heart Association 2022;11:e025284. doi: 10.1161/jaha.122.025284.

11. White A. WHO Resolution on rheumatic heart disease. European Heart Journal 2018;39:4233 – 4233. doi: 10.1093/eurheartj/ehy764.

12. GBD 2019 Diseases and Injuries Collaborators, Vos T, Lim SS, et al. Global burden of 369 diseases and injuries in 204 countries and territories, 1990–2019: a systematic analysis for the Global Burden of Disease Study 2019. The Lancet 2020;396:1204– 22. doi: 10.1016/s0140-6736(20)30925-9.

13. Abouzeid M, Wyber R, Vincente SL, et al. Time to tackle rheumatic heart disease: Data needed to drive global policy dialogues. Global public health 2018;14:1 –13. doi: 10.1080/17441692.2018.1515970.

14. Parks T, Kado J, Miller AE, et al. Rheumatic Heart Disease-Attributable Mortality at Ages 5-69 Years in Fiji: A Five-Year, National, Population-Based Record-Linkage Cohort Study. PLoS Neglected Tropical Diseases 2015;9:e0004033. doi: 10.1371/ journal.pntd.0004033.

15. Zühlke L, Karthikeyan G, Engel ME, et al. Clinical Outcomes in 3343 Children and Adults With Rheumatic Heart Disease From 14 Low- and Middle-Income Countries. Circulation 2016;134:1456–66. doi: 10.1161/circulationaha.116.024769.

For peer review only - http://bmjopen.bmj.com/site/about/guidelines.xhtml

16. Noubiap JJ, Agbor VN, Bigna JJ, Kaze AD, Nyaga UF, and Mayosi BM. Prevalence and progression of rheumatic heart disease: a global systematic review and metaanalysis of population-based echocardiographic studies. Scientific Reports 2019;9:17022. doi: 10.1038/s41598-019-53540-4.

17. Jaiteh LES, Drammeh L, Anderson ST, et al. Rheumatic heart disease in The Gambia: clinical and valvular aspects at presentation and evolution under penicillin prophylaxis. BMC Cardiovascular Disorders 2021;21:503. doi: 10.1186/s12872021-02308-8.

18. Stacey I, Hung J, Cannon J, et al. Long-term outcomes following rheumatic heart disease diagnosis in Australia. European Heart Journal Open 2021;1:oeab035. doi: 10.1093/ehjopen/oeab035.

19. Zimmerman M, Kitooleko S, Okello E, et al. Clinical outcomes of children with rheumatic heart disease. Heart 2022;108:633–8. doi: 10.1136/heartjnl-2021320356.

20. Zühlke LJ and Steer AC. Estimates of the Global Burden of Rheumatic Heart

Disease. Global Heart 2013;8:189–95. doi: 10.1016/j.gheart.2013.08.008.

21. Ronsmans C, Graham WJ, and group oboTLMSSs. Maternal mortality: who, when, where, and why. The Lancet 2006;368:1189–200. doi: 10.1016/s0140-6736(06) 69380-x.

22. Fiji Bureau of Statistics. 2007 Census of the Population. Tech. rep. 2008. URL:

http://www.statsfiji.gov.fj.

23. Fiji Bureau of Statistics. Fiji Population & Housing Census 2017. Tech. rep. Fiji Bureau of Statistics, 2017. url: https://www.statsfiji.gov.fj/index.php/ census-2017.

24. Roberts G, Irava W, Tuiketei T, et al. The Fiji Islands Health System Review. Health Systems in Transition 2011;1. Ed. by Tulloch J. URL: https://apps.who.int/iris/bitstream/handle/10665/207503/9789290615439_eng.pdf.

25. Reyburn R, Nand D, Nguyen C, et al. Validation of administrative data to estimate vaccine impact: Audit of the Fiji hospital admissions electronic database, 2007–2011 & 2014–2015. Vaccine 2017;35:6416–21. doi: 10.1016/j.vaccine.2017.09.091.

26. Charlson ME, Pompei P, Ales KL, and MacKenzie CR. A new method of classifying prognostic comorbidity in longitudinal studies: development and validation. Journal of Chronic Diseases 1987;40:373 – 383.

27. Fine JP and Gray RJ. A Proportional Hazards Model for the Subdistribution of a Competing Risk. Journal of the American Statistical Association 1999;94:496. doi: 10.2307/2670170.

28. Dickman PW, Sloggett A, Hills M, and Hakulinen T. Regression models for relative survival. Statistics in Medicine 2004;23:51 –64. doi: 10.1002/sim.1597.

29. Murray CJL, Ezzati M, Flaxman AD, et al. GBD 2010: design, definitions, and metrics. The Lancet 2012;380:2063 –2066. doi: 10.1016/s0140-6736(12)61899-6.

30. GBD 2017 Causes of Death Collaborators, Roth GA, Abate D, et al. Global, regional, and national age-sex-specific mortality for 282 causes of death in 195 countries and territories, 1980–2017: a systematic analysis for the Global Burden of Disease Study 2017. The Lancet 2018;392:1736–88. doi: 10.1016/s0140-6736(18) 32203-7.

31. Mirabel M, Tafflet M, Noël B, et al. Newly diagnosed rheumatic heart disease among indigenous populations in the Pacific. Heart 2015-12;101:1901 –1906. doi: 10.1136/heartjnl-2015-308237.

For peer review only - http://bmjopen.bmj.com/site/about/guidelines.xhtml

32. Steer AC, Kado J, Jenney AWJ, et al. Acute rheumatic fever and rheumatic heart disease in Fiji: prospective surveillance, 2005–2007. Medical Journal of Australia 2009;190:133–5. doi: 10.5694/j.1326-5377.2009.tb02312.x.

33. Sriharibabu M, Himabindu Y, and Kabir Z. Rheumatic heart disease in rural south India: A clinico-observational study. Journal of Cardiovascular Disease Research 2013;4:25–9. doi: 10.1016/j.jcdr.2013.02.011.

34. Okello E, Longenecker CT, Beaton A, Kamya MR, and Lwabi P. Rheumatic heart disease in Uganda: predictors of morbidity and mortality one year after presentation. BMC Cardiovascular Disorders 2017;17:20. doi: 10.1186/s12872-016-0451-8.

35. Narube L, Fong J, Parks T, Ekeroma AJ, and Kubuabola I. Pregnancy outcomes in women with heart disease at the Colonial War Memorial Hospital, Suva, Fiji. Pacific Journal of Reproductive Health 2016;1. doi: 10.18313/pjrh.2016.910.

36. Hagen IMv, Thorne SA, Taha N, et al. Pregnancy Outcomes in Women With Rheumatic Mitral Valve Disease. Circulation 2018;137:806–16. doi: 10.1161/circulationaha.117.032561.

37. Beaton A, Okello E, Scheel A, et al. Impact of heart disease on maternal, fetal and neonatal outcomes in a low-resource setting. Heart 2019;105:755. doi: 10.1136/ heartjnl-2018-313810.

38. Baghel J, Keepanasseril A, Pillai AA, Mondal N, Jeganathan Y, and Kundra P. Prediction of adverse cardiac events in pregnant women with valvular rheumatic heart disease. Heart 2020;106:1400–6. doi: 10.1136/heartjnl-2020-316648.

39. Vaughan G, Dawson A, Peek M, et al. Rheumatic Heart Disease in Pregnancy: New Strategies for an Old Disease? Global Heart 2021;16:84. doi: 10.5334/gh.1079.

40. Bond-Smith D, Seth R, Klerk Nd, et al. Development and Evaluation of a Prediction Model for Ascertaining Rheumatic Heart Disease Status in Administrative Data. Clinical Epidemiology 2020;12:717–30. doi: 10.2147/clep.s241588.

For peer review only

For peer review only - http://bmjopen.bmj.com/site/about/guidelines.xhtml

Figure 1: **Hospitalisations for nonfatal complications in the cohort.** The proportion of national cohort hospitalised for each complication of RHD are shown on the left with number of reaching each outcome indicated at the end of each bar. Cumulative incidence rates of each complication are shown on the right with 95% confidence intervals (CI).

a) Proportion of the cohort b) Cumulative incidence

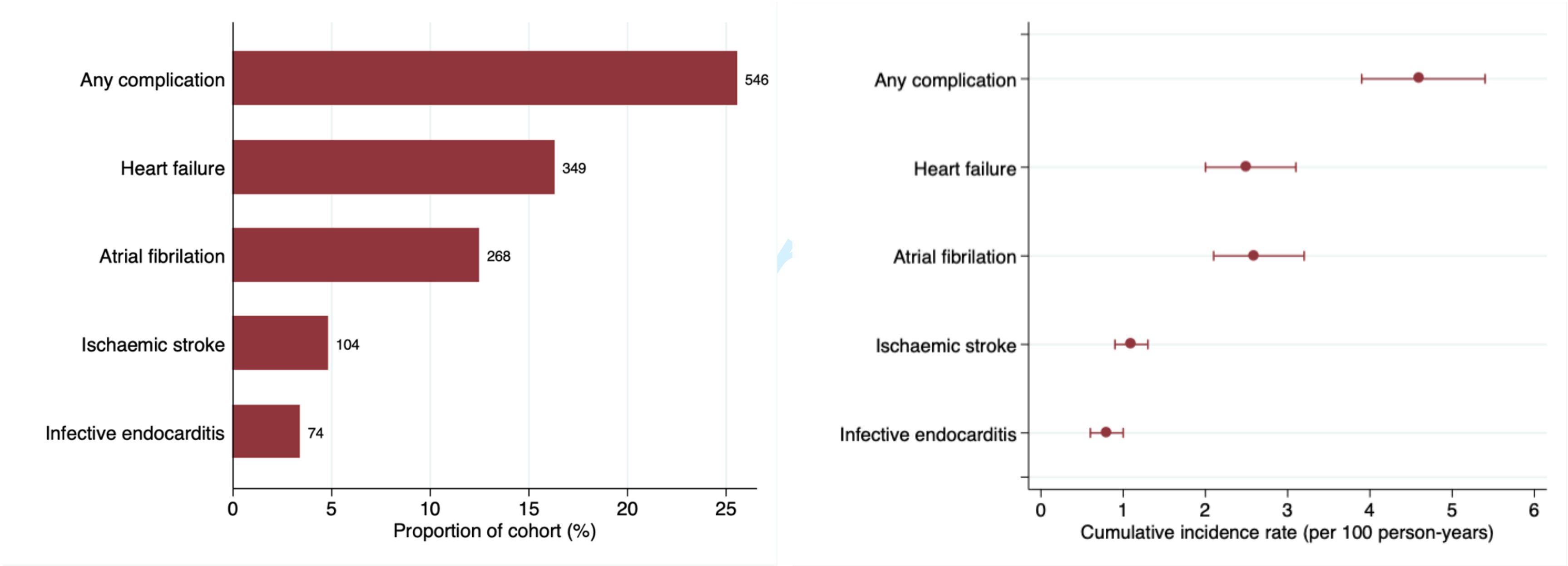

For peer review only - http://bmjopen.bmj.com/site/about/guidelines.xhtml

Figure 2: **Hospitalisation for heart failure, atrial fibrillation and stroke in the general population by age.** The number of hospitalisations for each complication are shown on the left with the fraction attributable to RHD coloured red. Population-based rates of RHD-attributable hospitalisation for each complication are shown on the right with 95% confidence intervals (CI) against the lefthand axis and the proportion attributable to RHD as a percentage against the righthand axis.

a) Heart failure (count)

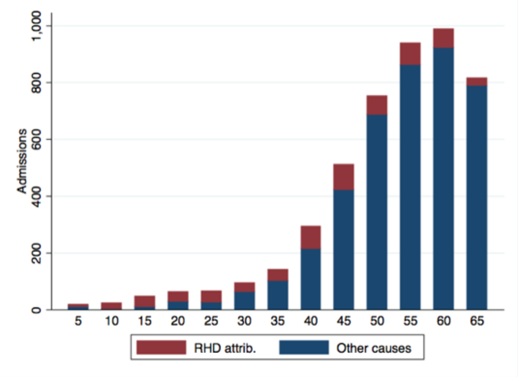

b) Heart failure (rate)

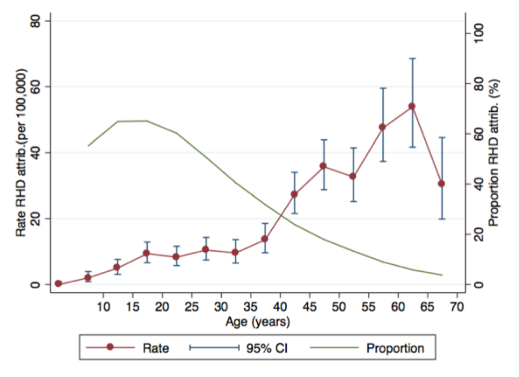

c) Atrial fibrillation (count)

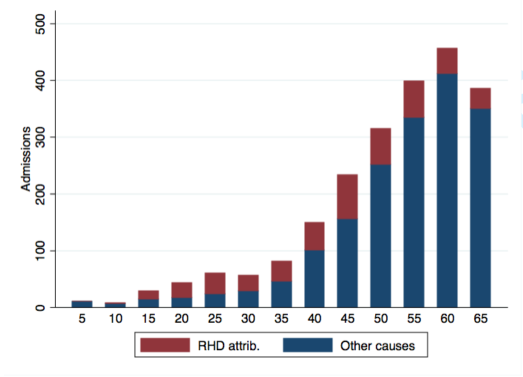

d) Atrial fibrillation (rate)

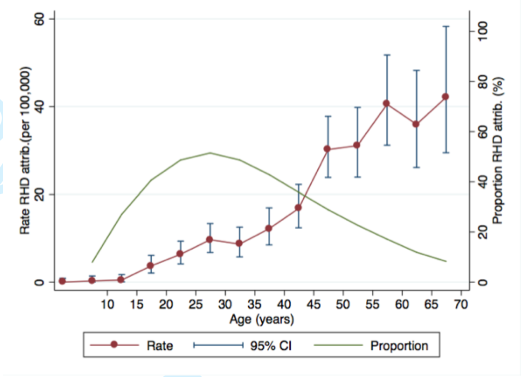

e) Ischaemic stroke (count)

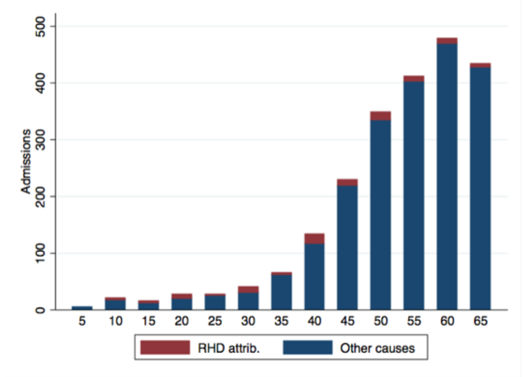

f) Ischaemic stroke (rate)]

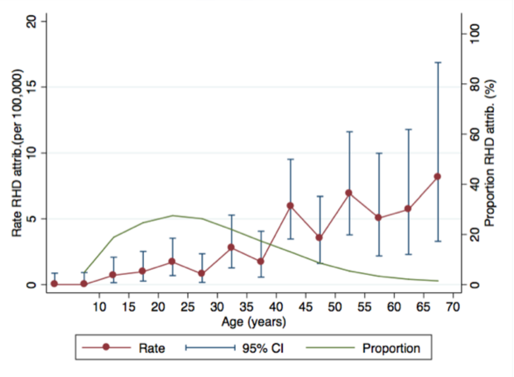

Figure 3: **Survival probability following hospitalisation for any complication, heart failure, atrial fibrillation and ischaemic stroke in the hospital cohort.** For each event, survival probability is plotted with 95% confidence intervals before and after hospitalisation with the number at risk at each timepoint in the table.

a) Any complication

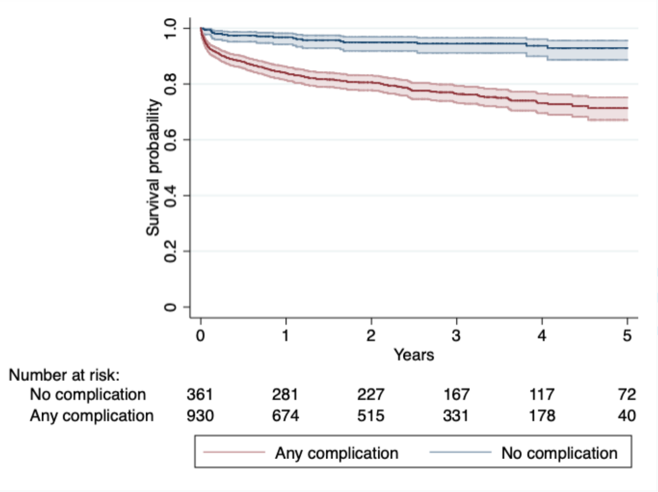

b) Heart failure

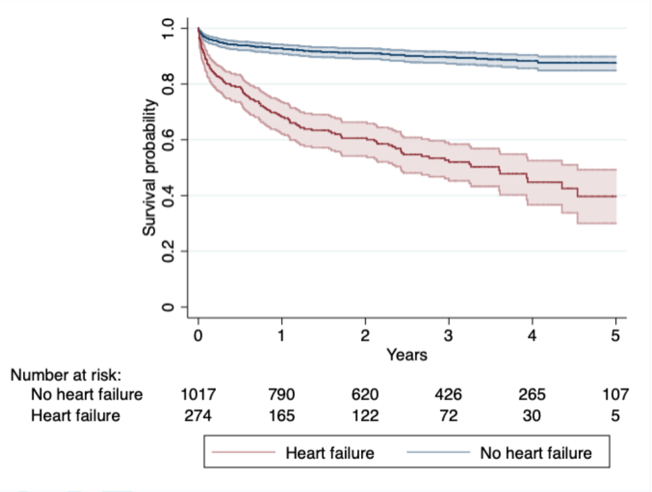

c) Atrial fibrillation

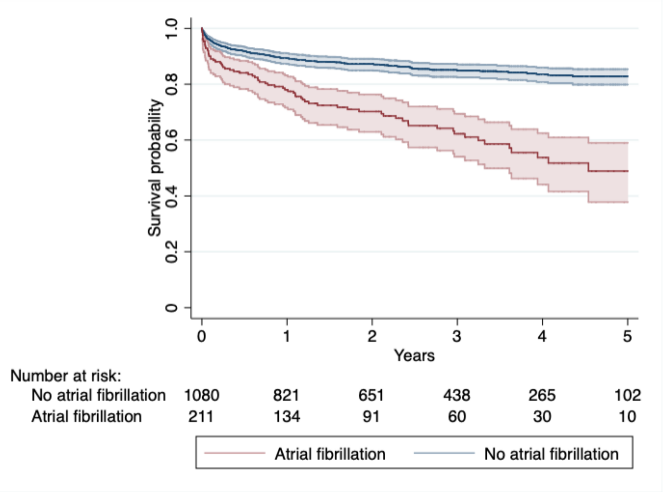

d) Ischaemic stroke

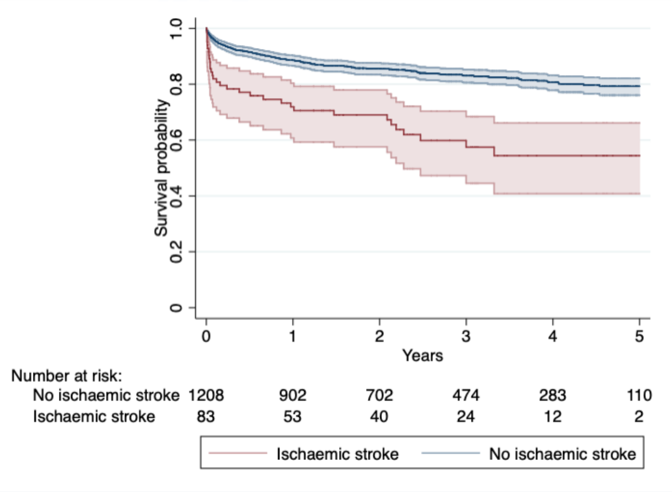

533 # Supplementary Material

For peer review only

For peer review only - http://bmjopen.bmj.com/site/about/guidelines.xhtml

Figure S1: **Overview of the study design.** Our primary analyses focused on the subset of patients from our previous reported study with clinically apparent RHD.[14] For further analyses, we defined two subsets: patients with at least one hospital attendance during follow-up, termed the hospital cohort, and women with at least one pregnancy during follow-up, termed the maternity cohort.

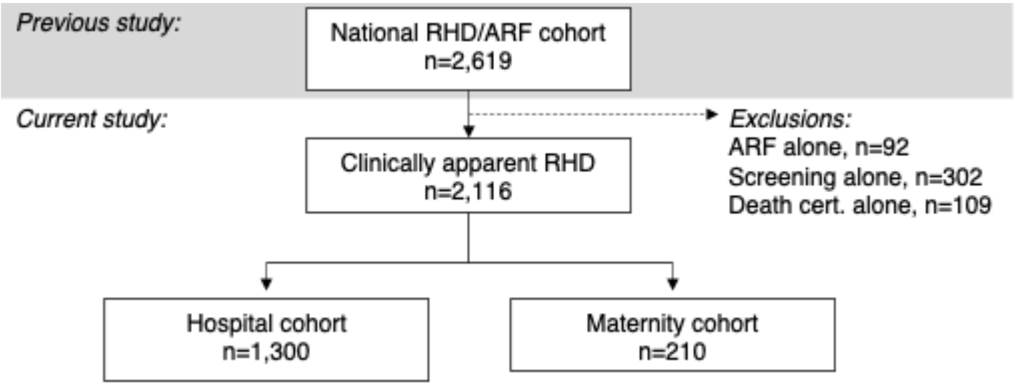

ARF, acute rheumatic fever.

For peer review only - http://bmjopen.bmj.com/site/about/guidelines.xhtml

Figure S2: **Cumulative incidence of any complication, heart failure, atrial fibrillation, ischaemic stroke and infective endocarditis in the national cohort.**

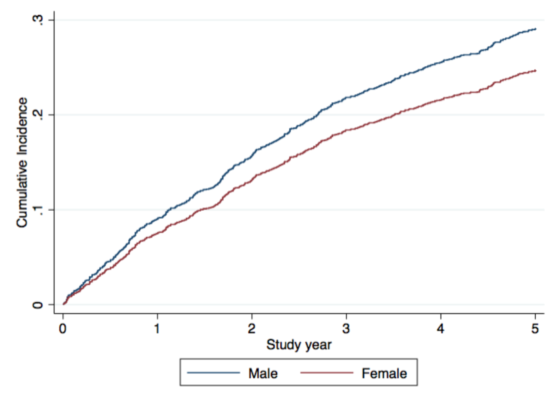

(a) All events

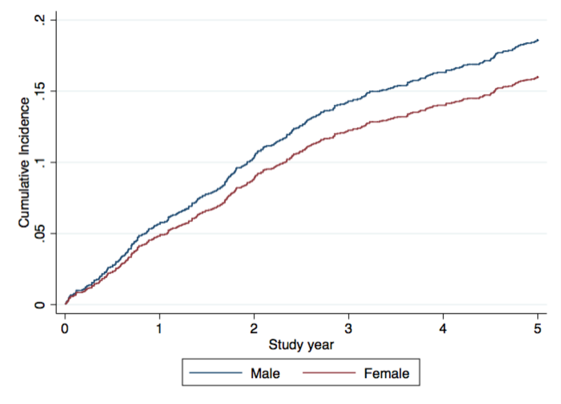

(b) Heart failure

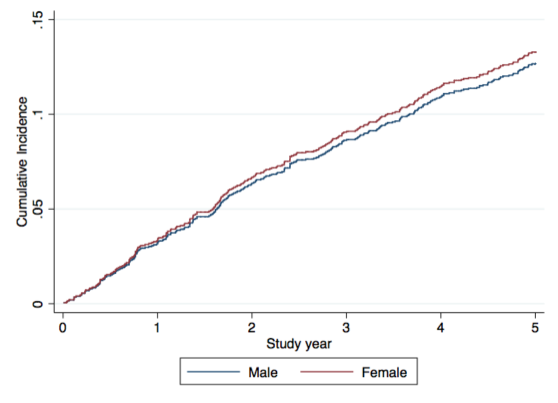

(c) Atrial fibrillation

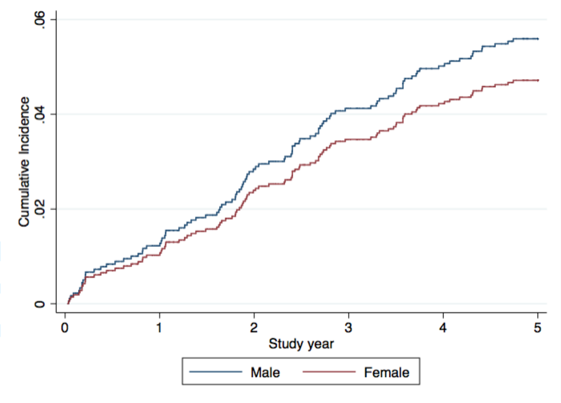

(d) Ischaemic stroke

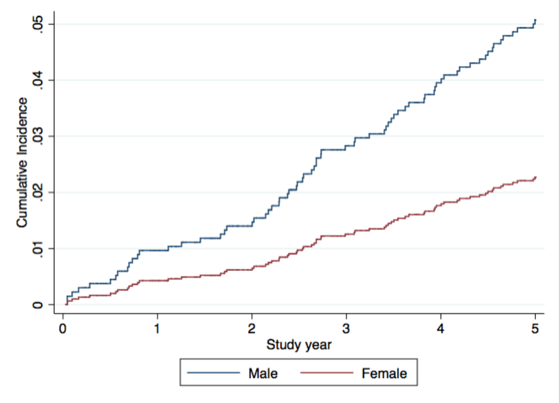

(e) Infective endocarditis

For peer review only - http://bmjopen.bmj.com/site/about/guidelines.xhtml

Figure S3: **Population-based rates of RHD-attributable disability from the study compared to estimates for the same period from the Global Burden of Disease (GBD) study.**[7,9,12]

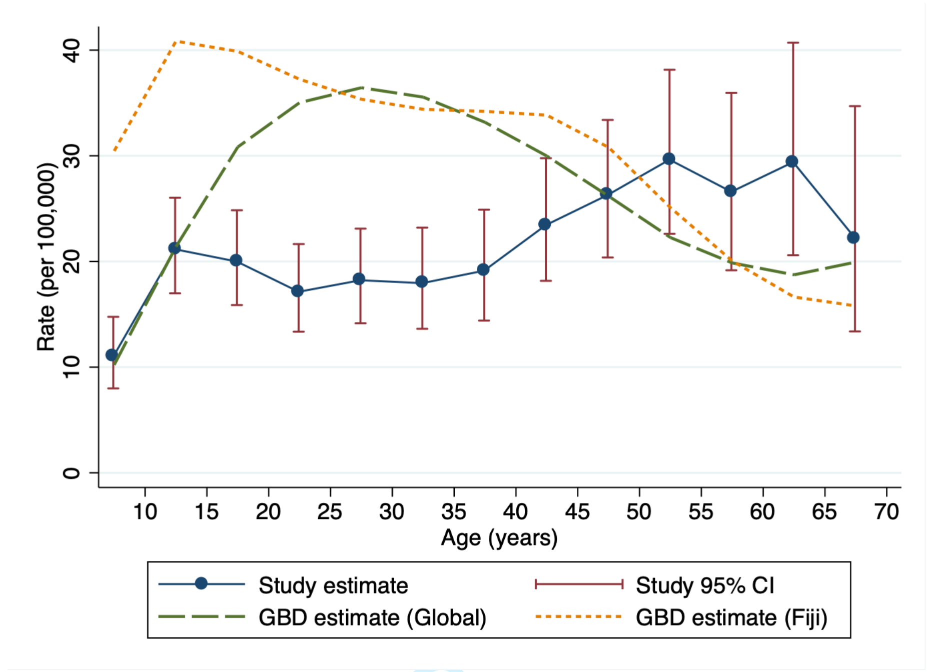

(a) Years lived with disability (YLDs)

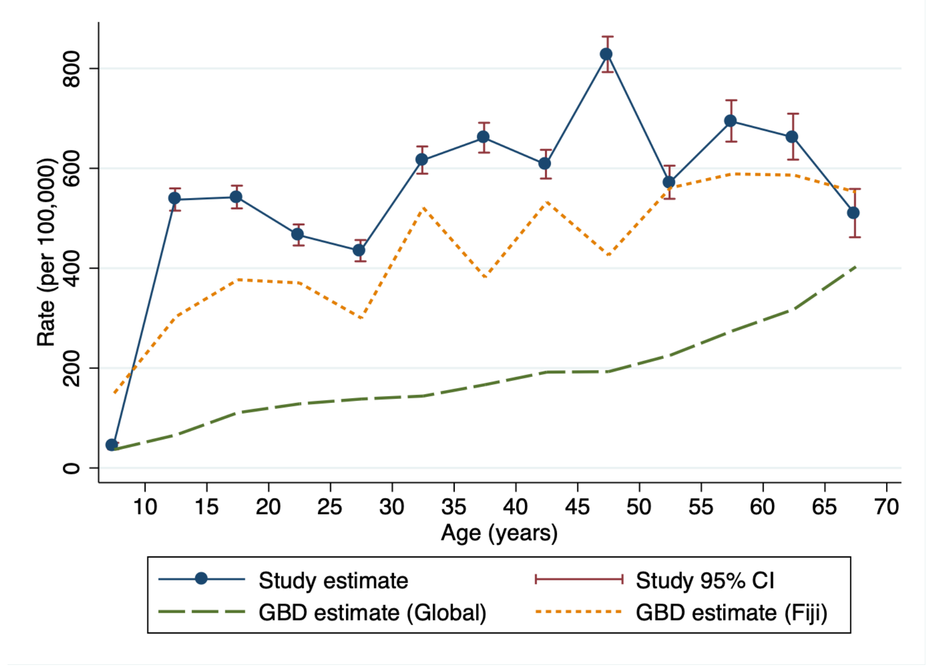

(b) Disability adjusted life-years (DALYs)

For peer review only - http://bmjopen.bmj.com/site/about/guidelines.xhtml

Figure S4: **Proportion of the hospital cohort with new RHD complications at 2 years follow-up compared to clinical outcomes at 2 years follow-up in the REMEDY cohort.**[15]

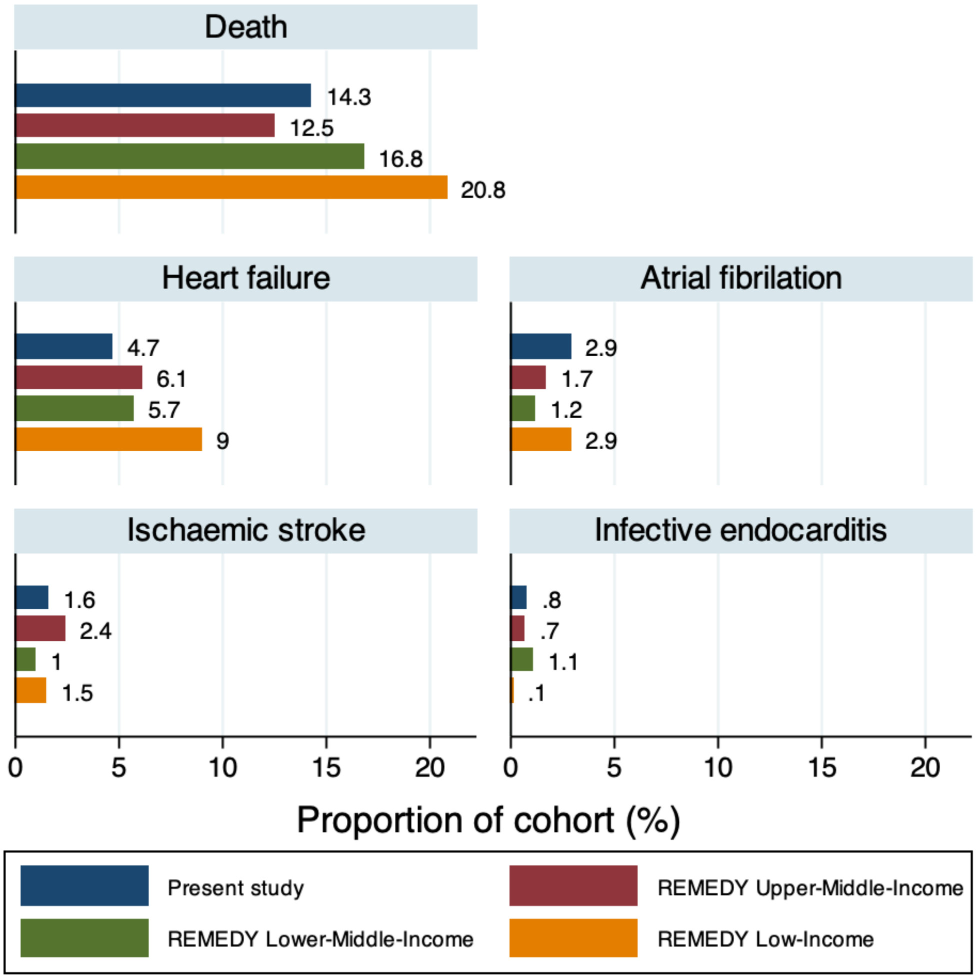

For peer review only - http://bmjopen.bmj.com/site/about/guidelines.xhtml

Table S1: **Diagnostic codes used for this analysis for rheumatic heart disease and its complications.**

**Diagnosis**	ICD-10 Codes*
Rheumatic heart disease	I05-I09
Acute rheumatic fever	I00-I02
Heart failure	I50
Atrial fibrillation	I48
Infective endocarditis	I33
Ischaemic stroke	G46, I63-64, I67.9, I69.3-I69.4, I69.8

*International Statistical Classification of Diseases and Related Health Problems 10th Revision (ICD-10) codes.

For peer review only - http://bmjopen.bmj.com/site/about/guidelines.xhtml

Table S2: **Numbers of individuals and admissions for which diagnostic codes were used in the cohort by age and sex*.** The percentage of individuals and admissions within each age category to which the code applies is also shown.

Diagnosis	Age (yrs)	Admissions, n (%)		Individuals, n (%)	
		Males n=1053	Females n=1830	Males n=892	Females n=1215
RHD	5-14	182 (60)	189 (66)	136 (38)	134 (42)
	15-39	186 (56)	437 (44)	128 (42)	273 (48)
	40-69	128 (30)	205 (38)	100 (44)	141 (46)
ARF	5-14	43 (14)	54 (18)	40 (12)	45 (14)
	15-39	7 (2)	17 (2)	7 (2)	16 (2)
	40-69	1 (0)	1 (0)	1 (0)	1 (0)
Heart failure	5-14	30 (10)	30 (10)	16 (4)	21 (6)
	15-39	90 (28)	93 (10)	53 (16)	66 (12)
	40-69	211 (50)	197 (36)	88 (40)	102 (32)
Atrial fibrillation	5-14	4 (2)	4 (2)	3 (0)	4 (2)
	15-39	79 (24)	74 (8)	46 (14)	51 (8)
	40-69	118 (28)	228 (42)	61 (28)	114 (36)
Ischaemic stroke	5-14	3 (2)	2 (0)	3 (0)	2 (0)
	15-39	16 (4)	24 (2)	15 (4)	20 (4)
	40-69	36 (8)	46 (8)	26 (12)	33 (10)
Endocarditis	5-14	18 (6)	8 (2)	16 (4)	8 (2)
	15-39	20 (6)	17 (2)	20 (6)	14 (2)
	40-69	8 (2)	5 (0)	8 (4)	5 (2)
Ischaemic heart diseas	5-14	6 (2)	2 (0)	6 (2)	2 (0)
	15-39	13 (4)	9 (0)	12 (4)	9 (0)
	40-69	142 (34)	88 (16)	70 (32)	59 (18)
Community acquired pneumonia	5-14	22 (8)	31 (10)	18 (6)	26 (8)
	15-39	29 (8)	43 (4)	26 (8)	35 (6)
	40-69	34 (8)	32 (6)	29 (12)	25 (8)
Diabetes mellitus	5-14	0 (0)	0 (0)	0 (0)	0 (0)
	15-39	2 (0)	6 (0)	1 (0)	4 (0)
	40-69	62 (14)	90 (16)	33 (14)	45 (14)
Obstetric	5-14	0 (0)	4 (2)	0 (0)	3 (0)
	15-39	0 (0)	447 (44)	0 (0)	215 (38)
	40-69	0 (0)	10 (2)	0 (0)	4 (2)

*Sex information missing for nine patients who had a total of 13 admissions.

For peer review only

Table S3: **Clinical events by sex in the national, hospital and maternity cohorts.***

	National (n = 2,116)		Hospital (n = 1,300)		Maternity (n = 210)
	Male (n = 892)	Female (n = 1,215)	Male (n = 547)	Female (n = 748)	Female (n = 210)
Hospital admission, n (%)					
Any complication	252 (28.3)	292 (24.0)	208 (38.0)	241 (32.2)	22 (10.5)
Heart failure	160 (17.9)	189 (15.6)	128 (23.4)	156 (20.9)	11 (5.2)
Atrial fibrillation	109 (12.6)	158 (13.0)	90 (16.5)	125 (16.7)	7 (3.3)
Ischaemic stroke	48 (5.4)	56 (4.6)	37 (6.8)	45 (6.3)	7 (3.3)
Infective endocarditis	45 (5.0)	27 (2.2)	40 (7.3)	23 (3.1)	4 (1.9)
Echocardiography, n (%)					
Moderate disease	103 (11.6)	162 (13.3)	81 (14.8)	137 (18.3)	45 (21.4)
Severe disease	112 (12.6)	147 (12.1)	100 (18.3)	122 (16.3)	29 (13.8)
Mitral stenosis	136 (15.3)	269 (22.1)	121 (22.1)	230 (30.8)	64 (30.5)
Heart valve surgery, n (%)					
Prior to study	32 (3.6)	54 (4.4)	26 (4.8)	38 (5.1)	7 (3.3)
During follow-up	51 (5.7)	75 (6.2)	39 (7.1)	56 (7.5)	10 (4.8)
Date uncertain	31 (3.5)	32 (2.6)	21 (3.8)	21 (2.8)	5 (2.4)
Death during follow-up, n (%)	147 (16.5)	173 (14.2)	113 (20.7)	127 (17.0)	11 (5.2)

*Sex information missing for nine patients in the national cohort and five patients in the hospital cohort.

For peer review only

Table S4: **Cumulative incidence rates per 100 person-years with 95% confidence intervals of nonfatal complications of RHD in the national cohort at various time intervals during the study.**

Age	Sex	Years	Any complication*	Heart failure	Atrial fibrillation	Ischaemic stroke	Infective endocarditis
5-14 years	Male	1-3	17.4 (14.5–21.0)	0.6 (0.3–1.5)	0.1 (0.0–0.9)	0.2 (0.1–1.0)	0.8 (0.3–1.7)
		4-5	8.4 (5.5–12.8)	1.2 (0.5–2.9)	0 (0.0-0.0)	0 (0.0-0.0)	1.2 (0.5–2.9)
		All	14.9 (12.5–17.7)	1.8 (0.4–1.5)	0.1 (0.0–0.6)	0.2 (0.0–0.7)	0.9 (0.5–1.6)
	Female	1-3	16.5 (13.5–20.0)	1.5 (0.8–2.7)	0.3 (0.1–1.1)	0.4 (0.1–1.3)	0.7 (0.3–1.6)
		4-5	11.4 (7.8–16.7)	0.8 (0.3–2.5)	0.3 (0.0–1.8)	0 (0.0-0.0)	0.3 (0.0–1.9)
		All	15.1 (12.7–17.9)	1.3 (0.8–2.1)	0.3 (0.1–0.8)	0.3 (0.1–0.8)	0.5 (0.2–1.2)
15-39 years	Male	1-3	13.2 (11.0–15.9)	3.6 (2.6–5.0)	3.1 (2.1–4.4)	1.7 (1.0–2.7)	0.9 (0.5–1.8)
		4-5	11.4 (8.7–14.9)	2.7 (1.7–4.2)	2.1 (1.2–3.5)	0.4 (0.1–1.3)	2.0 (1.2–3.4)
		All	12.6 (10.8–14.6)	3.2 (2.4–4.2)	2.7 (2.0–3.6)	1.1 (0.7–1.8)	1.4 (0.9–2.1)
	Female	1-3	17.1 (15.1–19.4)	3.0 (2.3–3.9)	1.6 (1.1–2.4)	0.8 (0.4–1.3)	0.7 (0.4–1.2)
		4-5	12.5 (10.1–15.5)	1.5 (0.9–2.4)	1.5 (0.9–2.4)	0.8 (0.4–1.5)	0.3 (0.1–0.8)
		All	15.6 (14.0–17.4)	2.4 (1.9–3.0)	1.6 (1.2–2.1)	0.8 (0.5–1.2)	0.5 (0.3–0.9)
40-69 years	Male	1-3	25.6 (21.4–30.5)	13.5 (10.7–16.8)	8.0 (6.0–10.6)	3.2 (2.1–5.0)	0.6 (0.2–1.7)
		4-5	20.3 (14.8–28.0)	6.5 (4.2–10.3)	6.0 (3.8–9.5)	3.0 (1.6–5.5)	1.7 (0.7–3.7)
		All	24.1 (20.6–28.1)	11.1 (9.1–13.6)	7.3 (5.7–9.3)	3.1 (2.2–4.5)	1.0 (0.5–1.9)
	Female	1-3	22.9 (19.6–26.8)	10.7 (8.7–13.3)	10.7 (8.6–13.2)	3.5 (2.5–5.0)	0.3 (0.1–1.1)
		4-5	17.2 (13.0–22.7)	4.8 (3.2–7.4)	8.6 (6.2–11.9)	2.4 (1.3–4.2)	0.6 (0.2–1.7)
		All	21.2 (18.5–24.3)	8.7 (7.2–10.5)	10.0 (8.3–11.9)	3.1 (2.3–4.2)	0.4 (0.2–0.9)

*At least one hospitalisation for heart failure, stroke, atrial fibrillation or infective endocarditis.

For peer review only

For peer review only

Table S5: **Hazard ratio estimates with 95% confidence intervals for new nonfatal complications of RHD within the national cohort in a multivariate competing-risks analysis***.

		Any complication‡	Heart failure	Atrial fibrillation	Ischaemic stroke	Infective endocarditis
Age (yrs)	5-14	1	1	1	1	1
	15-39	**2.77 (1.44–5.32)**	1.98 (0.91–4.30)	**20.4 (5.0–83.4)**	**4.19 (1.48–11.82)**	1.20 (0.67–2.14)
	40-69	**6.78 (3.46–13.28)**	**4.41 (1.97–9.83)**	**77.4 (19.1–313.4)**	**7.26 (2.46–21.4)**	0.77 (0.34–1.74)
Sex	Male	1	1	1	1	1
	Female	**0.62 (0.44–0.87)**	0.65 (0.42–1.01)	1.05 (0.81–1.35)	0.77 (0.52–1.13)	**0.42 (0.26–0.68)**
Ethnicity	Fijian of Indian Descent	1	1	1	1	1
	iTaukei & others	**2.68 (1.79–4.03)**	**1.80 (1.09–2.96)**	**2.0 (1.52–2.61)**	**2.45 (1.52–3.94)**	1.71 (0.97–3.04)
Comorbidity§	Index = 0	1	1	1	1	1
	Index = 1	**5.92 (3.56–9.87**	**3.65 (1.89-7.02)**	**2.92 (2.03–4.21)**	**5.21 (2.59–10.5)**	1.20 (0.36–3.98)
	Index ≥2	**6.41 (4.15–9.89)**	**3.64 (2.00–6.59)**	**2.51 (1.86–3.89)**	**9.14 (5.36–15.6)**	1.97 (0.85–4.54)

∗Bold font indicates P<0.05 while underlined font indicates P<0.001. ‡At least one hospitalisation for heart failure, stroke, atrial fibrillation or endocarditis. §Modified Charlson Comorbidity Index[26] calculated without points for heart failure and stroke.

11

Table S6: **Population-based rates of hospitalisations for RHD complications per 100,000 person-years in the general population.**

Age (years)	Any complication*		Heart failure		Atrial fibrillation		Fiji
	Events	Rate (95% CI)	Events	Rate (95% CI)	Events	Rate (95% CI)	Popn†
0-4	0	-	0	-	0	-	84926
5-9	124	31 (25.8-37)	8	2 (0.9-3.9)	1	0.2 (0-1.4)	80020
10-14	174	41.4 (35.4-48)	21	5 (3.1-7.6)	2	0.5 (0.1-1.7)	84145
15-20	159	39.2 (33.4-45.8)	38	9.4 (6.6-12.9)	15	3.7 (2.1-6.1)	81040
20-24	181	44.3 (38.1-51.2)	34	8.3 (5.8-11.6)	26	6.4 (4.2-9.3)	81722
25-29	188	50.4 (43.4-58.1)	39	10.5 (7.4-14.3)	36	9.6 (6.8-13.4)	74621
30-34	161	49.8 (42.4-58.1)	31	9.6 (6.5-13.6)	28	8.7 (5.8-12.5)	64629
35-39	136	47.3 (39.7-56)	39	13.6 (9.6-18.5)	35	12.2 (8.5-16.9)	57501
40-44	171	59.8 (51.2-69.5)	78	27.3 (21.6-34.1)	48	16.8 (12.4-22.3)	57158
45-49	177	69.5 (59.6-80.5)	91	35.7 (28.8-43.8)	77	30.2 (23.8-37.8)	50960
50-54	147	72.6 (61.3-85.3)	66	32.6 (25.2-41.5)	63	31.1 (23.9-39.8)	40493
55-59	120	76 (63-90.9)	75	47.5 (37.4-59.5)	64	40.5 (31.2-51.8)	31584
60-64	98	80 (65-97.5)	66	53.9 (41.7-68.6)	44	35.9 (26.1-48.2)	24490
65-69	48	56.1 (41.4-74.4)	26	30.4 (19.9-44.6)	36	42.1 (29.5-58.3)	17102
Aged 0–69 years	-	45.4 (43.4-47.5)	-	14.7 (13.6-16.0)	-	11.4 (10.4-12.5)	

*All hospitalisations for heart failure, stroke, atrial fibrillation or endocarditis. †Estimated midpoint population in Fiji.

For peer review only

Table S6: **Continued**

Age (years)	Ischaemic stroke Events	Ischaemic stroke Rate (95% CI)	Infective endocarditis Events	Infective endocarditis Rate (95% CI)	Fiji Popn*
0-4	0	-	0	-	84926
5-9	0	0 (0-0.9)	7	1.7 (0.7-3.6)	84145
10-14	3	0.7 (0.1-2.1)	9	2.1 (1-4.1)	84145
15-20	4	1 (0.3-2.5)	9	2.2 (1-4.2)	81040
20-24	7	1.7 (0.7-3.5)	3	0.7 (0.2-2.1)	81722
25-29	3	0.8 (0.2-2.3)	10	2.7 (1.3-4.9)	74621
30-34	9	2.8 (1.3-5.3)	9	2.8 (1.3-5.3)	64629
35-39	5	1.7 (0.6-4.1)	8	2.8 (1.2-5.5)	58501
40-44	17	5.9 (3.5-9.5)	8	2.8 (1.2-5.5)	57158
45-49	9	3.5 (1.6-6.7)	3	1.2 (0.2-3.4)	50960
50-54	14	6.9 (3.8-11.6)	4	2 (0.5-5.1)	40493
55-59	8	5.1 (2.2-10)	1	0.6 (0-3.5)	31584
60-64	7	5.7 (2.3-11.8)	0	0 (0-3)	24490
65-69	7	8.2 (3.3-16.9)	0	0 (0-4.3)	17102
Aged 0–69 years		2.2 (1.8-2.7)		1.7 (1.3-2.1)	

*Estimated midpoint population in Fiji.

12

For peer review only

Table S7: **Incidence rate ratios estimates with 95% confidence intervals for new nonfatal complications of RHD in the general population in multivariate Poisson regression*.**

		Any complication†	Heart failure	Atrial fibrillation	Ischaemic stroke	Infective endocarditis
Age (yrs)	5-14	1	1	1	1	1
	15-39	2.00 (1.75-2.28)	**4.37 (2.95-6.48)**	**32.8 (10.4-103.6)**	**6.74 (2.04-22.3)**	**1.9 (1.04-3.46)**
	40-69	3.04 (2.66-3.48)	**16.0 (10.9-23.3)**	**130 (41.6-408)**	**25.2 (7.88-80.8)**	1.28 (0.63-2.61)
Sex	Male	1	1	1	1	1
	Female	**1.43 (1.30-1.57)**	0.93 (0.79-1.09)	**1.51 (1.25-1.82)**	1.27 (0.84-1.92)	0.63 (0.39-1.02)
Ethnicity	Fijian of Indian Descent	1	1	1	1	1
	iTaukei & others	**1.39 (1.27-1.53)**	1.18 (1.0-1.38)	**1.30 (1.07-1.59)**	**1.79 (1.13-2.84)**	**2.11 (1.18-3.75)**

*Bold font indicates P<0.05 while underlined font indicates P<0.001. †At least one hospitalisation for heart failure, stroke, atrial fibrillation or endocarditis.

For peer review only

For peer review only - http://bmjopen.bmj.com/site/about/guidelines.xhtml

Table S8: **Estimates of years of life lost (YLL), years lived with disability (YLD) and disability adjusted life years (DALYs) attributable to RHD complications per 100,000 person-years in the general population.**

Age	Deaths	Death rate	YLL	YLL rate	YLD	YLD rate	DALYs rate	Fiji popn.*	GBD. std (%)†
0-4	0	0	0	0	0	0	0	84926	10.18
5-9	1.5	0.4	130.1	32.5	43.7	10.9	43.5	80020	9.68
10-14	27.5	6.5	2171.3	516.1	88.9	21.1	537.2	84145	8.95
15-19	28.6	7.1	2116	522.2	81.3	20.1	542.3	81040	8.38
20-24	26.5	6.5	1834.3	448.9	70.4	17.2	466.1	81722	8.02
25-29	24.2	6.5	1554.3	416.6	67.8	18.2	434.8	74621	7.78
30-34	32.7	10.1	1933.1	598.2	58.3	18	616.3	64629	7.33
35-39	34	11.8	1844.8	641.6	54.7	19	660.7	57501	6.78
40-44	33.9	11.8	1670.2	584.4	66.8	23.4	607.8	57158	6.09
45-49	46	18	2042.3	801.5	66.5	26.1	827.6	50960	5.47
50-54	27.7	13.7	1097.3	542	59.9	29.6	571.5	40493	4.87
55-59	30.2	19.1	1053.7	667.2	42.2	26.7	693.9	31584	4.25
60-64	25.6	20.9	774.9	632.8	35.9	29.3	662.2	24490	3.6
65-69	16.2	19	416.1	486.7	18.6	21.8	508.4	17102	2.91
Aged 0–69 years (95% CI)		8.6 (7.7-9.5)		448.9 (442.5-455.4)		18.2 (16.9-19.5)	467.1 (460.5-473.7)		
Age-standardised‡ (95% CI)		9.4 (8.5-10.3)		438.0 (431.8-444.4)		18.4 (17.1-19.7)	456.4 (450.1-462.9)		

*Estimated midpoint population in Fiji. †GBD world population age standard[30]; ‡Study rates for the population aged 65–69 years applied to population aged ≥70 years.

For peer review only

Table S9: **Hazard ratio estimates with 95% confidence intervals for risk of death associated with hospitalisation for nonfatal complications of RHD in the hospital cohort in a multivariate Cox regression analysis.**

Variable	Level	HR (95% CI)	P
Heart failure	Before	1.0	
with or without atrial fib.	After	6.57 (4.76–9.06)	<0.001
Atrial fibrillation	Before	1.0	
without heart failure	After	2.86 (1.75–4.67)	<0.001
Ischaemic stroke	Before	1.0	
	After*	6.56 (3.82–11.2)	<0.001
Infective endocarditis	Before	1.0	
	After*	4.48 (2.22–9.02)	<0.001
Age (yrs)	5-14	1.0	
	15-39	1.40 (0.88–2.24)	0.16
	40-69	1.67 (1.01–2.75)	0.046
Sex	Male	1.0	
	Female	0.83 (0.63–1.09)	0.17
Ethnicity	Fijian of Indian Descent	1.0	
	iTaukei & others	0.78 (0.57–1.07)	0.12
Comorbidity†	Index < 2	1.0	
	Index ≥2	1.49 (1.10–2.03)	0.011
Residence	Urban	1.0	
	Rural	1.18 (0.90–1.54)	0.23

*Hazard within 28 days of admission for complication. †Modified Charlson Comorbidity Index[26] calculated without points for heart failure and stroke.

For peer review only - http://bmjopen.bmj.com/site/about/guidelines.xhtml

STROBE Statement—checklist of items that should be included in reports of observational studies

	!"#$%<br>&'(%	)#*'$$#+,-".'+%	/-0#%%<br>&'(%	)#1#2-+"%"#3"%45'$%<br>$-+67*5.8"%
**9."1#%-+,%-:7"5-*"%**	1	(*a*) Indicate the study's design with a commonly used term in the title or the abstract	1	
		(*b*) Provide in the abstract an informative and balanced summary of what was done and what was found	2	
		**!+"5',6*".'+%**		%
Background/rationale	2	Explain the scientific background and rationale for the investigation being reported	4-5	
Objectives	3	State specific objectives, including any prespecified hypotheses	5	
		**;#"<',7%**		%
Study design	4	Present key elements of study design early in the paper	4	
Setting	5	Describe the setting, locations, and relevant dates, including periods of recruitment, exposure, follow-up, and data collection	6-7	
Participants	6	(*a*) *Cohort study*—Give the eligibility criteria, and the sources and methods of selection of participants. Describe methods of follow-up	6-7	
		(*b*)%*Cohort study*—For matched studies, give matching criteria and number of exposed and unexposed	N/A	
Variables	7	Clearly define all outcomes, exposures, predictors, potential confounders, and effect modifiers. Give diagnostic criteria, if applicable	7	
Data sources/ measurement	8*	For each variable of interest, give sources of data and details of methods of assessment (measurement). Describe comparability of assessment methods if there is more than one group	6-7	
Bias	9	Describe any efforts to address potential sources of bias	7-8	
Study size	10	Explain how the study size was arrived at	N/A	

! "#$%#&' ()"#)#'*$)+, -'

For peer review only

Quantitative variables	11	Explain how quantitative variables were handled in the analyses. If applicable, describe which groupings were chosen and why	7-8
Statistical methods	12	(*a*) Describe all statistical methods, including those used to control for confounding	7-8
		(*b*) Describe any methods used to examine subgroups and interactions	7-8
		(*c*) Explain how missing data were addressed	8
		(*d*) *Cohort study*—If applicable, explain how loss to follow-up was addressed	7
		(*e*) Describe any sensitivity analyses	N/A
		**) #761"7%**	
Participants	13*	(a) Report numbers of individuals at each stage of study—eg numbers potentially eligible, examined for eligibility, confirmed eligible, included in the study, completing follow-up, and analysed	9-10
		(b) Give reasons for non-participation at each stage	9
		(c) Consider use of a flow diagram	Suppl. Fig. S1
Descriptive data	14*	(a) Give characteristics of study participants (eg demographic, clinical, social) and information on exposures and potential confounders	Table 1
		(b) Indicate number of participants with missing data for each variable of interest	Table 1
		(c) *Cohort study*—Summarise follow-up time (eg, average and total amount)	9
Outcome data	15*	*Cohort study*—Report numbers of outcome events or summary measures over time	10
Main results	16	(*a*) Give unadjusted estimates and, if applicable, confounder-adjusted estimates and their precision (eg, 95% confidence interval). Make clear which confounders were adjusted for and why they were included	10-13
		(*b*) Report category boundaries when continuous variables were categorized	Table 1
		(*c*) If relevant, consider translating estimates of relative risk into absolute risk for a meaningful time period	N/A

! "#$%#&’ ()"#)#’*$)+, -’

For peer review only - http://bmjopen.bmj.com/site/about/guidelines.xhtml

Other analyses	17	Report other analyses done—eg analyses of subgroups and interactions, and sensitivity analyses	13-14
**=.7*677.'+**			
Key results	18	Summarise key results with reference to study objectives	14-15
Limitations	19	Discuss limitations of the study, taking into account sources of potential bias or imprecision. Discuss both direction and magnitude of any potential bias	17
Interpretation	20	Give a cautious overall interpretation of results considering objectives, limitations, multiplicity of analyses, results from similar studies, and other relevant evidence	18
Generalisability	21	Discuss the generalisability (external validity) of the study results	15-16
**>"<#5.+4'5 $ -".'+**			
Funding	22	Give the source of funding and the role of the funders for the present study and, if applicable, for the original study on which the present article is based	20

*Give information separately for cases and controls in case-control studies and, if applicable, for exposed and unexposed groups in cohort and cross-sectional studies.

&'"#? An Explanation and Elaboration article discusses each checklist item and gives methodological background and published examples of transparent reporting. The STROBE checklist is best used in conjunction with this article (freely available on the Web sites of PLoS Medicine at http://www.plosmedicine.org/, Annals of Internal Medicine at http://www.annals.org/, and Epidemiology at http://www.epidem.com/). Information on the STROBE Initiative is available at www.strobe-statement.org.

For peer review only

Population-Based Assessment of Cardiovascular Complications of Rheumatic Heart Disease in Fiji: a Record-linkage Analysis

Journal:	*BMJ Open*
Manuscript ID	bmjopen-2022-070629.R1
Article Type:	Original research
Date Submitted by the Author:	01-Feb-2023
Complete List of Authors:	Parks, Tom; Imperial College London; University of Oxford<br>Narube, Litia; Fiji National University College of Medicine Nursing and Health Sciences<br>Perman, Mai Ling; Fiji National University College of Medicine Nursing and Health Sciences<br>Sakumeni, Kelera; Fiji Ministry of Health & Medical Services<br>Fong, James; Fiji Ministry of Health & Medical Services<br>Engelman, Daniel; Murdoch Children's Research Institute<br>Colquhoun, Samantha ; Australian National University<br>Steer, Andrew; Murdoch Children's Research Institute<br>Kado, Joseph; Fiji Ministry of Health & Medical Services; Telethon Kids Institute
**Primary Subject Heading**:	Cardiovascular medicine
Secondary Subject Heading:	Epidemiology, Global health, Infectious diseases
Keywords:	Valvular heart disease < CARDIOLOGY, Heart failure < CARDIOLOGY, Stroke < NEUROLOGY, PAEDIATRICS, OBSTETRICS, PUBLIC HEALTH

I, the Submitting Author has the right to grant and does grant on behalf of all authors of the Work (as defined in the below author licence), an exclusive licence and/or a non-exclusive licence for contributions from authors who are: i) UK Crown employees; ii) where BMJ has agreed a CC-BY licence shall apply, and/or iii) in accordance with the terms applicable for US Federal Government officers or employees acting as part of their official duties; on a worldwide, perpetual, irrevocable, royalty-free basis to BMJ Publishing Group Ltd ("BMJ") its licensees and where the relevant Journal is co-owned by BMJ to the co-owners of the Journal, to publish the Work in this journal and any other BMJ products and to exploit all rights, as set out in our licence.

The Submitting Author accepts and understands that any supply made under these terms is made by BMJ to the Submitting Author unless you are acting as an employee on behalf of your employer or a postgraduate student of an affiliated institution which is paying any applicable article publishing charge ("APC") for Open Access articles. Where the Submitting Author wishes to make the Work available on an Open Access basis (and intends to pay the relevant APC), the terms of reuse of such Open Access shall be governed by a Creative Commons licence – details of these licences and which Creative Commons licence will apply to this Work are set out in our licence referred to above.

Other than as permitted in any relevant BMJ Author's Self Archiving Policies, I confirm this Work has not been accepted for publication elsewhere, is not being considered for publication elsewhere and does not duplicate material already published. I confirm all authors consent to publication of this Work and authorise the granting of this licence.

For peer review only

Population-Based Assessment of Cardiovascular Complications of Rheumatic Heart Disease in Fiji: a Record-linkage Analysis

Tom Parks MD*[1,2], Litia Narube MBBS[3], Mai Ling Perman MBBS[3], Kelera Sakumeni MBBS[4], James J. Fong MBBS[4], Daniel Engelman PhD[5,6], Samantha M. Colquhoun PhD[7], Andrew C. Steer PhD[5,6], and Joseph Kado MBBS[4,8,9]

[1]Imperial College London, London, UK
[2]University of Oxford, Oxford, UK
[3]Fiji National University, Suva, Fiji
[4]Fiji Islands Ministry of Health and Medical Services, Suva, Fiji
[5]Murdoch Children's Research Institute, Melbourne Australia
[6]University of Melbourne, Melbourne, Australia
[7]Australian National University, Canberra, Australia
[8]Telethon Kids Institute, Perth, Australia
[9]University of Western Australia, Perth, Australia

*Correspondence to: t.parks@imperial.ac.uk

For peer review only - http://bmjopen.bmj.com/site/about/guidelines.xhtml

1 Abstract

Objective: To determine population-based rates of nonfatal complications of rheumatic heart disease (RHD).

Design: Retrospective cohort study based on multiple sources of routine clinical and administrative data amalgamated by probabilistic record-linkage.

Setting: Fiji, an upper-middle-income country, where most of the population has access to government-funded health care services.

Participants: National cohort of 2,116 patients with clinically apparent RHD aged 5-69 years during 2008-2012.

Primary and secondary outcome measures: The primary outcome was hospitalisation for any of heart failure, atrial fibrillation, ischaemic stroke and infective endocarditis. Secondary outcomes were first hospitalisation for each of the complications individually in the national cohort as well as in hospital (n=1300) and maternity (n=210) subsets. Information on outcomes was obtained from discharge diagnoses coded in the hospital patient information system. Population-based rates were obtained using relative survival methods with census data as the denominator.

Results: Among 2,116 patients in the national cohort (median age, 23.3 years; 57.7% female), 546 (25.8%) were hospitalised for an RHD complication, a substantial proportion of all cardiovascular admissions in the country during this period in those aged 0–40 years (heart failure, 210/454, 46.2%; ischaemic stroke 31/134, 23.3%). Absolute numbers of RHD complications peaked during the third decade of life with higher population-based rates in women compared to men (incidence rate ratio 1.4, 95% CI 1.3–1.6, P<0.001). Hospitalisation for any RHD complication was associated with substantially increased risk of death (hazard ratio, 5.4, 95% CI, 3.4–8.8, P<0.001), especially after the onset of heart failure (hazard ratio, 6.6, 95% CI, 4.8–9.1, P<0.001).

Conclusions: Our study defines the burden of RHD-attributable morbidity in the general population of Fiji, potentially reflecting the situation in low- and middle-income countries worldwide. Hospitalisation for an RHD complication is associated with markedly increased risk of death, re-emphasising the importance of effective early prevention.

For peer review only

For peer review only - http://bmjopen.bmj.com/site/about/guidelines.xhtml

Strengths and limitations of this study

- Our study was based on probabilistic record-linkage providing an efficient means to derive population-based estimates of disease burden from routinely collected data.
- We used relative survival methods to estimate rates of RHD-attributable morbidity in the general populations since this approach helps separate out events that are unrelated to the disease of interest.
- We demonstrate the comparability of the hospital subset of our cohort to key previous hospital-based studies, including the landmark REMEDY study, set in Asia and Africa, greatly increasingly the generalisability of our population-based estimates beyond Fiji.
- The routinely collected data used in our study are likely to contain some inaccuracies, including in the precision of coding of discharge diagnoses. However, there is little reason to suspect the relevant diagnostic codes would have been used differently in patients with and without RHD.
- Reflecting external factors, we recognise that several years have now passed since the period from which our multiple sources of routine data were available. Nonetheless, they continue to provide invaluable insight into the burden of RHD in low resource settings, chiefly due to the scarcity of comparable estimates from elsewhere.

For peer review only

For peer review only - http://bmjopen.bmj.com/site/about/guidelines.xhtml

2 Introduction

Rheumatic heart disease (RHD) is a chronic disease of the heart valves that predominantly affects children and young adults in low- and middle-income countries (LMICs) [1]. The disease is a consequence of an aberrant immune response to *Streptococcus pyogenes* infection causing inflammation and scarring of the heart valves [2], which manifests clinically through complications such as heart failure, ischaemic stroke and early death [3]. Secondary prevention is achievable using long-term antibiotic prophylaxis [4], although implementation remains challenging in most settings where the disease is endemic [5]. Moreover, there remains no vaccine against the causative *S. pyogenes* [6].

In recent years, there has been growing recognition that RHD causes a substantial burden of disease and disability globally [7–10], culminating in 2018 in a World Health Assembly resolution calling for a coordinated global response [11]. Global summary estimates by the Global Burden of Disease (GBD) project suggest RHD accounts for approximately 305,000 deaths and 10.7 million disability-adjusted life years annually, the majority of these occurring LMICs [7,9,12]. Notwithstanding their importance in providing impetus for increased investment in RHD prevention, such estimates remain problematic because of the scarcity of good quality data available from LMIC settings, where the disease is endemic [13]. For example, to our knowledge, our previous record-linkage analysis set in Fiji remains the only population-based estimate of RHD-attributable mortality from an LMIC [14]. Moreover, data pertaining to the rates of nonfatal complications, such as heart failure and stroke, among RHD patients living in LMICs are particularly scarce [7].

One compelling exception to this pattern was the landmark REMEDY study, published in 2016, a prospective, hospital-based registry that defined rates of death, heart failure and stroke among 3,343 symptomatic RHD patients enrolled across 12 African countries, India and Yemen [15]. However, as the authors of that study noted, since the patients were mostly enrolled at tertiary referral hospitals, the rates observed cannot be easily generalised to the wider pool of RHD patients away from hospital

For peer review only

settings [15]. Furthermore, neither REMEDY nor more recent studies of RHD progression provide estimates of population-based rates of complications of RHD [16–19], which are needed to improve current global summary estimates as well as to inform the design, implementation and evaluation of disease control efforts [13,20].

Therefore we sought to assess the burden and calculate nationwide population-based rates of RHD-attributable morbidity in Fiji, a middle-income country in the Western Pacific.

3 Methods

This study investigated RHD-attributable morbidity among patients with clinically apparent RHD in Fiji during 2008-2012. Patients were identified from a cohort previously ascertained by probabilistic record linkage (Supplementary Figure S1) [14]. In this analysis, we investigated the four major complications of RHD: heart failure, atrial fibrillation, ischaemic stroke and infective endocarditis [3], which together we term 'any complication'. We then used census data to calculate age-specific RHD-attributable rates, as well as estimates of years lived with disability (YLDs), for the wider population.

We also examined two subsets of the 'national' cohort in further detail (Supplementary Figure S1). The first, termed the 'hospital' cohort, comprised patients with at least one hospital attendance during follow-up, allowing assessment of the relationship between RHD-attributable morbidity and mortality in this setting, as well as comparison with previously reported hospital-based studies. The second, termed the 'maternity' cohort, comprised women with at least one diagnostic code indicating pregnancy or childbirth during follow-up, enabling a description of the RHD-attributable morbidity and mortality during pregnancy, childbirth and up to one year postpartum in the cohort [21]. The study was approved by the Fiji National Research Ethics Review Committee (2013-89) and the Oxford University Tropical Research Ethics Committee (1055-13).

For peer review only

For peer review only - http://bmjopen.bmj.com/site/about/guidelines.xhtml

3.1 Setting

At the time of the study, the population of Fiji was approximately 840,000, comprising two major ethnic groups, Indigenous iTaukei and Fijians of Indian Descent [22,23]. Government-funded hospital services are available free-of-charge including consultations, admissions, laboratory investigations and radiological examinations [24]. A high proportion of pregnant women give birth in hospital [24].

3.2 Cohort

We used sources of data and record-linkage methods as described previously [14]. Briefly, we used a probabilistic record-linkage procedure to amalgamate data across the four datasets: a hospital patient information system, a disease control register, echocardiography clinic registers and a database of death certificates. After extensive cleaning and standardisation, we used multiple identifiers including registration numbers, names, sex, ethnicity, birth dates, and locality of residence to find pairs of records that referred to the same individual. In its final configuration, our procedure identified known duplications in the hospital patient information system with sensitivity of 91.4% and specificity of 99.9% [14].

For this current analysis, we focussed on patients with clinically apparent RHD reported in one or more of the hospital patient information system, the disease control register and the echocardiography clinic registers. As before, we restricted the analysis to patients aged 5–69 years and the period 2008–2012 for which the most data was available [14]. We obtained information on complications resulting in hospitalisation, as well as pregnancy outcomes from the coded discharge diagnoses reported in the hospital patient information system. Data were available from all government funded hospitals, including the two regional and one national referral hospitals, but not from the smaller private sector

For peer review only

For peer review only - http://bmjopen.bmj.com/site/about/guidelines.xhtml

[24] The completeness and quality of Fiji's hospital patient information system data have been independently investigated previously [25] and the diagnostic codes used to define RHD and its complications are summarised in Supplementary Table S1. We also used this system to obtain information on co-morbidities using a system based on the Charlson Comorbidity Index [26] but excluding heart failure and stroke from the calculation since these were outcomes of interest in this study.

The primary outcome was first hospitalisation for any complication defined by the date of hospital admission when the relevant diagnostic code first appeared in the patient's records. The secondary outcomes were hospitalisation for each of the complications individually. We assumed no loss to follow-up because we expected the patient information system would include most hospitalisations in the country during 2008–2012 [24,25].

3.3 Statistical methods

We used survival methods to calculate cumulative incidence rates for each outcome in the cohort. Notably, the available coded discharge diagnoses data did not reliably distinguish between new and past diagnoses. Accordingly, to minimise the risk of counting events that occurred prior to the study, we limited the incidence rate calculations for the three more frequent outcomes, any complication, heart failure and atrial fibrillation, to the fourth and fifth year of the study. However, for the two remaining outcomes, ischaemic stroke and infective endocarditis, we used all the data available because: 1) these less frequent outcomes occurred at a comparatively constant rate throughout the study (Supplementary Figure S2); and 2) preliminary estimates based on the fourth and fifth year of the study tended to be imprecise. Having excluded nine individuals with missing sex information, we then used competing-risks regression (Stata *stcrreg* command) [27] to examine the impact of age (in three categories), sex and ethnicity (both as recorded in the hospital patient information system), and comorbidity on the risk of complications with death as the competing failure event.

For peer review only - http://bmjopen.bmj.com/site/about/guidelines.xhtml

To obtain rates of RHD-attributable morbidity in the general population, we used relative survival methods by applying background rates of hospitalisation to the cohort by sex, ethnicity and age in thirteen five-year age categories [28]. Background rates for each diagnosis were estimated by Poisson regression using the counts of hospitalisations from the patient information system and concurrent denominator population estimates published by the Fiji Bureau of Statistics [22]. Specifically, we calculated age-specific rates for each complication using the observed minus the expected number (i.e. the excess) as the numerator divided by the population estimates as the denominator with 95% confidence intervals (CI) calculated using Poisson.

Years lived with disability were calculated by applying disability weights used by the GBD study to the person-time of the cohort during follow-up using weights: heart failure (severe), 0.179; stroke (moderate), 0.316; atrial fibrillation (symptomatic), 0.224; infective endocarditis (severe), 0.133; uncomplicated RHD, 0.049 [12]. Where a patient had experienced more than one complication, disability weights were combined using a multiplicative model, as previously described [29]. To estimate disability-adjusted life-years (DALYs), we updated our previous estimates of years of life lost (YLLs) [14] using the GBD 2019 theoretical minimum risk life table [12]. In addition to summary estimates for the population of Fiji aged 0–69 years, we also calculated age-standardised rates using the GBD world population age standard [30] with rates of death and disability from our study for the population aged 65–69 years applied to the population aged ≥70 years.

To assess the relationship between RHD-attributable morbidity and mortality in the cohort, we focussed on the hospital cohort subset, defined by at least one attendance during follow-up at one of the two regional hospitals or the one national referral hospitals in the country. Alongside the opportunity for comparison with previously reported hospital-based studies, this subgroup had the advantage of a well-defined starting point for the time-to-event analysis (i.e. the earliest recorded hospital or outpatient clinic attendance in the study period at which a diagnosis of RHD was recorded) as well as the availability of additional clinical parameters for analysis (e.g. disease severity and presence of mitral stenosis). For each complication, having excluded five individuals with missing

For peer review only - http://bmjopen.bmj.com/site/about/guidelines.xhtml

sex information, we split the follow-up time at the earliest point at which the complication occurred (Stata *stsplit* command) and plotted Kaplan-Meier estimates of survival based on the resulting variable to illustrate risk before and after the event occurred. We then used a Cox regression model to measure the relative impact of each of the complications, using the Schoenfeld residuals to test the proportional-hazards assumption (Stata *phtest* command). All models included potential confounders – age, sex, ethnicity, comorbidity and rural residence – and evaluated interactions using likelihood ratio tests. Statistical analyses were conducted using Stata, version 12 (StataCorp, College Station, TX).

3.3 Patient and public involvement

Patients and/or the public were not involved in the design, or conduct, or reporting, or dissemination plans of this research.

4 Results

From our previously reported cohort of 2,619 patients [14], we excluded 92 individuals known only for acute rheumatic fever, 302 identified only through echocardiographic screening, and 109 for whom the only available information was a death certificate (Supplementary Figure S1). The cohort comprised 2,116 patients with clinically apparent RHD followed for a median of 5.0 years. Most were aged 5-39 years (74.6%) and more than half were female (57.7%, Table 1).

4.1 RHD-attributable morbidity in the cohort

There were 2,116 patients in the national cohort, totalling 9,439 years of person-time. During follow-up, 1,335 (63.1%) patients were hospitalised a total of 2,896 times, equating to 1.4% of the total 205,427 admissions for any cause in the population of Fiji aged 5–69 years during 2008–2012. The

For peer review only

For peer review only - http://bmjopen.bmj.com/site/about/guidelines.xhtml

most frequent discharge diagnostic codes were heart failure (22.5% of admissions) and atrial fibrillation (17.6%) (Supplementary Table S2).

In total, 546 patients (25.8%) were admitted with a discharge diagnosis consistent with a complication of RHD (Figure 1). More specifically, 349 patients (16.5%) were admitted with heart failure, 268 (12.7%) for atrial fibrillation, 104 (4.9%) for ischaemic stroke and 73 (3.4%) for endocarditis (Supplementary Table S3). Furthermore, 220 (10.4%) patients had admissions for more than one complication of which heart failure and atrial fibrillation in 154 (7.3%) followed by ischaemic stroke and atrial fibrillation in 46 (2.2%) were the most frequent combinations. The yearly cumulative incidence rate of any complication was 4.6% per year (95% CI 3.9–5.4) with rates of all complications except infective endocarditis increasing with age (Table 2 & Supplementary Table S4).

For peer review only

Table 1: **Baseline characteristics of patients by sex in the national, hospital and maternity cohorts.** [*]

	National (n = 2,116)		Hospital (n = 1,300)		Maternity (n = 210)
	Male (n = 892)	Female (n = 1,215)	Male (n = 547)	Female (n = 748)	Female (n = 210)
Age, n (%)					
5–14 years	356 (39.9)	326 (26.8)	197 (36.0)	179 (23.9)	5 (0.95)
15–39 years	312 (35.0)	577 (47.5)	191 (34.9)	356 (47.6)	202 (96.2)
40–69 years	224 (25.1)	312 (25.7)	159 (29.1)	213 (28.5)	6 (2.9)
Ethnicity, n (%)					
iTaukei (Indigenous)	527 (59.1)	775 (63.8)	332 (60.7)	500 (66.8)	151 (72.3)
Fijian of Indian Descent	294 (33.0)	366 (30.1)	166 (30.4)	201 (26.9)	41 (19.6)
Other	71 (8.0)	74 (6.1)	49 (9.0)	47 (6.3)	17 (8.1)
Data sources, n (%)					
Hospital admission	375 (42.0)	562 (46.3)	324 (59.2)	461 (61.6)	135 (64.3)
Echocardiography clinic	427 (47.9)	608 (50.0)	341 (62.3)	480 (64.2)	129 (61.4)
Control programme	523 (58.6)	682 (56.1)	185 (33.8)	223 (29.8)	92 (43.8)
Comorbidity†, n (%)					
Index = 0	763 (85.5)	1,047 (86.2)	435 (79.5)	615 (82.2)	195 (92.9)
Index = 1	48 (5.4)	46 (3.8)	41 (7.5)	33 (4.4)	7 (3.3)
Index ≥2	81 (9.1)	122 (10.0)	71 (13.0)	100 (13.4)	8 (3.8)
Rural residence, n (%)	400 (44.8)	515 (42.4)	245 (44.8)	301 (40.2)	64 (30.5)
Pregnancy, n (%)	0	210 (17.3)	0	155 (20.7)	210 (100)

[*] Sex information missing for nine patients in the national cohort and five patients in the hospital cohort. †Modified Charlson Comorbidity [26] calculated without points awarded for heart failure and stroke.

For peer review only - http://bmjopen.bmj.com/site/about/guidelines.xhtml

Table 2: **Cumulative incidence rates per 100 person-years with 95% confidence intervals of nonfatal complications of RHD within the national cohort[*].**

Age	Sex	Any complication[†]	Heart failure	Atrial fibrillation	Ischaemic stroke	Infective endocarditis
5-14 yrs	Male	1.7 (0.8–3.6)	1.2 (0.5–2.9)	0	0.2 (0.0–0.7)	0.9 (0.5–1.6)
	Female	1.1 (0.4–2.9)	0.8 (0.3–2.5)	0	0.2 (0.0–0.7)	0.5 (0.2–1.2)
15-39 yrs	Male	4.9 (3.5–7.0)	2.7 (1.7–4.2)	2.1 (1.2–3.5)	1.0 (0.6–1.6)	1.4 (0.9–2.1)
	Female	2.8 (2.0–4.0)	1.5 (0.9–2.4)	1.5 (0.9–2.4)	0.7 (0.4–1.0)	0.5 (0.3–0.9)
40-69 yrs	Male	12.8 (9.9–18.3)	6.5 (4.2–10.3)	6.6 (4.3–10.2)	2.9 (2.0–4.2)	1.0 (0.5–1.9)
	Female	10.3 (7.5–14.2)	4.8 (3.2–7.4)	8.5 (6.2–11.8)	2.6 (1.8–3.6)	0.4 (0.2–0.9)

[*] Rates are based on hospitalisation during the fourth or fifth year for any complications, heart failure and atrial fibrilation but otherwise at any stage during the study. Rates for the entire study period are given in Supplementary Table S4. [†] At least one hospitalisation for heart failure, stroke, atrial fibrillation or endocarditis.

For peer review only - http://bmjopen.bmj.com/site/about/guidelines.xhtml

In a multivariate analysis with adjustment for age and comorbidity, complications occurred more frequently in men than in women (hazard ratio, HR, 1.6, 95% CI 1.2–2.3, P=0.005, Supplementary Table S5). In addition, Indigenous iTaukei or other ethnicity was associated with a more than two-fold increased risk of complications compared to Fijians of Indian Descent (HR, 2.7, 95% CI 1.8–4.0, P<0.001). Across the complications, residential location (urban/rural) had no impact on risk (P>0.20).

4.2 RHD-attributable morbidity in the general population

To estimate RHD-attributable morbidity in the general population, we compared rates of hospitalisation for complications to those in the general population. Among the population 5-69 years in Fiji during 2008–2012, 651 of 4756 (13.7%) hospitalisations with a discharge diagnosis consistent with heart failure, 509 of 2225 (22.9%) with atrial fibrillation, 127 of 1927 (6.6%) with ischaemic stroke and 73 of 108 (67.6%) with endocarditis occurred in patients with an underlying diagnosis of RHD (Figure 2). However, before 40 years of age, 210 of 454 (46.2%) hospitalisations with heart failure, 143 of 288 (49.7%) with atrial fibrillation and 31 of 134 (23.3%) for stroke were associated with an underlying diagnosis of RHD.

We then calculated rates of RHD-attributable morbidity in the general population (Figure 2). For the Fiji population aged 0–69 years, we estimated that for every 100,000 person-years there were 14.7 (95% CI 13.6–16.0) excess hospitalisations for heart failure, 11.4 for atrial fibrillation (95% CI 10.4–12.5), 2.2 for ischaemic stroke (95% CI 1.8–2.7) and 1.7 for infective endocarditis (95% CI 1.3–2.1) each year (Supplementary Table S6). While absolute numbers of RHD-attributable complications peaked in the third decade, the incidence of all but infective endocarditis increased with age. In a multivariate analysis, incidence rates of complications were lower outside the Fijian of Indian Descent population (Supplementary Table S7), with this difference being most marked for stroke (incidence rate ratio, IRR, 1.8, 95% CI 1.1–2.8, P=0.013) and infective endocarditis (IRR 2.1, 95% CI 1.2–3.8, P=0.012). However, contrary to the within cohort risk calculations, overall incidence of complications

For peer review only - http://bmjopen.bmj.com/site/about/guidelines.xhtml

was higher in women than in men (IRR 1.4, 95% CI 1.3-1.6, P<0.001), an effect that appeared driven predominantly by higher rates of atrial fibrillation (IRR 1.5, 95% CI 1.3-1.8, P<0.001).

We estimated a yearly rate of 18.2 RHD-attributable YLDs (95% CI 16.9–19.5) per 100,000 person-years in the Fiji population aged 0–69 years (Supplementary Table S8). Rates of RHD-attributable disability increased during adolescence and thereafter increased only marginally (Supplementary Figure S3). Heart failure alone accounted for 18.7% of YLDs with 28.6% due to other complications while the remaining 52.7% were attributable to RHD without complications. Combined with our previous analyses of RHD-attributable mortality for the same population [14], we calculated 467.1 DALYs (95% CI 460.5–473.7) per 100,000 person-years were lost to RHD in the Fiji population aged 0–69 years during the study period.

4.3 Relationship between RHD-attributable morbidity and mortality

There were 1300 patients in the hospital cohort (a subset of the national cohort) with at least one admission to one of the two regional or one national referral hospitals or outpatient attendance at the national referral hospital during follow-up (Table 1). This cohort accounted for 3,118 person-years with a median follow-up time of 2.4 years and 238 deaths (18.5%), equating to an overall yearly death rate of 7.2% (95% CI 6.3–8.2).

Median survival time was markedly shortened after admission with a complication to 3.6 years after any complication, 2.1 years after heart failure and 2.4 years after stroke (Figure 3). Adjusted for age, sex, ethnicity, comorbidity and rural residence, admission for any complication was associated with a five-fold increased risk of death for the remainder of follow-up (HR 5.4, 95% CI 3.4–8.8, P<0.001). Moreover, admission for heart failure, atrial fibrillation, stroke and infective endocarditis all had independent effects on risk of death, although the independent effect of atrial fibrillation was only apparent before the onset of heart failure (Supplementary Table S9). However, while heart failure and

For peer review only - http://bmjopen.bmj.com/site/about/guidelines.xhtml

atrial fibrillation had a lasting effect on risk of death, that associated with stroke and infective endocarditis was short-lived and only apparent within the first 28 days of admission.

4.4 RHD-attributable morbidity and mortality during maternity

There were 210 women in the maternity cohort with at least one diagnostic code indicating pregnancy or childbirth during follow-up (17.3% of 1,215 women in the entire cohort), 204 women with a single pregnancy and six women with two pregnancies. A total of 164 deliveries (75.9%) were managed at the national referral hospital, 46 deliveries (21.3%) at two regional referral hospitals and the remaining six at subdivisional or unknown locations. The median age at delivery was 27.0 years (IQR 22.8–31.9).

A total of 123 deliveries (56.9%) had diagnostic codes indicating a complication of labour or delivery of which the most frequent were perineal laceration (74 deliveries), fetal distress (23 deliveries) and umbilical cord complications (10 deliveries). The mode of delivery was indicated in only 50 records (six indicating caesarean section, three forceps or vacuum extraction and the remainder spontaneous). Ten of 210 women (4.8%) had a diagnostic code suggesting a prior or co-existent complication of RHD including three each with heart failure, ischaemic stroke and infective endocarditis and two with atrial fibrillation (none of these women died during follow-up). However, overall, 11 of 210 women (5.2%) died during follow-up at a median age 32.2 years, including five after the onset of at least one RHD complication. While none of these deaths occurred within 42 days of delivery, three occurred within one year of delivery (i.e. late maternal deaths) of which one death was attributable to RHD and two to other causes.

For peer review only - http://bmjopen.bmj.com/site/about/guidelines.xhtml

5 Discussion

In this nationwide, population-based study, we provide evidence that RHD underlies a substantial proportion of cardiovascular morbidity among children and young adults in Fiji, likely reflecting the situation in LMICs worldwide.

Our analyses provide a considerable amount of new information about the epidemiology of nonfatal RHD complications in LMICs. First, we observed a high burden of RHD-attributable disability among adolescents and young adults, especially women of childbearing age, with women in the general population at greater risk than men overall. Second, we found a large burden of disability in the population attributable not only to heart failure but also to other complications of RHD – together these other complications accounted for a greater number of YLDs than heart failure alone, which is striking given the reliance on the latter in global summary estimates [7,9]. Third, we found that all four of the nonfatal complications that we assessed were independently associated with an increased risk of death. Consequently, notwithstanding the substantial rates of nonfatal RHD complications in this setting, our study indicates that the dominant public health impact of RHD in this setting is premature death rather than chronic disability.

There are few previously published studies available for comparison to our population-based estimates. Nonetheless, comparison of estimates from our hospital cohort with those from previous hospital-based studies helps benchmark our findings. Focusing on the REMEDY study [15] there is a clear resemblance between the rates of new events in our hospital cohort during the initial two years of follow-up and those reported by the REMEDY investigators (Supplementary Figure S4) [15]. This is illustrated, for example, by the 14.3% of hospital cohort patients who died within two years compared to 12.5% of patients in REMEDY for upper middle-income countries. This comparability underscores the vital importance of our ascertainment of patients away from the hospital setting and increases the generalisability of our findings beyond the Pacific region.

For peer review only

An additional comparison is with the recently reported ERASE project from Australia, which also identified cases using record-linkage [18]. The most relevant comparison is the yearly cumulative incidence rate of heart failure within a year of diagnosis, which at 3.2% in the Australian study [18] is similar to the 2.5% estimated from our study with overlapping confidence intervals. Additionally, the rates of nonfatal complications that we report are similar to those from a hospital-based cohort reported from the high income Pacific nation of New Caledonia [31]. Beyond this, we and others have previously highlighted the burden of RHD complications in hospital- and community-based cohorts in the Pacific and beyond [17,19,32–34]. While broadly consistent with our findings, none of these prior studies provided the population-based estimates of disease burden needed to better define the scale of the RHD problem in LMICs.

This analysis extends our previous investigation of cardiac complications of childbirth in Fiji at the national referral hospital [35]. However, the number of complications detected here are much lower than ascertained by case record review. For example, prior or coexistent heart failure was noted in only three women (2%) in the current study compared to 23% in the case note review [35]. While it plausible rates are lower in the general population compared to the national referral hospital, it is also likely these lower rates reflect variable recording of non-obstetric diagnostic codes in the hospital patient information system in this group. Accordingly, the rates of complications that we report here are low relative to previous studies [36–38] and should be interpreted with caution. Nonetheless, the substantial number of deaths in this group during the remainder of follow-up is a stark reminder of the need for new approaches to tackle the burden of RHD among women of child-bearing age [39].

Finally, the GBD study provides the key comparison for our population-based estimates, including those specific to Fiji for the same period [7,9,12]. Our point estimate of YLDs at 18.2 per 100,000 in the population aged 0–69 years is 40% lower than that made by GBD, which, inferred from prevalence, pertains only to heart failure. Importantly, we found a markedly different age-distribution of YLDs to that suggested by GBD, with relatively fewer RHD-attributable YLDs in young adults, likely reflecting markedly shortened survival times after the onset of complications. Accordingly,

For peer review only

For peer review only - http://bmjopen.bmj.com/site/about/guidelines.xhtml

96% of DALYs in our study were the result of YLLs, reflecting RHD-attributable deaths among adolescents and young adults [14], and our DALYs estimate at 467.1 per 100,000 in the population aged 0–69 years is 28% higher than that from GBD. Moreover, since this may reflect the situation in LMICs more widely, it is our view that these discrepancies mandate further work to measure RHD-attributable death and disability in other settings to inform future GBD estimates.

Although our results appear reasonable, they have some limitations. First, notwithstanding the value of linked routine data for epidemiological analyses, there is likely to be some inaccuracy in our dataset, including in the precision of coding of discharge diagnoses. Nonetheless, since there is little reason to suspect diagnostic codes would have been used differently in patients with and without RHD, we expect this limitation to have limited impact on our population-based estimates of RHD-attributable morbidity. Future studies might increase accuracy by using algorithms developed to improve ascertainment of RHD cases from routine data, although these algorithms have yet to be validated in data from LMICs [40].

Second, our data did not distinguish new from pre-existing complications and complications were frequently present at or shortly after diagnosis. To counter this, we limited estimates of the incidence of heart failure (as well as 'any complication' and atrial fibrillation) to the fourth and fifth years of the study on the presumption that almost all of those who developed these events prior to this period would either have either died or been detected during the first three years of follow-up. This 'clearance' period is shorter than the eight years in a recent Australian study [18] but supported by event rates in our cohort (Supplementary Figure S2). In addition, to calculate population-based rates, we made comparisons with hospitalisation data from the general population from the same period, which we expect to comprise a similar make up of new and pre-existing complications.

Third, our data lack important information of clinical relevance, including recurrence of acute rheumatic fever, detailed echocardiographic correlates of risk, and the impact of therapeutics such as penicillin prophylaxis and anticoagulation. Finally, reflecting external factors, we recognise that

For peer review only - http://bmjopen.bmj.com/site/about/guidelines.xhtml

several years have now passed since the period from which our multiple sources of routine data were available. Nonetheless, we maintain they provide invaluable insight into the burden of morbidity and mortality attributable to RHD in low resource settings, not least because it is likely to be a number of years before interventions targeting reduction of childhood *S. pyogenes* infections and improved provision of penicillin prophylaxis impact the burden of disease in the adult population in Fiji.

5.1 Conclusions

By summarising burden of nonfatal complications of RHD in Fiji, our study underscores the assertion that effective control of RHD in LMICs could have a substantial impact on the global burden of cardiovascular morbidity and mortality in the young. However, without significant investment in efforts to improve knowledge of RHD epidemiology in LMICs worldwide, targeting and evaluating the impact of control measures will remain problematic, including, ultimately, delivery of vaccines against the causative *S. pyogenes*.

For peer review only - http://bmjopen.bmj.com/site/about/guidelines.xhtml

For peer review only

6 Data availability

The authors did not generate the primary dataset which is the property of the Fiji Ministry of Health and Medical Services. Researchers wishing to access Fiji Ministry of Health data should send a request in writing to the Permanent Secretary for Health, Fiji Ministry of Health and Medical Services, Dinem House, 88 Amy Street, PO Box 2223, Government Buildings, Suva, Fiji Islands (see also: http://www. health.gov.fj). Use of Fiji Ministry of Health data would also be subject to approval by the Fiji National Health Research Committee. The study protocol is available for download at: http://users.ox.ac.uk/~clme1250/rhd_epi/. An earlier version of this manuscript was released as a preprint at: http://dx.doi.org/10.2139/ssrn.4237745.

7 Ethical approval

The study was approved by the Fiji National Research Ethics Review Committee (2013-89) and the Oxford University Tropical Research Ethics Committee (1055-13).

8 Contributions

TP, JK, MLP, JF, SMC, ACS conceived the study. TP, JK, LN, SMC, ACS acquired and analysed the underlying data. TP, JK, LN, MLP, KS, JF, DE, SMC, ACS interpreted the findings. TP wrote the first draft of the manuscript. All authors contributed to and critically revised the manuscript. All authors approved the final version.

9 Acknowledgements

We thank the Ministry of Health and Medical Services and the National Disease Control Programme in Fiji for providing the datasets as well as their employees who compiled them, particularly those at the Ministry of Health Information Unit. We thank and recognise the contribution of the late Dr Isimeli Tukana, formerly Head of Wellness and National Adviser for Non-Communicable Disease at the Fiji Ministry of Health and Medical

For peer review only

Services, for his vital contribution to conception and design of the study. We also thank Dr Anne E Hannay (née Miller), Mr Brenton Ward, Dr Rachel Heenan, Ms Tuliana Cua and Ms Maureen Ah Kee for their contribution to the data collection.

10 Funding

This work was supported by the Medical Research Council UK (G1100449 to TP), the British Medical Association Foundation for Medical Research (Research Grant 2012 to TP), the Sir Halley Stewart Trust (Research Grant 2013 to TP), the National Institute for Health and Care Research UK (CL-2020-21-001 to TP) and the Wellcome Trust (222098/Z/20/Z to TP). These funders had no role in study design, data collection and analysis, decision to publish, or preparation of the manuscript.

This research was funded in whole, or in part, by the Wellcome Trust (222098/Z/20/Z). For the purpose of Open Access, the author has applied a CC BY public copyright licence to any Author Accepted Manuscript version arising from this submission.

11 Competing interests

None declared.

For peer review only - http://bmjopen.bmj.com/site/about/guidelines.xhtml

12 References

1. Remenyi B, ElGuindy A, Smith SC, Yacoub M, and Holmes DR. Valvular aspects of rheumatic heart disease. The Lancet 2016;387:1335 –1346. doi: 10.1016/s01406736(16)00547-x.

2. Carapetis JR, McDonald M, and Wilson NJ. Acute rheumatic fever. The Lancet 2005;366:155 –168. doi: 10.1016/s0140-6736(05)66874-2.

3. Marijon E, Mirabel M, Celermajer DS, and Jouven X. Rheumatic heart disease. The Lancet 2012;379:953 –964. doi: 10.1016/s0140-6736(11)61171-9.

4. Beaton A, Okello E, Rwebembera J, et al. Secondary Antibiotic Prophylaxis for Latent Rheumatic Heart Disease. New England Journal of Medicine 2021;386:230– 40. doi: 10.1056/nejmoa2102074.

5. Engelman D, Mataika RL, Kado JH, et al. Adherence to secondary antibiotic prophylaxis for patients with rheumatic heart disease diagnosed through screening in Fiji. Tropical Medicine & International Health 2016;21:1583–91. doi: 10.1111/tmi. 12796.

6. Vekemans J, Gouvea-Reis F, Kim JH, et al. The Path to Group A Streptococcus Vaccines: World Health Organization Research and Development Technology Roadmap and Preferred Product Characteristics. Clinical Infectious Diseases 2019;69:877–83. doi: 10.1093/cid/ciy1143.

7. Watkins DA, Johnson CO, Colquhoun SM, et al. Global, Regional, and National Burden of Rheumatic Heart Disease, 1990-2015. The New England Journal of Medicine 2017;377:713 –722. doi: 10.1056/nejmoa1603693.

8. Sliwa K, White A, Milan P, Mocumbi AO, Zilla P, and Wood D. Momentum builds for a global response to rheumatic heart disease. European Heart Journal 2018;39:4229 –4232. doi: 10.1093/eurheartj/ehy763.

9. Roth GA, Mensah GA, Johnson CO, et al. Global Burden of Cardiovascular Diseases and Risk Factors, 1990–2019 Update From the GBD 2019 Study. Journal of the American College of Cardiology 2020;76:2982–3021. doi: 10.1016/j.jacc.2020. 11.010.

10. Ghamari S, Abbasi-Kangevari M, Moghaddam SS, et al. Rheumatic Heart Disease Is a Neglected Disease Relative to Its Burden Worldwide: Findings From Global Burden of Disease 2019. Journal of the American Heart Association 2022;11:e025284. doi: 10.1161/jaha.122.025284.

11. White A. WHO Resolution on rheumatic heart disease. European Heart Journal 2018;39:4233 –4233. doi: 10.1093/eurheartj/ehy764.

12. GBD 2019 Diseases and Injuries Collaborators, Vos T, Lim SS, et al. Global burden of 369 diseases and injuries in 204 countries and territories, 1990–2019: a systematic analysis for the Global Burden of Disease Study 2019. The Lancet 2020;396:1204– 22. doi: 10.1016/s0140-6736(20)30925-9.

13. Abouzeid M, Wyber R, Vincente SL, et al. Time to tackle rheumatic heart disease: Data needed to drive global policy dialogues. Global public health 2018;14:1 –13. doi: 10.1080/17441692.2018.1515970.

14. Parks T, Kado J, Miller AE, et al. Rheumatic Heart Disease-Attributable Mortality at Ages 5-69 Years in Fiji: A Five-Year, National, Population-Based Record-Linkage Cohort Study. PLoS Neglected Tropical Diseases 2015;9:e0004033. doi: 10.1371/ journal.pntd.0004033.

15. Zühlke L, Karthikeyan G, Engel ME, et al. Clinical Outcomes in 3343 Children and Adults With Rheumatic Heart Disease From 14 Low- and Middle-Income Countries. Circulation 2016;134:1456–66. doi: 10.1161/circulationaha.116.024769.

For peer review only - http://bmjopen.bmj.com/site/about/guidelines.xhtml

16. Noubiap JJ, Agbor VN, Bigna JJ, Kaze AD, Nyaga UF, and Mayosi BM. Prevalence and progression of rheumatic heart disease: a global systematic review and metaanalysis of population-based echocardiographic studies. Scientific Reports 2019;9:17022. doi: 10.1038/s41598-019-53540-4.

17. Jaiteh LES, Drammeh L, Anderson ST, et al. Rheumatic heart disease in The Gambia: clinical and valvular aspects at presentation and evolution under penicillin prophylaxis. BMC Cardiovascular Disorders 2021;21:503. doi: 10.1186/s12872021-02308-8.

18. Stacey I, Hung J, Cannon J, et al. Long-term outcomes following rheumatic heart disease diagnosis in Australia. European Heart Journal Open 2021;1:oeab035. doi: 10.1093/ehjopen/oeab035.

19. Zimmerman M, Kitooleko S, Okello E, et al. Clinical outcomes of children with rheumatic heart disease. Heart 2022;108:633–8. doi: 10.1136/heartjnl-2021320356.

20. Zühlke LJ and Steer AC. Estimates of the Global Burden of Rheumatic Heart

Disease. Global Heart 2013;8:189–95. doi: 10.1016/j.gheart.2013.08.008.

21. Ronsmans C, Graham WJ, and group oboTLMSSs. Maternal mortality: who, when, where, and why. The Lancet 2006;368:1189–200. doi: 10.1016/s0140-6736(06) 69380-x.

22. Fiji Bureau of Statistics. 2007 Census of the Population. Tech. rep. 2008. URL:

http://www.statsfiji.gov.fj.

23. Fiji Bureau of Statistics. Fiji Population & Housing Census 2017. Tech. rep. Fiji Bureau of Statistics, 2017. url: https://www.statsfiji.gov.fj/index.php/ census-2017.

24. Roberts G, Irava W, Tuiketei T, et al. The Fiji Islands Health System Review. Health Systems in Transition 2011;1. Ed. by Tulloch J. URL: https://apps.who.int/iris/bitstream/handle/10665/207503/9789290615439_eng.pdf.

25. Reyburn R, Nand D, Nguyen C, et al. Validation of administrative data to estimate vaccine impact: Audit of the Fiji hospital admissions electronic database, 2007–2011 & 2014–2015. Vaccine 2017;35:6416–21. doi: 10.1016/j.vaccine.2017.09.091.

26. Charlson ME, Pompei P, Ales KL, and MacKenzie CR. A new method of classifying prognostic comorbidity in longitudinal studies: development and validation. Journal of Chronic Diseases 1987;40:373 – 383.

27. Fine JP and Gray RJ. A Proportional Hazards Model for the Subdistribution of a Competing Risk. Journal of the American Statistical Association 1999;94:496. doi: 10.2307/2670170.

28. Dickman PW, Sloggett A, Hills M, and Hakulinen T. Regression models for relative survival. Statistics in Medicine 2004;23:51 –64. doi: 10.1002/sim.1597.

29. Murray CJL, Ezzati M, Flaxman AD, et al. GBD 2010: design, definitions, and metrics. The Lancet 2012;380:2063 –2066. doi: 10.1016/s0140-6736(12)61899-6.

30. GBD 2017 Causes of Death Collaborators, Roth GA, Abate D, et al. Global, regional, and national age-sex-specific mortality for 282 causes of death in 195 countries and territories, 1980–2017: a systematic analysis for the Global Burden of Disease Study 2017. The Lancet 2018;392:1736–88. doi: 10.1016/s0140-6736(18) 32203-7.

31. Mirabel M, Tafflet M, Noël B, et al. Newly diagnosed rheumatic heart disease among indigenous populations in the Pacific. Heart 2015-12;101:1901 –1906. doi: 10.1136/heartjnl-2015-308237.

For peer review only

For peer review only - http://bmjopen.bmj.com/site/about/guidelines.xhtml

32. Steer AC, Kado J, Jenney AWJ, et al. Acute rheumatic fever and rheumatic heart disease in Fiji: prospective surveillance, 2005–2007. Medical Journal of Australia 2009;190:133–5. doi: 10.5694/j.1326-5377.2009.tb02312.x.

33. Sriharibabu M, Himabindu Y, and Kabir Z. Rheumatic heart disease in rural south India: A clinico-observational study. Journal of Cardiovascular Disease Research 2013;4:25–9. doi: 10.1016/j.jcdr.2013.02.011.

34. Okello E, Longenecker CT, Beaton A, Kamya MR, and Lwabi P. Rheumatic heart disease in Uganda: predictors of morbidity and mortality one year after presentation. BMC Cardiovascular Disorders 2017;17:20. doi: 10.1186/s12872-016-0451-8.

35. Narube L, Fong J, Parks T, Ekeroma AJ, and Kubuabola I. Pregnancy outcomes in women with heart disease at the Colonial War Memorial Hospital, Suva, Fiji. Pacific Journal of Reproductive Health 2016;1. doi: 10.18313/pjrh.2016.910.

36. Hagen IMv, Thorne SA, Taha N, et al. Pregnancy Outcomes in Women With Rheumatic Mitral Valve Disease. Circulation 2018;137:806–16. doi: 10.1161/circulationaha.117.032561.

37. Beaton A, Okello E, Scheel A, et al. Impact of heart disease on maternal, fetal and neonatal outcomes in a low-resource setting. Heart 2019;105:755. doi: 10.1136/ heartjnl-2018-313810.

38. Baghel J, Keepanasseril A, Pillai AA, Mondal N, Jeganathan Y, and Kundra P. Prediction of adverse cardiac events in pregnant women with valvular rheumatic heart disease. Heart 2020;106:1400–6. doi: 10.1136/heartjnl-2020-316648.

39. Vaughan G, Dawson A, Peek M, et al. Rheumatic Heart Disease in Pregnancy: New Strategies for an Old Disease? Global Heart 2021;16:84. doi: 10.5334/gh.1079.

40. Bond-Smith D, Seth R, Klerk Nd, et al. Development and Evaluation of a Prediction Model for Ascertaining Rheumatic Heart Disease Status in Administrative Data. Clinical Epidemiology 2020;12:717–30. doi: 10.2147/clep.s241588.

For peer review only

For peer review only - http://bmjopen.bmj.com/site/about/guidelines.xhtml

Figure Legends:

Figure 1: **Hospitalisations for nonfatal complications in the cohort.** The proportion of national cohort hospitalised for each complication of RHD are shown on the left with number of reaching each outcome indicated at the end of each bar. Cumulative incidence rates of each complication are shown on the right with 95% confidence intervals (CI).

Figure 2: **Hospitalisation for heart failure, atrial fibrillation and stroke in the general population by age.** The number of hospitalisations for each complication are shown on the left with the fraction attributable to RHD coloured red. Population-based rates of RHD-attributable hospitalisation for each complication are shown on the right with 95% confidence intervals (CI) against the lefthand axis and the proportion attributable to RHD as a percentage against the righthand axis.

Figure 3: **Survival probability following hospitalisation for any complication, heart failure, atrial fibrillation and ischaemic stroke in the hospital cohort.** For each event, survival probability is plotted with 95% confidence intervals before and after hospitalisation with the number at risk at each timepoint in the table.

For peer review only

For peer review only - http://bmjopen.bmj.com/site/about/guidelines.xhtml

Figure 1: **Hospitalisations for nonfatal complications in the cohort.** The proportion of national cohort hospitalised for each complication of RHD are shown on the left with number of reaching each outcome indicated at the end of each bar. Cumulative incidence rates of each complication are shown on the right with 95% confidence intervals (CI).

a) Proportion of the cohort b) Cumulative incidence

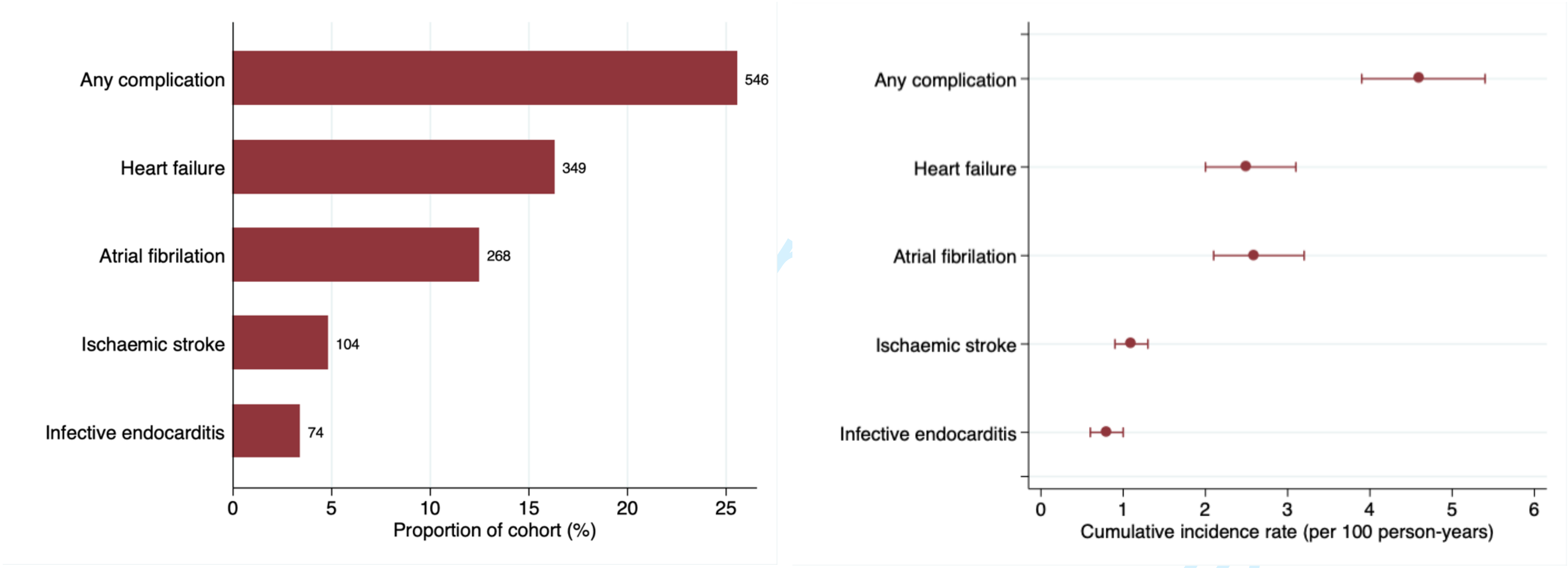

For peer review only - http://bmjopen.bmj.com/site/about/guidelines.xhtml

Figure 2: **Hospitalisation for heart failure, atrial fibrillation and stroke in the general population by age.** The number of hospitalisations for each complication are shown on the left with the fraction attributable to RHD coloured red. Population-based rates of RHD-attributable hospitalisation for each complication are shown on the right with 95% confidence intervals (CI) against the lefthand axis and the proportion attributable to RHD as a percentage against the righthand axis.

a) Heart failure (count)

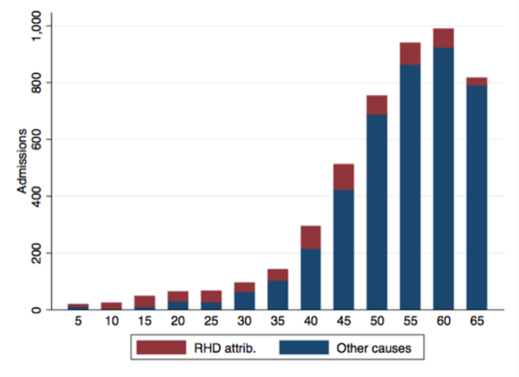

b) Heart failure (rate)

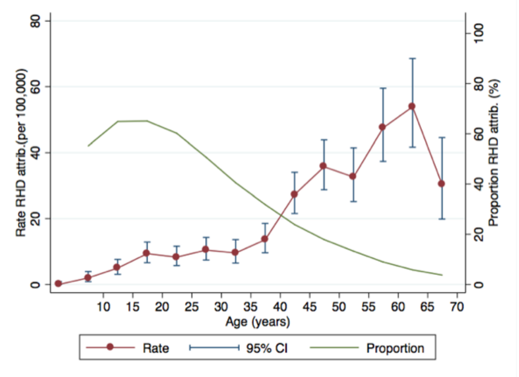

c) Atrial fibrillation (count)

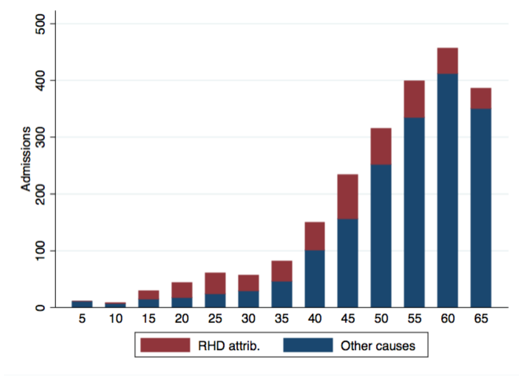

d) Atrial fibrillation (rate)

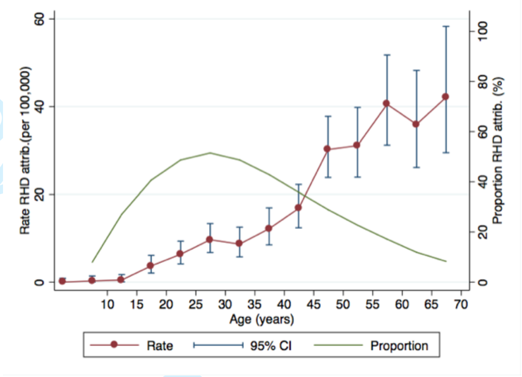

e) Ischaemic stroke (count)

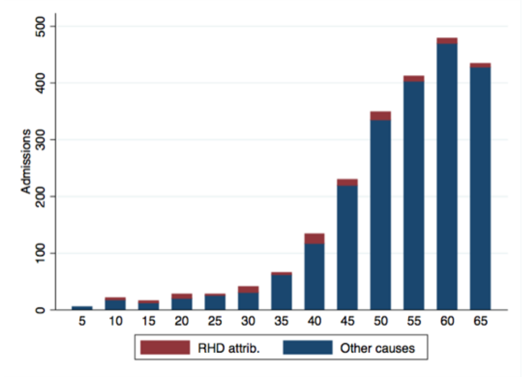

f) Ischaemic stroke (rate)]

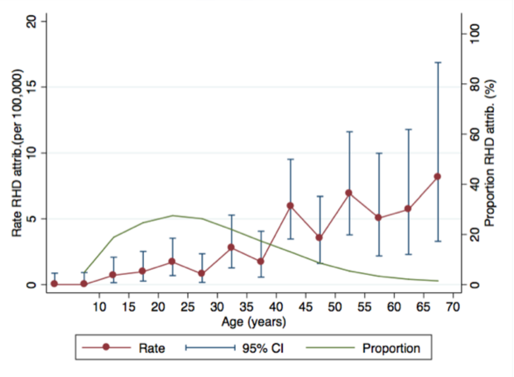

Figure 3: **Survival probability following hospitalisation for any complication, heart failure, atrial fibrillation and ischaemic stroke in the hospital cohort.** For each event, survival probability is plotted with 95% confidence intervals before and after hospitalisation with the number at risk at each timepoint in the table.

a) Any complication

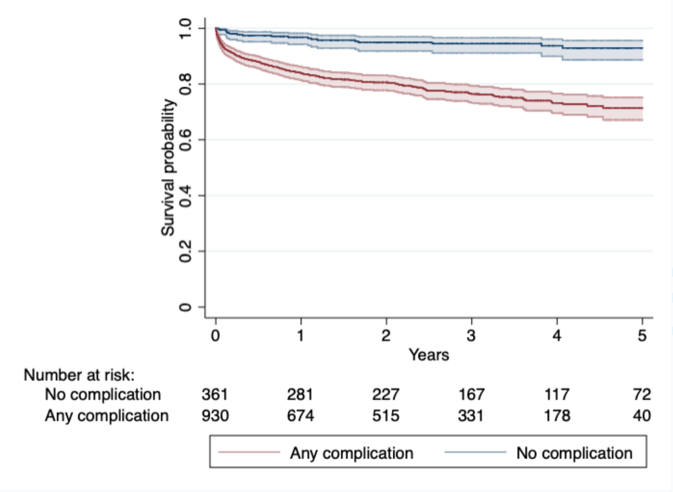

b) Heart failure

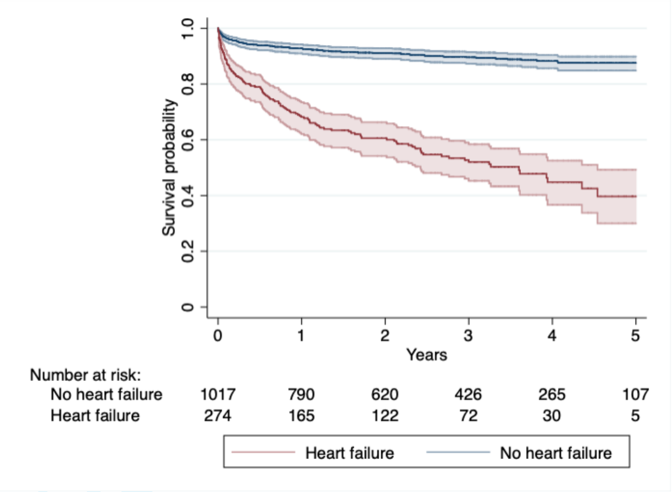

c) Atrial fibrillation

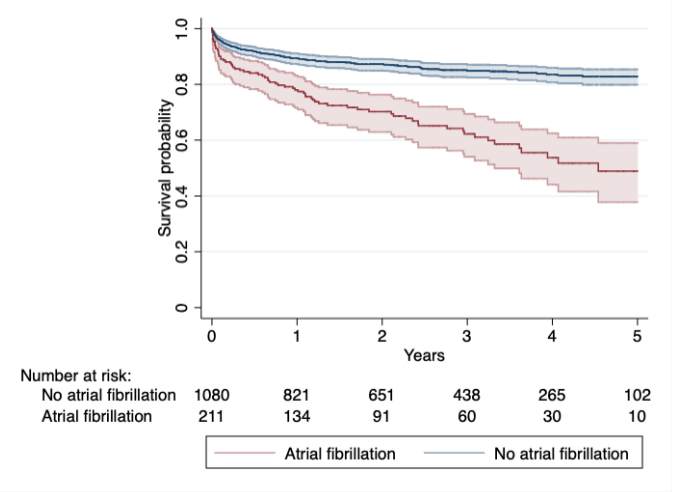

d) Ischaemic stroke

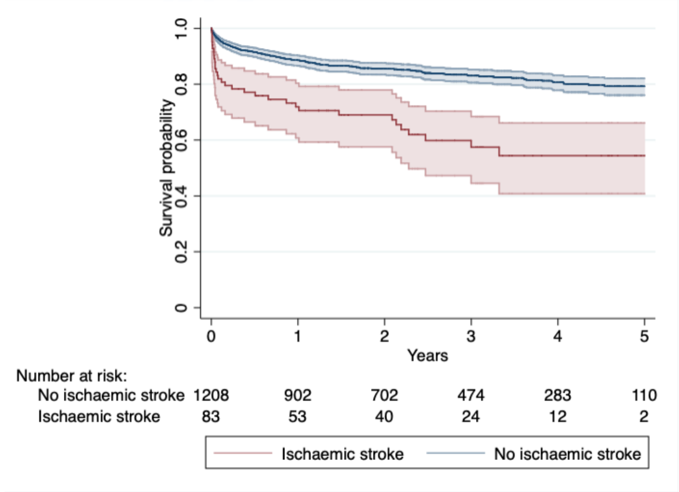

For peer review only - http://bmjopen.bmj.com/site/about/guidelines.xhtml

Supplementary Material

For peer review only

For peer review only - http://bmjopen.bmj.com/site/about/guidelines.xhtml

Figure S1: **Overview of the study design.** Our primary analyses focused on the subset of patients from our previous reported study with clinically apparent RHD.[14] For further analyses, we defined two subsets: patients with at least one hospital attendance during follow-up, termed the hospital cohort, and women with at least one pregnancy during follow-up, termed the maternity cohort.

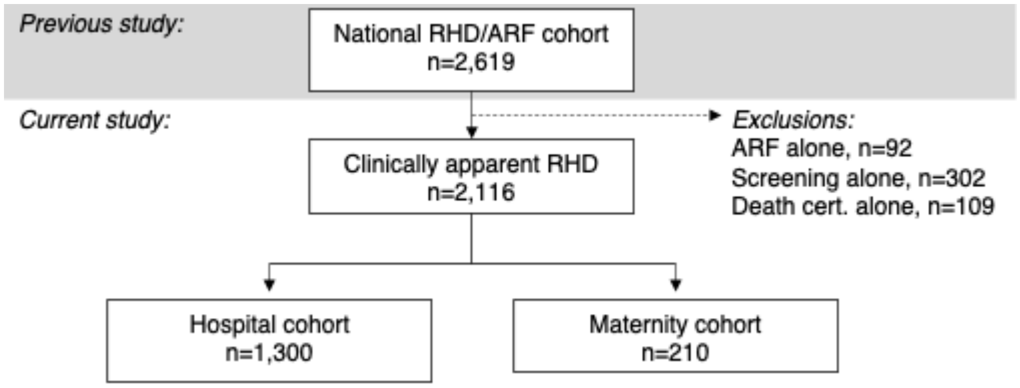

ARF, acute rheumatic fever.

For peer review only - http://bmjopen.bmj.com/site/about/guidelines.xhtml

Figure S2: **Cumulative incidence of any complication, heart failure, atrial fibrillation, ischaemic stroke and infective endocarditis in the national cohort.**

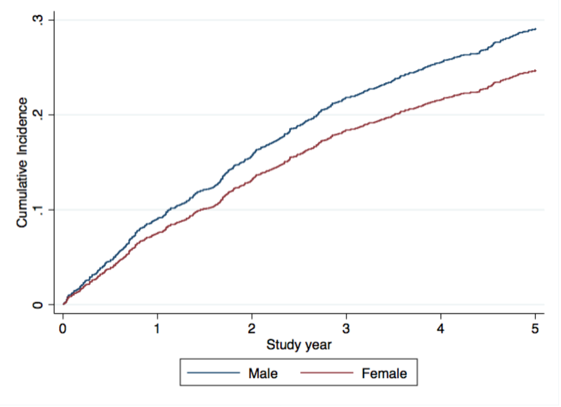

(a) All events

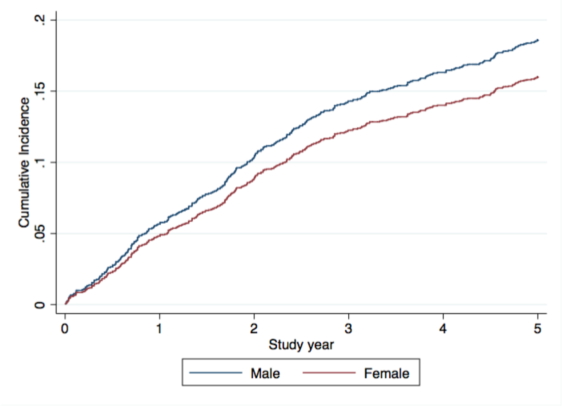

(b) Heart failure

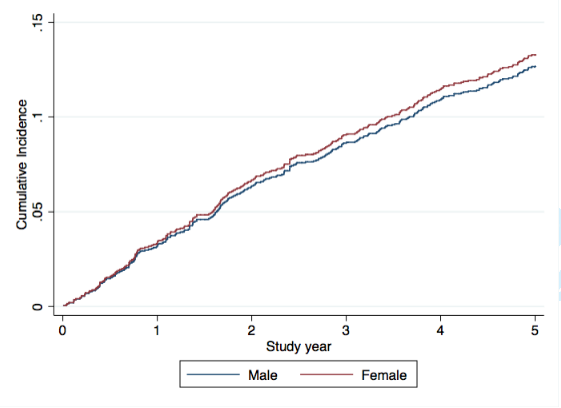

(c) Atrial fibrillation

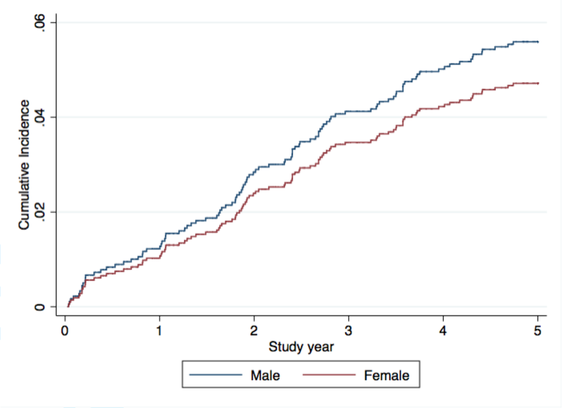

(d) Ischaemic stroke

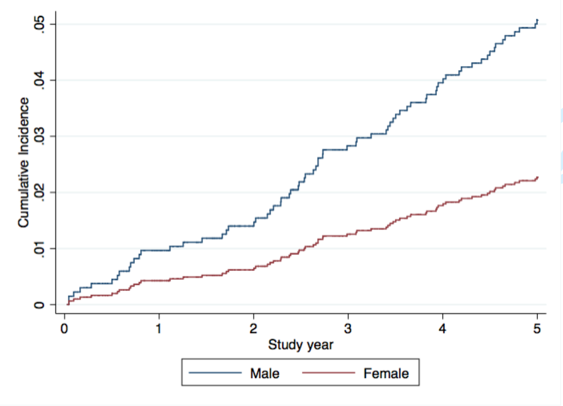

(e) Infective endocarditis

For peer review only - http://bmjopen.bmj.com/site/about/guidelines.xhtml

Figure S3: **Population-based rates of RHD-attributable disability from the study compared to estimates for the same period from the Global Burden of Disease (GBD) study.**[7,9,12]

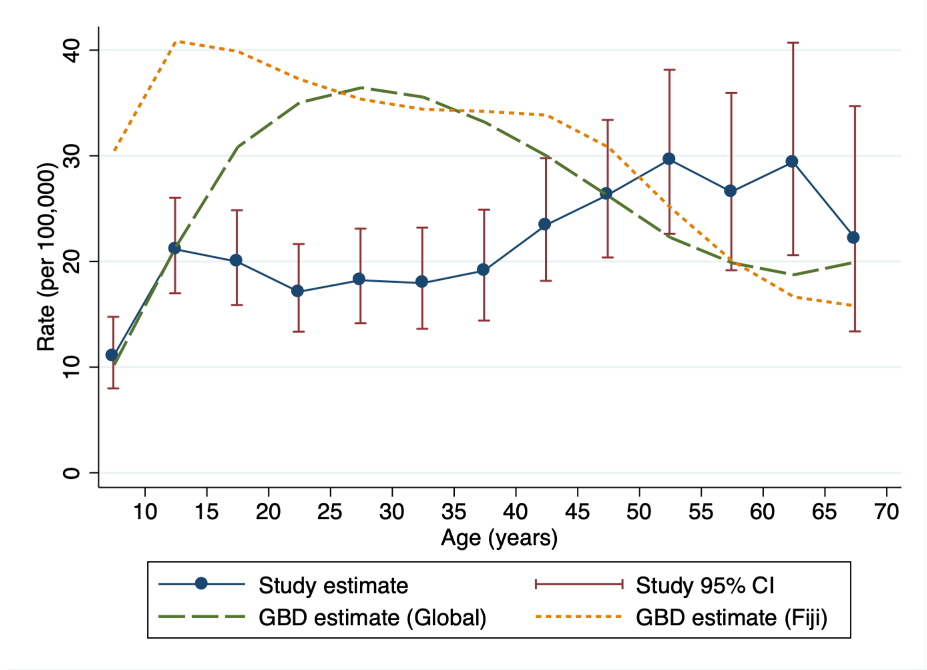

(a) Years lived with disability (YLDs)

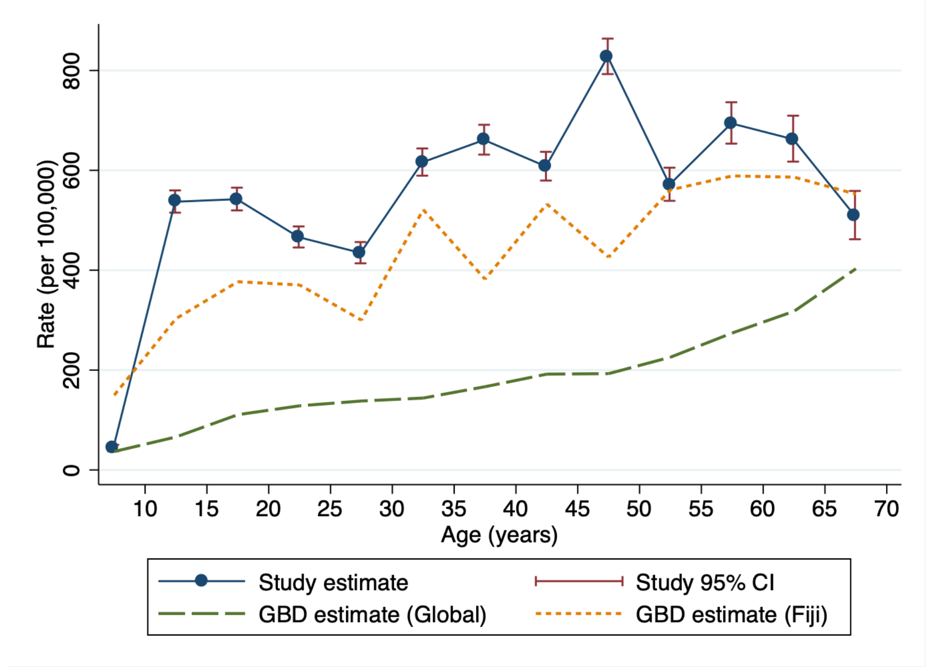

(b) Disability adjusted life-years (DALYs)

For peer review only - http://bmjopen.bmj.com/site/about/guidelines.xhtml

Figure S4: **Proportion of the hospital cohort with new RHD complications at 2 years follow-up compared to clinical outcomes at 2 years follow-up in the REMEDY cohort.**[15]

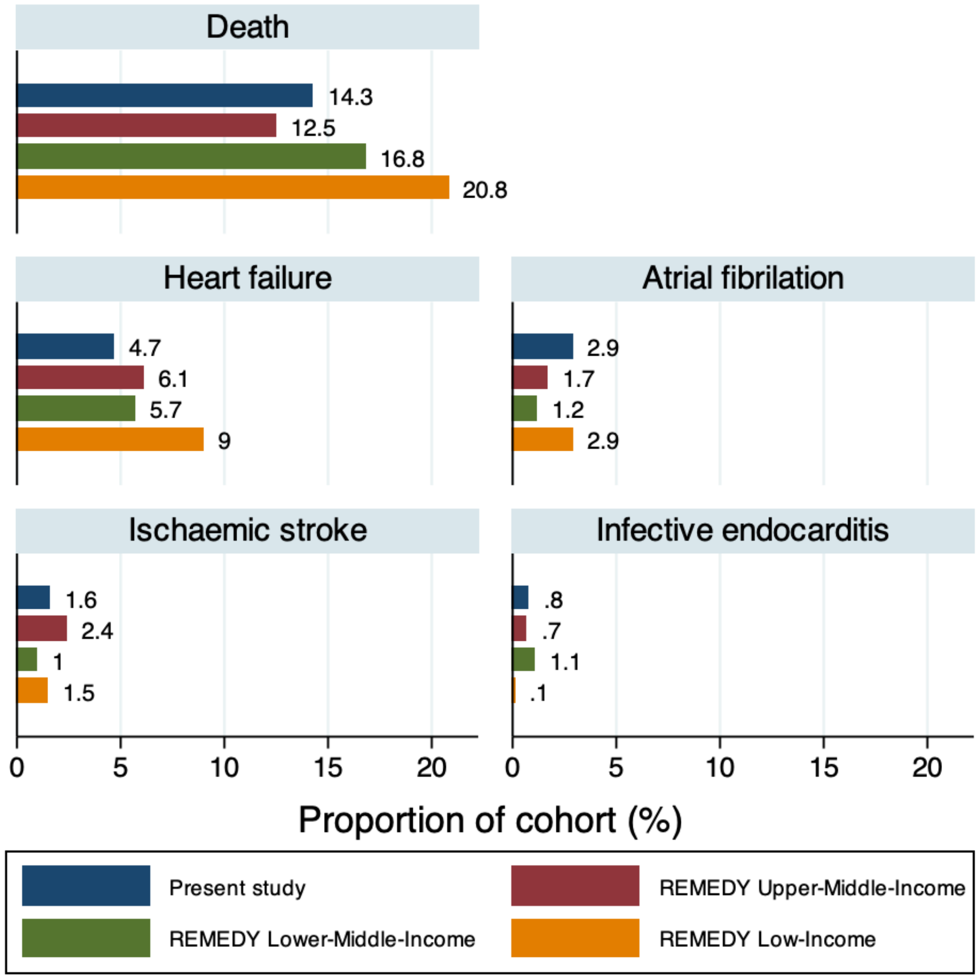

For peer review only - http://bmjopen.bmj.com/site/about/guidelines.xhtml

Table S1: **Diagnostic codes used for this analysis for rheumatic heart disease and its complications.**

**Diagnosis**	ICD-10 Codes*
Rheumatic heart disease	I05-I09
Acute rheumatic fever	I00-I02
Heart failure	I50
Atrial fibrillation	I48
Infective endocarditis	I33
Ischaemic stroke	G46, I63-64, I67.9, I69.3-I69.4, I69.8

*International Statistical Classification of Diseases and Related Health Problems 10th Revision (ICD-10) codes.

For peer review only - http://bmjopen.bmj.com/site/about/guidelines.xhtml

Table S2: **Numbers of individuals and admissions for which diagnostic codes were used in the cohort by age and sex*.** The percentage of individuals and admissions within each age category to which the code applies is also shown.

Diagnosis	Age (yrs)	Admissions, n (%)		Individuals, n (%)	
		Males n=1053	Females n=1830	Males n=892	Females n=1215
RHD	5-14	182 (60)	189 (66)	136 (38)	134 (42)
	15-39	186 (56)	437 (44)	128 (42)	273 (48)
	40-69	128 (30)	205 (38)	100 (44)	141 (46)
ARF	5-14	43 (14)	54 (18)	40 (12)	45 (14)
	15-39	7 (2)	17 (2)	7 (2)	16 (2)
	40-69	1 (0)	1 (0)	1 (0)	1 (0)
Heart failure	5-14	30 (10)	30 (10)	16 (4)	21 (6)
	15-39	90 (28)	93 (10)	53 (16)	66 (12)
	40-69	211 (50)	197 (36)	88 (40)	102 (32)
Atrial fibrillation	5-14	4 (2)	4 (2)	3 (0)	4 (2)
	15-39	79 (24)	74 (8)	46 (14)	51 (8)
	40-69	118 (28)	228 (42)	61 (28)	114 (36)
Ischaemic stroke	5-14	3 (2)	2 (0)	3 (0)	2 (0)
	15-39	16 (4)	24 (2)	15 (4)	20 (4)
	40-69	36 (8)	46 (8)	26 (12)	33 (10)
Endocarditis	5-14	18 (6)	8 (2)	16 (4)	8 (2)
	15-39	20 (6)	17 (2)	20 (6)	14 (2)
	40-69	8 (2)	5 (0)	8 (4)	5 (2)
Ischaemic heart diseas	5-14	6 (2)	2 (0)	6 (2)	2 (0)
	15-39	13 (4)	9 (0)	12 (4)	9 (0)
	40-69	142 (34)	88 (16)	70 (32)	59 (18)
Community acquired pneumonia	5-14	22 (8)	31 (10)	18 (6)	26 (8)
	15-39	29 (8)	43 (4)	26 (8)	35 (6)
	40-69	34 (8)	32 (6)	29 (12)	25 (8)
Diabetes mellitus	5-14	0 (0)	0 (0)	0 (0)	0 (0)
	15-39	2 (0)	6 (0)	1 (0)	4 (0)
	40-69	62 (14)	90 (16)	33 (14)	45 (14)
Obstetric	5-14	0 (0)	4 (2)	0 (0)	3 (0)
	15-39	0 (0)	447 (44)	0 (0)	215 (38)
	40-69	0 (0)	10 (2)	0 (0)	4 (2)

*Sex information missing for nine patients who had a total of 13 admissions.

For peer review only

Table S3: **Clinical events by sex in the national, hospital and maternity cohorts.***

	National (n = 2,116)		Hospital (n = 1,300)		Maternity (n = 210)
	Male (n = 892)	Female (n = 1,215)	Male (n = 547)	Female (n = 748)	Female (n = 210)
Hospital admission, n (%)					
Any complication	252 (28.3)	292 (24.0)	208 (38.0)	241 (32.2)	22 (10.5)
Heart failure	160 (17.9)	189 (15.6)	128 (23.4)	156 (20.9)	11 (5.2)
Atrial fibrillation	109 (12.6)	158 (13.0)	90 (16.5)	125 (16.7)	7 (3.3)
Ischaemic stroke	48 (5.4)	56 (4.6)	37 (6.8)	45 (6.3)	7 (3.3)
Infective endocarditis	45 (5.0)	27 (2.2)	40 (7.3)	23 (3.1)	4 (1.9)
Echocardiography, n (%)					
Moderate disease	103 (11.6)	162 (13.3)	81 (14.8)	137 (18.3)	45 (21.4)
Severe disease	112 (12.6)	147 (12.1)	100 (18.3)	122 (16.3)	29 (13.8)
Mitral stenosis	136 (15.3)	269 (22.1)	121 (22.1)	230 (30.8)	64 (30.5)
Heart valve surgery, n (%)					
Prior to study	32 (3.6)	54 (4.4)	26 (4.8)	38 (5.1)	7 (3.3)
During follow-up	51 (5.7)	75 (6.2)	39 (7.1)	56 (7.5)	10 (4.8)
Date uncertain	31 (3.5)	32 (2.6)	21 (3.8)	21 (2.8)	5 (2.4)
Death during follow-up, n (%)	147 (16.5)	173 (14.2)	113 (20.7)	127 (17.0)	11 (5.2)

*Sex information missing for nine patients in the national cohort and five patients in the hospital cohort.

For peer review only

Table S4: **Cumulative incidence rates per 100 person-years with 95% confidence intervals of nonfatal complications of RHD in the national cohort at various time intervals during the study.**

Age	Sex	Years	Any complication*	Heart failure	Atrial fibrillation	Ischaemic stroke	Infective endocarditis
5-14 years	Male	1-3	17.4 (14.5–21.0)	0.6 (0.3–1.5)	0.1 (0.0–0.9)	0.2 (0.1–1.0)	0.8 (0.3–1.7)
		4-5	8.4 (5.5–12.8)	1.2 (0.5–2.9)	0 (0.0-0.0)	0 (0.0-0.0)	1.2 (0.5–2.9)
		All	14.9 (12.5–17.7)	1.8 (0.4–1.5)	0.1 (0.0–0.6)	0.2 (0.0–0.7)	0.9 (0.5–1.6)
	Female	1-3	16.5 (13.5–20.0)	1.5 (0.8–2.7)	0.3 (0.1–1.1)	0.4 (0.1–1.3)	0.7 (0.3–1.6)
		4-5	11.4 (7.8–16.7)	0.8 (0.3–2.5)	0.3 (0.0–1.8)	0 (0.0-0.0)	0.3 (0.0–1.9)
		All	15.1 (12.7–17.9)	1.3 (0.8–2.1)	0.3 (0.1–0.8)	0.3 (0.1–0.8)	0.5 (0.2–1.2)
15-39 years	Male	1-3	13.2 (11.0–15.9)	3.6 (2.6–5.0)	3.1 (2.1–4.4)	1.7 (1.0–2.7)	0.9 (0.5–1.8)
		4-5	11.4 (8.7–14.9)	2.7 (1.7–4.2)	2.1 (1.2–3.5)	0.4 (0.1–1.3)	2.0 (1.2–3.4)
		All	12.6 (10.8–14.6)	3.2 (2.4–4.2)	2.7 (2.0–3.6)	1.1 (0.7–1.8)	1.4 (0.9–2.1)
	Female	1-3	17.1 (15.1–19.4)	3.0 (2.3–3.9)	1.6 (1.1–2.4)	0.8 (0.4–1.3)	0.7 (0.4–1.2)
		4-5	12.5 (10.1–15.5)	1.5 (0.9–2.4)	1.5 (0.9–2.4)	0.8 (0.4–1.5)	0.3 (0.1–0.8)
		All	15.6 (14.0–17.4)	2.4 (1.9–3.0)	1.6 (1.2–2.1)	0.8 (0.5–1.2)	0.5 (0.3–0.9)
40-69 years	Male	1-3	25.6 (21.4–30.5)	13.5 (10.7–16.8)	8.0 (6.0–10.6)	3.2 (2.1–5.0)	0.6 (0.2–1.7)
		4-5	20.3 (14.8–28.0)	6.5 (4.2–10.3)	6.0 (3.8–9.5)	3.0 (1.6–5.5)	1.7 (0.7–3.7)
		All	24.1 (20.6–28.1)	11.1 (9.1–13.6)	7.3 (5.7–9.3)	3.1 (2.2–4.5)	1.0 (0.5–1.9)
	Female	1-3	22.9 (19.6–26.8)	10.7 (8.7–13.3)	10.7 (8.6–13.2)	3.5 (2.5–5.0)	0.3 (0.1–1.1)
		4-5	17.2 (13.0–22.7)	4.8 (3.2–7.4)	8.6 (6.2–11.9)	2.4 (1.3–4.2)	0.6 (0.2–1.7)
		All	21.2 (18.5–24.3)	8.7 (7.2–10.5)	10.0 (8.3–11.9)	3.1 (2.3–4.2)	0.4 (0.2–0.9)

*At least one hospitalisation for heart failure, stroke, atrial fibrillation or infective endocarditis.

For peer review only - http://bmjopen.bmj.com/site/about/guidelines.xhtml

Table S5: **Hazard ratio estimates with 95% confidence intervals for new nonfatal complications of RHD within the national cohort in a multivariate competing-risks analysis*.**

		Any complication‡	Heart failure	Atrial fibrillation	Ischaemic stroke	Infective endocarditis
Age (yrs)	5-14	1	1	1	1	1
	15-39	**2.77 (1.44–5.32)**	1.98 (0.91–4.30)	**20.4 (5.0–83.4)**	**4.19 (1.48–11.82)**	1.20 (0.67–2.14)
	40-69	**6.78 (3.46–13.28)**	**4.41 (1.97–9.83)**	**77.4 (19.1–313.4)**	**7.26 (2.46–21.4)**	0.77 (0.34–1.74)
Sex	Male	1	1	1	1	1
	Female	**0.62 (0.44–0.87)**	0.65 (0.42–1.01)	1.05 (0.81–1.35)	0.77 (0.52–1.13)	**0.42 (0.26–0.68)**
Ethnicity	Fijian of Indian Descent	1	1	1	1	1
	iTaukei & others	**2.68 (1.79–4.03)**	**1.80 (1.09–2.96)**	**2.0 (1.52–2.61)**	**2.45 (1.52–3.94)**	1.71 (0.97–3.04)
Comorbidity§	Index = 0	1	1	1	1	1
	Index = 1	**5.92 (3.56–9.87**	**3.65 (1.89-7.02)**	**2.92 (2.03–4.21)**	**5.21 (2.59–10.5)**	1.20 (0.36–3.98)
	Index ≥2	**6.41 (4.15–9.89)**	**3.64 (2.00–6.59)**	**2.51 (1.86–3.89)**	**9.14 (5.36–15.6)**	1.97 (0.85–4.54)

*Bold font indicates P<0.05 while underlined font indicates P<0.001. ‡At least one hospitalisation for heart failure, stroke, atrial fibrillation or endocarditis. §Modified Charlson Comorbidity Index[26] calculated without points for heart failure and stroke.

For peer review only - http://bmjopen.bmj.com/site/about/guidelines.xhtml

Table S6: **Population-based rates of hospitalisations for RHD complications per 100,000 person-years in the general population.**

Age (years)	Any complication* Events	Any complication* Rate (95% CI)	Heart failure Events	Heart failure Rate (95% CI)	Atrial fibrillation Events	Atrial fibrillation Rate (95% CI)	Fiji Popn†
0-4	0	-	0	-	0	-	84926
5-9	124	31 (25.8-37)	8	2 (0.9-3.9)	1	0.2 (0-1.4)	80020
10-14	174	41.4 (35.4-48)	21	5 (3.1-7.6)	2	0.5 (0.1-1.7)	84145
15-20	159	39.2 (33.4-45.8)	38	9.4 (6.6-12.9)	15	3.7 (2.1-6.1)	81040
20-24	181	44.3 (38.1-51.2)	34	8.3 (5.8-11.6)	26	6.4 (4.2-9.3)	81722
25-29	188	50.4 (43.4-58.1)	39	10.5 (7.4-14.3)	36	9.6 (6.8-13.4)	74621
30-34	161	49.8 (42.4-58.1)	31	9.6 (6.5-13.6)	28	8.7 (5.8-12.5)	64629
35-39	136	47.3 (39.7-56)	39	13.6 (9.6-18.5)	35	12.2 (8.5-16.9)	57501
40-44	171	59.8 (51.2-69.5)	78	27.3 (21.6-34.1)	48	16.8 (12.4-22.3)	57158
45-49	177	69.5 (59.6-80.5)	91	35.7 (28.8-43.8)	77	30.2 (23.8-37.8)	50960
50-54	147	72.6 (61.3-85.3)	66	32.6 (25.2-41.5)	63	31.1 (23.9-39.8)	40493
55-59	120	76 (63-90.9)	75	47.5 (37.4-59.5)	64	40.5 (31.2-51.8)	31584
60-64	98	80 (65-97.5)	66	53.9 (41.7-68.6)	44	35.9 (26.1-48.2)	24490
65-69	48	56.1 (41.4-74.4)	26	30.4 (19.9-44.6)	36	42.1 (29.5-58.3)	17102
Aged 0–69 years	-	45.4 (43.4-47.5)	-	14.7 (13.6-16.0)	-	11.4 (10.4-12.5)	

*All hospitalisations for heart failure, stroke, atrial fibrillation or endocarditis. †Estimated midpoint population in Fiji.

For peer review only

For peer review only - http://bmjopen.bmj.com/site/about/guidelines.xhtml

Table S6: **Continued**

Age (years)	Ischaemic stroke Events	Ischaemic stroke Rate (95% CI)	Infective endocarditis Events	Infective endocarditis Rate (95% CI)	Fiji Popn*
0-4	0	-	0	-	84926
5-9	0	0 (0-0.9)	7	1.7 (0.7-3.6)	84145
10-14	3	0.7 (0.1-2.1)	9	2.1 (1-4.1)	84145
15-20	4	1 (0.3-2.5)	9	2.2 (1-4.2)	81040
20-24	7	1.7 (0.7-3.5)	3	0.7 (0.2-2.1)	81722
25-29	3	0.8 (0.2-2.3)	10	2.7 (1.3-4.9)	74621
30-34	9	2.8 (1.3-5.3)	9	2.8 (1.3-5.3)	64629
35-39	5	1.7 (0.6-4.1)	8	2.8 (1.2-5.5)	58501
40-44	17	5.9 (3.5-9.5)	8	2.8 (1.2-5.5)	57158
45-49	9	3.5 (1.6-6.7)	3	1.2 (0.2-3.4)	50960
50-54	14	6.9 (3.8-11.6)	4	2 (0.5-5.1)	40493
55-59	8	5.1 (2.2-10)	1	0.6 (0-3.5)	31584
60-64	7	5.7 (2.3-11.8)	0	0 (0-3)	24490
65-69	7	8.2 (3.3-16.9)	0	0 (0-4.3)	17102
Aged 0–69 years		2.2 (1.8-2.7)		1.7 (1.3-2.1)	

*Estimated midpoint population in Fiji.

For peer review only

For peer review only - http://bmjopen.bmj.com/site/about/guidelines.xhtml

Table S7: **Incidence rate ratios estimates with 95% confidence intervals for new nonfatal complications of RHD in the general population in multivariate Poisson regression*.**

		Any complication†	Heart failure	Atrial fibrillation	Ischaemic stroke	Infective endocarditis
Age (yrs)	5-14	1	1	1	1	1
	15-39	2.00 (1.75-2.28)	**4.37 (2.95-6.48)**	**32.8 (10.4-103.6)**	**6.74 (2.04-22.3)**	**1.9 (1.04-3.46)**
	40-69	3.04 (2.66-3.48)	**16.0 (10.9-23.3)**	**130 (41.6-408)**	**25.2 (7.88-80.8)**	1.28 (0.63-2.61)
Sex	Male	1	1	1	1	1
	Female	**1.43 (1.30-1.57)**	0.93 (0.79-1.09)	**1.51 (1.25-1.82)**	1.27 (0.84-1.92)	0.63 (0.39-1.02)
Ethnicity	Fijian of Indian Descent	1	1	1	1	1
	iTaukei & others	**1.39 (1.27-1.53)**	1.18 (1.0-1.38)	**1.30 (1.07-1.59)**	**1.79 (1.13-2.84)**	**2.11 (1.18-3.75)**

*Bold font indicates P<0.05 while underlined font indicates P<0.001. †At least one hospitalisation for heart failure, stroke, atrial fibrillation or endocarditis.

For peer review only

Table S8: **Estimates of years of life lost (YLL), years lived with disability (YLD) and disability adjusted life years (DALYs) attributable to RHD complications per 100,000 person-years in the general population.**

Age	Deaths	Death rate	YLL	YLL rate	YLD	YLD rate	DALYs rate	Fiji popn.*	GBD. std (%)†
0-4	0	0	0	0	0	0	0	84926	10.18
5-9	1.5	0.4	130.1	32.5	43.7	10.9	43.5	80020	9.68
10-14	27.5	6.5	2171.3	516.1	88.9	21.1	537.2	84145	8.95
15-19	28.6	7.1	2116	522.2	81.3	20.1	542.3	81040	8.38
20-24	26.5	6.5	1834.3	448.9	70.4	17.2	466.1	81722	8.02
25-29	24.2	6.5	1554.3	416.6	67.8	18.2	434.8	74621	7.78
30-34	32.7	10.1	1933.1	598.2	58.3	18	616.3	64629	7.33
35-39	34	11.8	1844.8	641.6	54.7	19	660.7	57501	6.78
40-44	33.9	11.8	1670.2	584.4	66.8	23.4	607.8	57158	6.09
45-49	46	18	2042.3	801.5	66.5	26.1	827.6	50960	5.47
50-54	27.7	13.7	1097.3	542	59.9	29.6	571.5	40493	4.87
55-59	30.2	19.1	1053.7	667.2	42.2	26.7	693.9	31584	4.25
60-64	25.6	20.9	774.9	632.8	35.9	29.3	662.2	24490	3.6
65-69	16.2	19	416.1	486.7	18.6	21.8	508.4	17102	2.91
Aged 0–69 years (95% CI)		8.6 (7.7-9.5)		448.9 (442.5-455.4)		18.2 (16.9-19.5)	467.1 (460.5-473.7)		
Age-standardised‡ (95% CI)		9.4 (8.5-10.3)		438.0 (431.8-444.4)		18.4 (17.1-19.7)	456.4 (450.1-462.9)		

*Estimated midpoint population in Fiji. †GBD world population age standard[30]; ‡Study rates for the population aged 65–69 years applied to population aged ≥70 years.

For peer review only

Table S9: **Hazard ratio estimates with 95% confidence intervals for risk of death associated with hospitalisation for nonfatal complications of RHD in the hospital cohort in a multivariate Cox regression analysis.**

Variable	Level	HR (95% CI)	P
Heart failure	Before	1.0	
with or without atrial fib.	After	6.57 (4.76–9.06)	<0.001
Atrial fibrillation	Before	1.0	
without heart failure	After	2.86 (1.75–4.67)	<0.001
Ischaemic stroke	Before	1.0	
	After*	6.56 (3.82–11.2)	<0.001
Infective endocarditis	Before	1.0	
	After*	4.48 (2.22–9.02)	<0.001
Age (yrs)	5-14	1.0	
	15-39	1.40 (0.88–2.24)	0.16
	40-69	1.67 (1.01–2.75)	0.046
Sex	Male	1.0	
	Female	0.83 (0.63–1.09)	0.17
Ethnicity	Fijian of Indian Descent	1.0	
	iTaukei & others	0.78 (0.57–1.07)	0.12
Comorbidity†	Index < 2	1.0	
	Index ≥2	1.49 (1.10–2.03)	0.011
Residence	Urban	1.0	
	Rural	1.18 (0.90–1.54)	0.23

*Hazard within 28 days of admission for complication. †Modified Charlson Comorbidity Index[26] calculated without points for heart failure and stroke.

For peer review only

For peer review only - http://bmjopen.bmj.com/site/about/guidelines.xhtml

STROBE Statement—checklist of items that should be included in reports of observational studies

	Item No.	Recommendation	Page No.	Relevant text from manuscript
**Title and abstract**	1	(*a*) Indicate the study's design with a commonly used term in the title or the abstract	1	
		(*b*) Provide in the abstract an informative and balanced summary of what was done and what was found	2	
		**Introduction**		
Background/rationale	2	Explain the scientific background and rationale for the investigation being reported	4-5	
Objectives	3	State specific objectives, including any prespecified hypotheses	5	
		**Methods**		
Study design	4	Present key elements of study design early in the paper	4	
Setting	5	Describe the setting, locations, and relevant dates, including periods of recruitment, exposure, follow-up, and data collection	6-7	
Participants	6	(*a*) *Cohort study*—Give the eligibility criteria, and the sources and methods of selection of participants. Describe methods of follow-up	6-7	
		(*b*) *Cohort study*—For matched studies, give matching criteria and number of exposed and unexposed	N/A	
Variables	7	Clearly define all outcomes, exposures, predictors, potential confounders, and effect modifiers. Give diagnostic criteria, if applicable	7	
Data sources/ measurement	8*	For each variable of interest, give sources of data and details of methods of assessment (measurement). Describe comparability of assessment methods if there is more than one group	6-7	
Bias	9	Describe any efforts to address potential sources of bias	7-8	
Study size	10	Explain how the study size was arrived at	N/A	

Continued on next page

For peer review only - http://bmjopen.bmj.com/site/about/guidelines.xhtml

Quantitative variables	11	Explain how quantitative variables were handled in the analyses. If applicable, describe which groupings were chosen and why	7-8
Statistical methods	12	(*a*) Describe all statistical methods, including those used to control for confounding	7-8
		(*b*) Describe any methods used to examine subgroups and interactions	7-8
		(*c*) Explain how missing data were addressed	8
		(*d*) *Cohort study*—If applicable, explain how loss to follow-up was addressed	7
		(*e*) Describe any sensitivity analyses	N/A
		**Results**	
Participants	13*	(a) Report numbers of individuals at each stage of study—eg numbers potentially eligible, examined for eligibility, confirmed eligible, included in the study, completing follow-up, and analysed	9-10
		(b) Give reasons for non-participation at each stage	9
		(c) Consider use of a flow diagram	Suppl. Fig. S1
Descriptive data	14*	(a) Give characteristics of study participants (eg demographic, clinical, social) and information on exposures and potential confounders	Table 1
		(b) Indicate number of participants with missing data for each variable of interest	Table 1
		(c) *Cohort study*—Summarise follow-up time (eg, average and total amount)	9
Outcome data	15*	*Cohort study*—Report numbers of outcome events or summary measures over time	10
Main results	16	(*a*) Give unadjusted estimates and, if applicable, confounder-adjusted estimates and their precision (eg, 95% confidence interval). Make clear which confounders were adjusted for and why they were included	10-13
		(*b*) Report category boundaries when continuous variables were categorized	Table 1
		(*c*) If relevant, consider translating estimates of relative risk into absolute risk for a meaningful time period	N/A

Continued on next page

For peer review only

Other analyses	17	Report other analyses done—eg analyses of subgroups and interactions, and sensitivity analyses	13-14
**Discussion**			
Key results	18	Summarise key results with reference to study objectives	14-15
Limitations	19	Discuss limitations of the study, taking into account sources of potential bias or imprecision. Discuss both direction and magnitude of any potential bias	17
Interpretation	20	Give a cautious overall interpretation of results considering objectives, limitations, multiplicity of analyses, results from similar studies, and other relevant evidence	18
Generalisability	21	Discuss the generalisability (external validity) of the study results	15-16
**Other information**			
Funding	22	Give the source of funding and the role of the funders for the present study and, if applicable, for the original study on which the present article is based	20

*Give information separately for cases and controls in case-control studies and, if applicable, for exposed and unexposed groups in cohort and cross-sectional studies.

Note: An Explanation and Elaboration article discusses each checklist item and gives methodological background and published examples of transparent reporting. The STROBE checklist is best used in conjunction with this article (freely available on the Web sites of PLoS Medicine at http://www.plosmedicine.org/, Annals of Internal Medicine at http://www.annals.org/, and Epidemiology at http://www.epidem.com/). Information on the STROBE Initiative is available at www.strobe-statement.org.

For peer review only

For peer review only - http://bmjopen.bmj.com/site/about/guidelines.xhtml
